# Some results on the penalised nematic liquid crystals driven by multiplicative noise: weak solution and maximum principle

**DOI:** 10.1007/s40072-018-0131-z

**Published:** 2019-01-24

**Authors:** Zdzisław Brzeźniak, Erika Hausenblas, Paul André Razafimandimby

**Affiliations:** 1 Department of Mathematics, University of York, Heslington, York, YO10 5DD UK; 2 Department of Mathematics and Informationtechnology, Montanuniversity of Leoben, Franz Josef Strasse 18, 8700 Leoben, Austria; 3 Department of Mathematics and Applied Mathematics, University of Pretoria, Lynwood Road, Pretoria, 0083 South Africa

**Keywords:** Nematic Liquid Crystal, Leslie–Ericksen System, Martingale Solution, Maximum Principle Theorem

## Abstract

In this paper, we prove several mathematical results related to a system of highly nonlinear stochastic partial differential equations (PDEs). These stochastic equations describe the dynamics of penalised nematic liquid crystals under the influence of stochastic external forces. Firstly, we prove the existence of a global weak solution (in the sense of both stochastic analysis and PDEs). Secondly, we show the pathwise uniqueness of the solution in a 2D domain. In contrast to several works in the deterministic setting we replace the Ginzburg–Landau function $\mathbb {1}_{|{\mathbf {n}}|\le 1}(|{\mathbf {n}}|^2-1){\mathbf {n}}$ by an appropriate polynomial $f({\mathbf {n}})$ and we give sufficient conditions on the polynomial *f* for these two results to hold. Our third result is a maximum principle type theorem. More precisely, if we consider $f({\mathbf {n}})=\mathbb {1}_{|d|\le 1}(|{\mathbf {n}}|^2-1){\mathbf {n}}$ and if the initial condition ${\mathbf {n}}_0$ satisfies $|{\mathbf {n}}_0|\le 1$, then the solution ${\mathbf {n}}$ also remains in the unit ball.

## Introduction

Nematic liquid crystal is a state of matter that has properties which are between amorphous liquid and crystalline solid. Molecules of nematic liquid crystals are long and thin, and they tend to align along a common axis. This preferred axis indicates the orientations of the crystalline molecules; hence it is useful to characterize its orientation with a vector field ${\mathbf {n}}$ which is called the **director**. Since its magnitude has no significance, we shall take ${\mathbf {n}}$ as a unit vector. We refer to [10, 15] for a comprehensive treatment of the physics of liquid crystals. To model the dynamics of nematic liquid crystals most scientists use the continuum theory developed by Ericksen [17] and Leslie [28]. From this theory Lin and Liu [29] derived the most basic and simplest form of the dynamical system describing the motion of nematic liquid crystals filling a bounded region ${\mathcal {O}}\subset {\mathbb {R}}^d, d=2,3$. This system is given by

1.1 $$\begin{aligned} {\mathbf {v}}_t+({\mathbf {v}}\cdot \nabla ){\mathbf {v}}-\mu \Delta {\mathbf {v}}+\nabla p&=-\lambda {{\,\mathrm{div}\,}}(\nabla {\mathbf {n}}\odot \nabla {\mathbf {n}}), \text { in } (0,T]\times {\mathcal {O}}\end{aligned}$$

1.2 $$\begin{aligned} \mathrm {div }\;{\mathbf {v}}&=0,\text { in } (0,T]\times {\mathcal {O}}\end{aligned}$$

1.3 $$\begin{aligned} {\mathbf {n}}_t+({\mathbf {v}}\cdot \nabla ){\mathbf {n}}&=\gamma \left( \Delta {\mathbf {n}}+|\nabla {\mathbf {n}}|^2{\mathbf {n}}\right) , \text { in } (0,T]\times {\mathcal {O}} \end{aligned}$$

1.4 $$\begin{aligned} {\mathbf {n}}(0)&={\mathbf {n}}_0, \text { and } {\mathbf {v}}(0)= {\mathbf {v}}_0 \text { in } {\mathcal {O}}\end{aligned}$$

1.5 $$\begin{aligned} |{\mathbf {n}}|^2&=1,\text { on } (0,T]\times {\mathcal {O}}. \end{aligned}$$

Here $p:{\mathbb {R}}^d\rightarrow {\mathbb {R}}$ represents the pressure of the fluid, ${\mathbf {v}}:{\mathbb {R}}^d\rightarrow {\mathbb {R}}^d$ its velocity and ${\mathbf {n}}:{\mathbb {R}}^d \rightarrow {\mathbb {R}}^3$ the liquid crystal molecules director. By the symbol $\nabla {\mathbf {n}}\odot \nabla {\mathbf {n}}$ we mean a $d\times d$-matrix with entries defined by

$$\begin{aligned}{}[\nabla {\mathbf {n}}\odot \nabla {\mathbf {n}}]_{i,j}=\sum _{k=1}^3 \frac{\partial {\mathbf {n}}^{(k)}}{\partial x_i}\frac{\partial {\mathbf {n}}^{(k)}}{\partial x_j},\;\; i,j=1,\dots , d. \end{aligned}$$

We assume that the boundary of ${\mathcal {O}}$ is smooth and equip the system with the boundary conditions

1.6 $$\begin{aligned} {\mathbf {v}}=0 \text { and } \frac{\partial {\mathbf {n}}}{\partial \varvec{\nu }}=0 \text { on } \partial {\mathcal {O}}, \end{aligned}$$

and the initial conditions

1.7 $$\begin{aligned} {\mathbf {v}}(0)={\mathbf {v}}_0 \text { and } {\mathbf {n}}(0)={\mathbf {n}}_0 , \end{aligned}$$

where ${\mathbf {v}}_0$ and ${\mathbf {n}}_0$ are given mappings defined on ${\mathcal {O}}$. Here, the vector field $\varvec{\nu }$ is the unit outward normal to $\partial {\mathcal {O}}$, *i.e.*, at each point *x* of ${\mathcal {O}}$, $\varvec{\nu }(x)$ is perpendicular to the tangent space $T_x\partial {\mathcal {O}}$, of length 1 and facing outside of ${\mathcal {O}}$.

Although the system (1.1)–(1.6) is the most basic and simplest form of equations from the Ericksen–Leslie continuum theory, it retains the most physical significance of the Nematic liquid crystals. Moreover, it offers several interesting mathematical problems. In fact, on one hand, two of the main mathematical difficulties related to the system (1.1)–(1.6) are non-parabolicity of Eq. (1.3) and high nonlinearity of the term $\mathrm {div }\;\sigma ^E= -\mathrm {div }\;( \nabla {\mathbf {n}}\odot \nabla {\mathbf {n}})$. The non-parabolicity follows from the fact that

1.8 $$\begin{aligned} \Delta {\mathbf {n}}+|\nabla {\mathbf {n}}|^2{\mathbf {n}}={\mathbf {n}}\times (\Delta {\mathbf {n}}\times {\mathbf {n}}), \end{aligned}$$

so that the linear term $\Delta {\mathbf {n}}$ in (1.3) is only a tangential part of the full Laplacian. Here we have denoted the vector product by $\times$. The term $\mathrm {div }\;( \nabla {\mathbf {n}}\odot \nabla {\mathbf {n}})$ makes the problem (1.1)–(1.6) a fully nonlinear and constrained system of PDEs coupled via a quadratic gradient nonlinearity. On the other hand, a number of challenging questions about the solutions to Navier–Stokes equations (NSEs) and Geometric Heat equation (GHE) are still open.

In 1995, Lin and Liu [29] proposed an approximation of the system (1.1)–(1.6) to relax the constraint $|{\mathbf {n}}|^2=1$ and the gradient nonlinearity $|\nabla {\mathbf {n}}|^2{\mathbf {n}}$. More precisely, they studied the following system of equations

1.9 $$\begin{aligned} {\mathbf {v}}_t+({\mathbf {v}}\cdot \nabla ){\mathbf {v}}-\mu \Delta {\mathbf {v}}+\nabla p&=-\lambda {{\,\mathrm{div}\,}}(\nabla {\mathbf {n}}\odot \nabla {\mathbf {n}}), \text { in } (0,T]\times {\mathcal {O}} \end{aligned}$$

1.10 $$\begin{aligned} \mathrm {div }\;{\mathbf {v}}&=0, \text { in } [0,T]\times {\mathcal {O}}\end{aligned}$$

1.11 $$\begin{aligned} {\mathbf {n}}(0)&={\mathbf {n}}_0 \text { and }{\mathbf {v}}(0)={\mathbf {v}}_0 \text { in } {\mathcal {O}}, \end{aligned}$$

1.12 $$\begin{aligned} {\mathbf {n}}_t+({\mathbf {v}}\cdot \nabla ){\mathbf {n}}&=\gamma \left( \Delta {\mathbf {n}}-\frac{1}{\varepsilon ^2} (|{\mathbf {n}}|^2-1){\mathbf {n}}\right) \text { in } (0,T]\times {\mathcal {O}}, \end{aligned}$$

where $\varepsilon >0$ is an arbitrary constant.

Problem (1.9)–(1.12) with boundary conditions (1.6) is much simpler than (1.1)–(1.5) with (1.6), but it offers several difficult mathematical problems. Since the pioneering work [29] the systems (1.9)–(1.12) and (1.1)–(1.5) have been the subject of intensive mathematical studies. We refer, among others, to [13, 19, 21, 29, 31–33, 42] and references therein for the relevant results. We also note that more general Ericksen–Leslie systems have been recently studied, see, for instance, [9, 22, 23, 25, 30, 47, 48] and references therein.

In this paper, we are interested in the mathematical analysis of a stochastic version of problem (1.9)–(1.12). Basically, we will investigate a system of stochastic evolution equations which is obtained by introducing appropriate noise term in (1.1)–(1.5). In contrast to the unpublished manuscript [7] we replace the bounded Ginzburg–Landau function $\mathbb {1}_{|{\mathbf {n}}|\le 1}(|{\mathbf {n}}|^2-1){\mathbf {n}}$ in the coupled system by an appropriate polynomial function $f({\mathbf {n}})$. More precisely, we set $\mu =\lambda =\gamma =1$ and we consider cylindrical Wiener processes $W_1$ on a separable Hilbert space $\mathrm {K}_1$ and a standard real-valued Brownian motion $W_2$. We assume that $W_1$ and $W_2$ are independent. We consider the problem

1.13 $$\begin{aligned} d{\mathbf {v}}(t)+\big [({\mathbf {v}}(t)\cdot \nabla ){\mathbf {v}}(t)-\Delta {\mathbf {v}}(t)+\nabla p\big ]dt&= -{{\,\mathrm{div}\,}}(\nabla {\mathbf {n}}(t)\odot \nabla {\mathbf {n}}(t))dt+ S({\mathbf {v}}(t)) dW_1(t), \end{aligned}$$

1.14 $$\begin{aligned} \mathrm {div }\;{\mathbf {v}}(t)&= 0, \end{aligned}$$

1.15 $$\begin{aligned} d{\mathbf {n}}(t)+({\mathbf {v}}(t)\cdot \nabla ){\mathbf {n}}(t)dt&= \big [\Delta {\mathbf {n}}(t)-f({\mathbf {n}})\big ]dt+({\mathbf {n}}(t)\times {\mathbf {h}})\circ dW_2(t), \end{aligned}$$

1.16 $$\begin{aligned} {\mathbf {v}}&=0 \text { and } \frac{\partial {\mathbf {n}}}{\partial \varvec{\nu }}=0 \text { on } \partial {\mathcal {O}}, \end{aligned}$$

1.17 $$\begin{aligned} {\mathbf {v}}(0)&={\mathbf {v}}_0 \text { and } {\mathbf {n}}(0)={\mathbf {n}}_0, \end{aligned}$$

where ${\mathbf {h}}:{\mathbb {R}}^d \rightarrow {\mathbb {R}}^3$ is a given function, $({\mathbf {n}}(t)\times {\mathbf {h}})\circ dW_2(t)$ is understood in the Stratonovich sense and *f* is a polynomial function and the above system holds in ${\mathcal {O}}_T:=(0,T]\times {\mathcal {O}}$. We will give more details about the polynomial *f* later on.

Our work is motivated by the importance of external perturbation on the dynamics of the director field ${\mathbf {n}}$. Indeed, an essential property of nematic liquid crystals is that its director field ${\mathbf {n}}$ can be easily distorted. However, it can also be aligned to form a specific pattern under some external perturbations. This pattern formation occurs when a threshold value of the external perturbations is attained; this is the so-called Fréedericksz transition. Random external perturbations change a little bit the threshold value for the Fréedericksz transition. For example, it has been found that with the fluctuation of the magnetic field the relaxation time of an unstable state diminishes, *i.e.*, the time for a noisy system to leave an unstable state is much shorter than the unperturbed system. For these results, we refer, among others, to [24, 40, 41] and references therein. In all of these works, the effect of the hydrodynamic flow has been neglected. However, it is pointed out in [15, Chapter 5] that the fluid flow disturbs the alignment and conversely a change in the alignment will induce a flow in the nematic liquid crystal. Hence, for a full understanding of the effect of fluctuating magnetic field on the behavior of the liquid crystals one needs to take into account the dynamics of ${\mathbf {n}}$ and ${\mathbf {v}}$. To initiate this kind of investigation we propose a mathematical study of (1.13)–(1.15) which basically describes an approximation of the system governing the nematic liquid crystals under the influence of fluctuating external forces.

In the present paper, we prove some results that are the stochastic counterparts of some of those obtained by Lin and Liu in [29]. Our results can be described as follows. In Sect. 3 we establish the existence of global martingale solutions (weak in the PDEs sense). To prove this result, we first find a suitable finite dimensional Galerkin approximation of system (1.13)–(1.15), which can be solved locally in time. Our choice of the approximation yields the global existence of the approximating solutions $({\mathbf {v}}_m,{\mathbf {n}}_m)$. For this purpose, we derive several significant global a priori estimates in higher order Sobolev spaces involving the following two energy functionals

$$\begin{aligned} {\mathcal {E}}_1({\mathbf {n}},t):= \Vert {\mathbf {n}}(t)\Vert ^q+q \int _0^t \Vert {\mathbf {n}}(s)\Vert ^{q-2} \Vert \nabla {\mathbf {n}}(s)\Vert ^2 ds + q \int _0^t \Vert {\mathbf {n}}(s)\Vert ^{q-2} \Vert {\mathbf {n}}(s)\Vert ^{2N+2}_{{\mathbf {L}}^{2N+2}} ds \end{aligned}$$

and

$$\begin{aligned} {\mathcal {E}}_2({\mathbf {v}},{\mathbf {n}},t):= & {} \Vert {\mathbf {v}}(t)\Vert ^{2}+{\tilde{\ell }} \Vert {\mathbf {n}}(t) \Vert ^2+\Vert \nabla {\mathbf {n}}(t)\Vert ^{2}+\int _{\mathcal {O}}F({\mathbf {n}}(t,x) dx \\&+ \left( \int _0^{t} \Vert \nabla {\mathbf {v}}(s)\Vert ^2+ \Vert \Delta {\mathbf {n}}(s)-f({\mathbf {n}}(s))\Vert ^2\right) ds. \end{aligned}$$

Here $F(\cdot )$ is the antiderivative of *f* such that $F(0)=0$ and ${\tilde{\ell }}>0$ is a certain constant. These global a priori estimates, the proofs of which are non-trivial and require long and tedious calculation, are very crucial for the proof of the tightness of the family of distributions $\{({\mathbf {v}}_m,{\mathbf {n}}_m):m\in {\mathbb {N}}\}$, where $({\mathbf {v}}_m,{\mathbf {n}}_m)$ is the solution of the Galerkin approximation in certain appropriate topological spaces such as $L^2(0,T;{\mathbb {L}}^2({\mathcal {O}}) \times {\mathbf {H}}^1({\mathcal {O}}))$. This tightness result along Prokhorov’s theorem and Skorokhod’s representation theorem will enable us to construct a new probability space on which we also find a new sequence of processes $(\bar{{\mathbf {v}}}_m,\bar{{\mathbf {n}}}_m, {\bar{W}}_1^m,{\bar{W}}_2^m)$ of solutions of the Galerkin equations. This new sequence is proved to converge to a system $({\mathbf {v}},{\mathbf {n}},{\bar{W}}_1, {\bar{W}}_2)$ which along with the new probability space will form our weak martingale solution. To close the first part of our results we show that the weak martingale solution is pathwise unique in the 2-D case. We prove a maximum principle type theorem in Sect. 5. More precisely, if we consider $f({\mathbf {n}})=\mathbb {1}_{|d|\le 1}(|{\mathbf {n}}|^2-1){\mathbf {n}}$ instead and if the initial condition ${\mathbf {n}}_0$ satisfies $|{\mathbf {n}}_0|\le 1$, then the solution ${\mathbf {n}}$ also remains in the unit ball. In contrast to the deterministic case, this result does not follow in a straightforward way from well-known results. Here the method of proofs are based on the blending of ideas from [11, 16].

To the best of our knowledge, our work is the first mathematical work, which studies the existence and uniqueness of a weak martingale solution of system (1.13)–(1.15). Under the assumption that $f(\cdot )$ is a bounded function, the authors proved in the unpublished manuscript [7] that the system (1.13)–(1.15) has a maximal strong solution which is global for the 2D case. Therefore, the present article is a generalization of [7] in the sense that we allow $f(\cdot )$ to be an unbounded polynomial function.

The organization of the present article is as follows. In Sect. 2 we introduce the notations that are frequently used throughout this paper. In the same section, we also state and prove some useful lemmata. By using the scheme, we outlined above we show in Sect. 3 that (1.13)–(1.15) admits a weak martingale solution which is pathwise unique in the two-dimensional case. The existence results rely on the derivation of several crucial estimates for the approximating solutions. These uniform estimates are proved in Sect. 4. In Sect. 5 a maximum principle type theorem is proved when $f({\mathbf {n}})=\mathbb {1}_{|{\mathbf {n}}|\le 1}(|{\mathbf {n}}|^2-1){\mathbf {n}}$. In “Appendix” section we recall or prove several crucial estimates about the nonlinear terms of the system (1.13)–(1.15).

## Functional spaces and preparatory lemma

### Functional spaces and linear operators

Let $d\in \{2,3\}$ and assume that ${\mathcal {O}}\subset {\mathbb {R}}^d$ is a bounded domain with boundary $\partial {\mathcal {O}}$ of class ${\mathcal {C}}^\infty$. For any $p\in [1,\infty )$ and $k\in {\mathbb {N}}$, $L^p({\mathcal {O}})$ and $\mathrm {W}^{k,p}({\mathcal {O}})$ are the well-known Lebesgue and Sobolev spaces, respectively, of ${\mathbb {R}}$-valued functions. The spaces of functions ${\mathbf {v}}:{\mathbb {R}}^d\rightarrow {\mathbb {R}}^d$ (resp. ${\mathbf {n}}:{\mathbb {R}}^d \rightarrow {\mathbb {R}}^3$) such that each component of ${\mathbf {v}}$ (resp. ${\mathbf {n}}$) belongs to $L^p({\mathcal {O}})$ or to $\mathrm {W}^{k,p}({\mathcal {O}})$ are denoted by ${\mathbb {L}}^p({\mathcal {O}})$ or by ${\mathbb {W}}^{k,p}({\mathcal {O}})$ (resp. by ${\mathbf {L}}^p({\mathcal {O}})$ or by ${\mathbf {W}}^{k,p}({\mathcal {O}})$). For $p=2$ the function space ${\mathbb {W}}^{k,2}({\mathcal {O}})$ is denoted by ${\mathbb {H}}^k$ and its norm is denoted by $\Vert {\mathbf {u}}\Vert _k$. The usual scalar product on ${\mathbb {L}}^2$ is denoted by $\langle u,v\rangle$ for $u,v\in {\mathbb {L}}^2$ and its associated norm is denoted by $||u||$, $u\in {\mathbb {L}}^2$. By ${\mathbb {H}}^1_0$ we mean the space of functions in ${\mathbb {H}}^1$ that vanish on the boundary on ${\mathcal {O}}$; ${\mathbb {H}}^1_0$ is a Hilbert space when endowed with the scalar product induced by that of ${\mathbb {H}}^1$. We understand that the same remarks hold for the spaces and ${\mathbf {W}}^{k,p}$, ${\mathbf {H}}^1$, ${\mathbf {L}}^{2}$ and so on. We will also understand that the norm of ${\mathbf {H}}^k$ (resp. ${\mathbf {L}}^2$) is also denoted by $||\cdot ||_{k}$ (resp. $||\cdot ||$).

We now introduce the following spaces

$$\begin{aligned} {\mathcal {V}}&=\left\{ {\mathbf {u}}\in {\mathcal {C}}_{c}^{\infty }({\mathcal {O}},{\mathbb {R}}^d)\,\,\text {such that} \,\,{{\,\mathrm{div}\,}}{\mathbf {u}}=0\right\} \\ \mathrm {V}&=\,\,\text {closure of }{\mathcal {V}}\text { in }\,\,{\mathbb {H}}_0^{1}({\mathcal {O}}) \\ \mathrm {H}&=\,\,\text {closure of }{\mathcal {V}}\text { in }\,\,{\mathbb {L}}^{2}({\mathcal {O}}). \end{aligned}$$

We endow $\mathrm {H}$ with the scalar product and norm of ${\mathbb {L}}^2$. As usual we equip the space $\mathrm {V}$ with the the scalar product $\langle \nabla {\mathbf {u}}, \nabla {\mathbf {v}}\rangle$ which, owing to the Poincaré inequality, is equivalent to the ${\mathbb {H}}^1({\mathcal {O}})$-scalar product.

Let $\Pi : {\mathbb {L}}^2 \rightarrow \mathrm {H}$ be the Helmholtz-Leray projection from ${\mathbb {L}}^2$ onto $\mathrm {H}$. We denote by $\mathrm {A}=-\Pi \Delta$ the Stokes operator with domain $D(\mathrm {A})=\mathrm {V}\cap {\mathbb {H}}^2$. It is well-known (see for e.g. [45, Chapter I, Section 2.6]) that there exists an orthonormal basis $(\varphi _i)_{i=1}^\infty$ of $\mathrm {H}$ consisting of the eigenfunctions of the Stokes operator $\mathrm {A}$. For $\beta \in [0,\infty )$, we denote by $\mathrm {V}_\beta$ the Hilbert space $D(\mathrm {A}^\beta )$ endowed with the graph inner product. The Hilbert space $\mathrm {V}_\beta =D(\mathrm {A}^\beta )$ for $\beta \in (-\infty , 0)$ can be defined by standard extrapolation methods. In particular, the space $D(\mathrm {A}^{-\beta })$ is the dual of $\mathrm {V}_\beta$ for $\beta \ge 0$. Moreover, for every $\beta ,\delta \in {\mathbb {R}}$ the mapping $\mathrm {A}^\delta$ is a linear isomorphism between $\mathrm {V}_\beta$ and $\mathrm {V}_{\beta -\delta }$. It is also well-known that $\mathrm {V}_\frac{1}{2} = \mathrm {V},$ see [12, page 33].

The Neumann Laplacian acting on ${\mathbb {R}}^3$-valued function will be denoted by $\mathrm {A}_1$, that is,

2.1 $$\begin{aligned} \begin{aligned} D(\mathrm {A}_1)&:=\biggl \{{\mathbf {u}}\in {\mathbf {H}}^2: \frac{\partial {\mathbf {u}}}{\partial \varvec{\nu }}=0 \text { on } \partial {\mathcal {O}}\biggr \},\\ \mathrm {A}_1{\mathbf {u}}&:=-\sum _{i=1}^d \frac{\partial ^2 {\mathbf {u}}}{\partial x_i^2},\;\;\; {\mathbf {u}}\in D(\mathrm {A}_1). \end{aligned} \end{aligned}$$

It can also be shown, see e.g. [20, Theorem 5.31], that $\hat{\mathrm {A}}_1=I+\mathrm {A}_1$ is a definite positive and self-adjoint operator in the Hilbert space ${\mathbf {L}}^2:= {\mathbf {L}}^2({\mathcal {O}})$ with compact resolvent. In particular, there exists an ONB $(\phi _k)_{k=1}^\infty$ of ${\mathbf {L}}^2$ and an increasing sequence $\big (\lambda _k\big )_{k=1}^\infty$ with $\lambda _1=0$ and $\lambda _k\nearrow \infty$ as $k\nearrow \infty$ (the eigenvalues of the Neumann Laplacian $\mathrm {A}_1$) such that $\mathrm {A}_1\phi _k=\lambda _k \phi _k$ for any $j\in {\mathbb {N}}$.

For any $\alpha \in [0,\infty )$ we denote by ${\mathbf {X}}_\alpha =D(\hat{\mathrm {A}}_1^{\alpha })$, the domain of the fractional power operator $\hat{\mathrm {A}}_1^{\alpha }$. We have the following characterization of the spaces ${\mathbf {X}}_\alpha$,

2.2 $$\begin{aligned} {\mathbf {X}}_{\alpha }=\left\{ {\mathbf {u}}= \sum _{k\in {\mathbb {N}}} u_k \phi _k: \sum _{k \in {\mathbb {N}}} (1+\lambda _k)^{2\alpha } |u_k|^2<\infty \right\} . \end{aligned}$$

It can be shown that ${\mathbf {X}}_\alpha \subset {\mathbf {H}}^{2\alpha }$, for all $\alpha \ge 0$ and ${\mathbf {X}}:={\mathbf {X}}_{\frac{1}{2}}={\mathbf {H}}^1$, see, for instance, [46, Sections 4.3.3 and 4.9.2].

For a fixed ${\mathbf {h}}\in {\mathbf {L}}^\infty$ we define a bounded linear operator *G* from ${\mathbf {L}}^2$ into itself by

$$\begin{aligned} G: {\mathbf {L}}^2 \ni {\mathbf {n}}\mapsto {\mathbf {n}}\times {\mathbf {h}}\in {\mathbf {L}}^2. \end{aligned}$$

It is straightforward to check that there exists a constant $C>0$ such that

$$\begin{aligned} \Vert G({\mathbf {n}})\Vert \le C \Vert {\mathbf {h}}\Vert _{{\mathbf {L}}^\infty } \Vert {\mathbf {n}}\Vert , \text { for any } {\mathbf {n}}\in {\mathbf {L}}^2. \end{aligned}$$

Given two Hilbert spaces *K* and *H*, we denote by ${\mathcal {L}}(K,H)$ and ${\mathcal {T}}_2(K,H)$ the space of bounded linear operators and the Hilbert space of all Hilbert–Schmidt operators from *K* to *H*, respectively. For $K=H$ we just write ${\mathcal {L}}(K)$ instead of ${\mathcal {L}}(K,K)$.

### The nonlinear terms

Throughout this paper ${\mathbf {B}}^*$ denotes the dual space of a Banach space ${\mathbf {B}}$. We also denote by $\langle \Psi , {\mathbf {b}}\rangle _{{\mathbf {B}}^*,{\mathbf {B}}}$ the value of $\Psi \in {\mathbf {B}}^*$ on ${\mathbf {b}}\in {\mathbf {B}}$.

We define a trilinear form $b(\cdot , \cdot ,\cdot )$ by

$$\begin{aligned} b({\mathbf {u}},{\mathbf {v}},{\mathbf {w}})=\sum _{i,j=1}^d \int _{\mathcal {O}}{\mathbf {u}}^{(i)}\frac{\partial {\mathbf {v}}^{(j)}}{\partial x_i}{\mathbf {w}}^{(j)} dx,\,\, {\mathbf {u}}\in {\mathbb {L}}^p ,{\mathbf {v}}\in {\mathbb {W}}^{1,q}, \text { and } {\mathbf {w}}\in {\mathbb {L}}^r, \end{aligned}$$

with numbers $p,q,r \in [1,\infty ]$ satisfying

$$\begin{aligned} \frac{1}{p} +\frac{1}{q} +\frac{1}{r}\le 1. \end{aligned}$$

Here $\partial _{x_i}=\frac{\partial }{\partial x_i}$ and $\phi ^{(i)}$ is the *i*-th entry of any vector-valued $\phi$. Note that in the above definition we can also take ${\mathbf {v}}\in {\mathbf {W}}^{1,q}$ and ${\mathbf {w}}\in {\mathbf {L}}^r$, but in this case we have to take the sum over *j* from $j=1$ to $j=3$.

The mapping *b* is the trilinear form used in the mathematical analysis of the Navier–Stokes equations, see for instance [45, Chapter II, Section 1.2]. It is well known, see [45, Chapter II, Section 1.2], that one can define a bilinear mapping *B* from $\mathrm {V}\times \mathrm {V}$ with values in $\mathrm {V}^*$ such that

2.3 $$\begin{aligned} \langle B({\mathbf {u}},{\mathbf {v}}),{\mathbf {w}}\rangle _{\mathrm {V}^*,\mathrm {V}}=b({\mathbf {u}},{\mathbf {v}},{\mathbf {w}})\text { for } {\mathbf {w}}\in \mathrm {V},\text { and } {\mathbf {u}}, {\mathbf {v}}\in {\mathbb {H}}^1. \end{aligned}$$

In a similar way, we can also define a bilinear mapping ${\tilde{B}}$ defined on ${\mathbb {H}}^1 \times {\mathbf {H}}^1$ with values in $({\mathbf {H}}^1)^*$ such that

2.4 $$\begin{aligned} \langle {\tilde{B}}({\mathbf {u}},{\mathbf {v}}),{\mathbf {w}}\rangle _{({\mathbf {H}}^1)^*,{\mathbf {H}}^1}=b({\mathbf {u}},{\mathbf {v}},{\mathbf {w}})\,\, \text { for any } {\mathbf {u}}\in {\mathbb {H}}^1, \,\, {\mathbf {v}}, \,\,{\mathbf {w}}\in {\mathbf {H}}^1. \end{aligned}$$

Well-known properties of *B* and ${\tilde{B}}$ will be given in the “Appendix” section.

Let ${\mathfrak {m}}$ be the trilinear form defined by

2.5 $$\begin{aligned} {\mathfrak {m}}({\mathbf {n}}_1, {\mathbf {n}}_2,{\mathbf {u}})= -\sum _{i,j=1}^d\sum _{k=1}^3 \int _{\mathcal {O}}\partial _{x_i}{\mathbf {n}}_1^{(k)} \partial _{x_j}{\mathbf {n}}_2^{(k)} \partial _{x_j}{\mathbf {u}}^{(i)} \,dx \end{aligned}$$

for any ${\mathbf {n}}_1\in {\mathbf {W}}^{1,p}$, ${\mathbf {n}}_2\in {\mathbf {W}}^{1,q}$ and ${\mathbf {u}}\in {\mathbb {W}}^{1,r}$ with $r,\,p,\, q\in (1,\infty )$ satisfying

$$\begin{aligned} \frac{1}{p} +\frac{1}{q}+\frac{1}{r}\le 1. \end{aligned}$$

Since $d\le 4$, the integral in (2.5) is well defined for ${\mathbf {n}}_1, {\mathbf {n}}_2 \in {\mathbf {H}}^2$ and ${\mathbf {u}}\in \mathrm {V}$. We have the following lemma.

#### Lemma 2.1

Let $d\in [1,4]$. Then, there exist a constant $C>0$ such that

2.6 $$\begin{aligned} |{\mathfrak {m}}({\mathbf {n}}_1, {\mathbf {n}}_2, {\mathbf {u}})|\le C \Vert \nabla {\mathbf {n}}_1 \Vert ^{1-\frac{d}{4}} \Vert \nabla ^2 {\mathbf {n}}_1\Vert ^{\frac{d}{4}} \Vert \nabla {\mathbf {n}}_2\Vert ^{1-\frac{d}{4}} \Vert \nabla ^2 {\mathbf {n}}_2\Vert ^{\frac{d}{4}} \Vert \nabla {\mathbf {u}}\Vert , \end{aligned}$$

for any ${\mathbf {n}}_1, {\mathbf {n}}_2\in {\mathbf {H}}^2$ and ${\mathbf {u}}\in \mathrm {V}$.

#### Proof of Lemma 2.1

From (2.5) and Hölder’s inequality we derive that

$$\begin{aligned} \begin{aligned} |{\mathfrak {m}}({\mathbf {n}}_1,{\mathbf {n}}_2,{\mathbf {u}})|\le \int _{{\mathcal {O}}} |\nabla {\mathbf {n}}_1||\nabla {\mathbf {n}}_2| |\nabla {\mathbf {u}}| dx. \end{aligned} \end{aligned}$$

The above integral is well-defined since $\nabla {\mathbf {n}}_i\in {\mathbf {L}}^{\frac{2d}{d-2}}, \,i=1,\, 2$, $\nabla {\mathbf {u}}\in {\mathbb {L}}^2$ and $\frac{d-2}{d}+\frac{1}{2} \le 1$ for $d\le 4.$ When $d=2$ we replace $2d/(d-2)$ by any $q\in [4,\infty )$. Note that for $d\le 4$ we have $|\nabla {\mathbf {n}}_i| \in {\mathbf {L}}^{4}$, $i=1,2$. Hence

$$\begin{aligned} |{\mathfrak {m}}({\mathbf {n}}_1, {\mathbf {n}}_2, {\mathbf {u}})|\le C \Vert \nabla {\mathbf {n}}_1\Vert _{{\mathbb {L}}^4} \Vert \nabla {\mathbf {n}}_2\Vert _{{\mathbb {L}}^4} \Vert \nabla {\mathbf {u}}\Vert . \end{aligned}$$

This last estimate and Gagliardo–Nirenberg’s inequality (6.1) lead us to

2.7 $$\begin{aligned} |{\mathfrak {m}}({\mathbf {n}}_1,{\mathbf {n}}_2, {\mathbf {u}})|\le C \Vert \nabla {\mathbf {n}}_1 \Vert ^{1-\frac{d}{4}} \Vert \nabla ^2 {\mathbf {n}}_1\Vert ^{\frac{d}{4}} \Vert \nabla {\mathbf {n}}_2\Vert ^{1-\frac{d}{4}} \Vert \nabla ^2 {\mathbf {n}}_2\Vert ^{\frac{d}{4}} \Vert \nabla {\mathbf {u}}\Vert . \end{aligned}$$

This concludes the proof of our claim. $\square$

The above result tells us that the mapping $\mathrm {V}\ni {\mathbf {u}}\mapsto {\mathfrak {m}}({\mathbf {n}}_1, {\mathbf {n}}_2,{\mathbf {u}})$ is an element of ${\mathcal {L}}(\mathrm {V}, {\mathbb {R}})$ whenever ${\mathbf {n}}_1, {\mathbf {n}}_2\in {\mathbf {H}}^2$. Now, we state and prove the following proposition.

#### Proposition 2.2

Let $d\in [1,4]$. There exists a bilinear operator *M* defined on ${\mathbf {H}}^2\times {\mathbf {H}}^2$ taking values in $\mathrm {V}^*$ such that for any ${\mathbf {n}}_1, \, {\mathbf {n}}_2 \in {\mathbf {H}}^2$

2.8 $$\begin{aligned} \langle M({\mathbf {n}}_1,{\mathbf {n}}_2), {\mathbf {u}}\rangle _{\mathrm {V}^*,\mathrm {V}}= {\mathfrak {m}}({\mathbf {n}}_1,{\mathbf {n}}_2,{\mathbf {u}}) \;\; {\mathbf {u}}\in \mathrm {V}. \end{aligned}$$

Furthermore, there exists a constant $C>0$ such that

2.9 $$\begin{aligned} \Vert M({\mathbf {n}}_1,{\mathbf {n}}_2)\Vert _{\mathrm {V}^*} \le C \Vert \nabla {\mathbf {n}}_1 \Vert ^{1-\frac{d}{4}} \Vert \nabla ^2 {\mathbf {n}}_1\Vert ^{\frac{d}{4}} \Vert \nabla {\mathbf {n}}_2\Vert ^{1-\frac{d}{4}} \Vert \nabla ^2 {\mathbf {n}}_2\Vert ^{\frac{d}{4}}, \end{aligned}$$

for any ${\mathbf {n}}_1, {\mathbf {n}}_2\in {\mathbf {H}}^2$. We also have the following identity

2.10 $$\begin{aligned} \langle {\tilde{B}}({\mathbf {v}},{\mathbf {n}}), \mathrm {A}_1{\mathbf {n}}\rangle =-\langle M({\mathbf {n}},{\mathbf {n}}), {\mathbf {v}}\rangle _{\mathrm {V}^*,\mathrm {V}}, \text { for any } {\mathbf {v}}\in \mathrm {V}, {\mathbf {n}}\in D(\mathrm {A}_1). \end{aligned}$$

#### Proof

The first part and (2.9) follow from Lemma 2.1.

To prove (2.10) we first note that $\langle {\tilde{B}}({\mathbf {v}},{\mathbf {n}}_2), \mathrm {A}_1{\mathbf {n}}_1\rangle =b({\mathbf {v}},{\mathbf {n}}_2,\mathrm {A}_1{\mathbf {n}}_1)$ is well-defined for any ${\mathbf {v}}\in \mathrm {V}, {\mathbf {n}}_1, {\mathbf {n}}_2 \in D(\mathrm {A}_1).$ Thus, taking into account that ${\mathbf {v}}$ is divergence free and vanishes on the boundary we can perform an integration-by-parts and deduce that

$$\begin{aligned} \langle B({\mathbf {v}},{\mathbf {n}}),\mathrm {A}_1{\mathbf {n}})\rangle&= -\int _{\mathcal {O}}{\mathbf {v}}^{(i)} \frac{\partial {\mathbf {n}}^{(k)}}{\partial x_i}\frac{\partial ^2 {\mathbf {n}}^{(k)}}{\partial x_l \partial x_l} dx\\&= \int _{\mathcal {O}}\frac{\partial {\mathbf {v}}^{(i)}}{\partial x_l} \frac{\partial {\mathbf {n}}^{(k)}}{\partial x_i} \frac{\partial {\mathbf {n}}^{(k)}}{\partial x_l} dx- \int _{\mathcal {O}}{\mathbf {v}}^{(i)} \frac{\partial ^2 {\mathbf {n}}^{(k)}}{\partial x_i\partial x_l} \frac{\partial {\mathbf {n}}^{(k)}}{\partial x_l} dx\\&=-\int _{\mathcal {O}}\frac{\partial {\mathbf {v}}^{(i)}}{\partial x_l} \frac{\partial {\mathbf {n}}^{(k)}}{\partial x_i} \frac{\partial {\mathbf {n}}^{(k)}}{\partial x_l} dx - \frac{1}{2} \int _{\mathcal {O}}{\mathbf {v}}^{(i)} \frac{\partial |\nabla {\mathbf {n}}|^2}{\partial x_i}dx\\&= \int _{\mathcal {O}}\frac{\partial {\mathbf {v}}^{(i)}}{\partial x_l} \frac{\partial {\mathbf {n}}^{(k)}}{\partial x_i} \frac{\partial {\mathbf {n}}^{(k)}}{\partial x_l} dx\\&= -{\mathfrak {m}}({\mathbf {n}},{\mathbf {n}},{\mathbf {v}})=-\langle M({\mathbf {n}},{\mathbf {n}}),{\mathbf {v}}\rangle _{\mathrm {V}^*,\mathrm {V}}. \end{aligned}$$

In the above chain of equalities summation over repeated indexes is enforced. $\square$

#### Remark 2.3

1. For any ${\mathbf {f}}, {\mathbf {g}}\in {\mathbf {X}}_{1}$ and ${\mathbf {v}}\in \mathrm {H}$ we have
  2.11 $$\begin{aligned} \langle M({\mathbf {f}}, {\mathbf {g}}), {\mathbf {v}}\rangle _{\mathrm {V}^*, \mathrm {V}}= \langle \Pi [{{\,\mathrm{div}\,}}(\nabla {\mathbf {f}} \odot \nabla {\mathbf {g}})], {\mathbf {v}}\rangle . \end{aligned}$$
  In fact, for any ${\mathbf {f}}, {\mathbf {g}}\in {\mathbf {X}}_{1}$ and ${\mathbf {v}}\in {\mathcal {V}}$
  $$\begin{aligned} \langle M({\mathbf {f}},{\mathbf {g}}),{\mathbf {v}}\rangle _{\mathrm {V}^*,\mathrm {V}}&=-\langle \nabla {\mathbf {f}}\odot \nabla {\mathbf {g}}, \nabla {\mathbf {v}}\rangle \\&=\langle {{\,\mathrm{div}\,}}(\nabla {\mathbf {f}}\odot \nabla {\mathbf {g}}), \Pi {\mathbf {v}}\rangle \\&= \langle \Pi [{{\,\mathrm{div}\,}}(\nabla {\mathbf {f}}\odot \nabla {\mathbf {g}})], {\mathbf {v}}\rangle . \end{aligned}$$
  Thanks to the density of ${\mathcal {V}}$ in $\mathrm {H}$ we can easily show that the last line is still true for ${\mathbf {v}}\in \mathrm {H}$, which completes the proof of (2.11).
2. In some places in this manuscript we use the following shorthand notation:
  $$\begin{aligned} B({\mathbf {u}}):= B({\mathbf {u}},{\mathbf {u}}) \text { and } M({\mathbf {n}}):= M({\mathbf {n}},{\mathbf {n}}), \end{aligned}$$
  for any ${\mathbf {u}}$ and ${\mathbf {n}}$ such that the above quantities are meaningful.

We now fix the standing assumptions on the function $f(\cdot )$.

#### Assumption 2.1

Let $I_d$ be the set defined by

2.12 $$\begin{aligned} I_d={\left\{ \begin{array}{ll} {\mathbb {N}}:=\{1, 2, 3, \ldots \}&{}\quad {\text { if }} d=2,\\ \{1\},&{}\quad {\text { if }} d=3. \end{array}\right. } \end{aligned}$$

Throughout this paper we fix $N\in I_d$ and a family of numbers $a_k$, $k=0,\ldots , N$, with $a_N>0$. We define a function ${\tilde{f}}:[0,\infty ) \rightarrow {\mathbb {R}}$ by

$$\begin{aligned} {\tilde{f}}(r)=\sum _{k=0}^N a_k r^k, \text { for any } r\in {\mathbb {R}}_+. \end{aligned}$$

We define a mapping $f:{\mathbb {R}}^3\rightarrow {\mathbb {R}}^3$ by $f({\mathbf {n}})={\tilde{f}}(\vert {\mathbf {n}}\vert ^2){\mathbf {n}}$ where ${\tilde{f}}$ is as above.

We now assume that there exists $F: {\mathbb {R}}^3 \rightarrow {\mathbb {R}}$ a differentiable mapping such that for any ${\mathbf {n}}\in {\mathbb {R}}^3$ and ${\mathbf {g}}\in {\mathbb {R}}^3$

$$\begin{aligned} F^\prime ({\mathbf {n}})[{\mathbf {g}}]= f({\mathbf {n}})\cdot {\mathbf {g}} . \end{aligned}$$

Before proceeding further let us state few important remarks.

#### Remark 2.4

Let ${\tilde{F}}$ be an antiderivative of ${\tilde{f}}$ such that ${\tilde{F}}(0)=0$. Then, as a consequence of our assumption we have

$$\begin{aligned} {\tilde{F}}(r)=a_{N+1}r^{N+1}+U(r), \end{aligned}$$

where *U* is a polynomial function of at most degree *N* and $a_{N+1}>0$.

#### Remark 2.5

For any $r\in [0,\infty )$ let ${\tilde{f}}(r):=r-1$. If $1\in I_d$ then the mappings *f* and *F* defined on ${\mathbb {R}}^3$ by $f({\mathbf {n}}):={\tilde{f}}(\vert {\mathbf {n}}\vert ^2){\mathbf {n}}$ and $F({\mathbf {n}}):=\frac{1}{4} [{\tilde{f}}(\vert {\mathbf {n}}\vert ^2) ]^2$ for any ${\mathbf {n}}\in {\mathbb {R}}^3$ satisfy the above set of assumptions.

#### Remark 2.6

There exist two constants $\ell _1,\ell _2>0$ such that

2.13 $$\begin{aligned}&|{\tilde{f}}(r)|\le \ell _1 \left( 1+r^N\right) , \,\, r>0, \end{aligned}$$

2.14 $$\begin{aligned}&|{\tilde{f}}^\prime (r) |\le \ell _2 \left( 1+r^{N_1}\right) , \,\, r>0. \end{aligned}$$

#### Remark 2.7

Let *f* be defined as in Assumption 2.1.

(i) Then, there exist two positive constants $c>0$ and ${\tilde{c}}>0$ such that
  $$\begin{aligned} |f({\mathbf {n}})|\le c \left( 1+\vert {\mathbf {n}}\vert ^{2N+1}\right) \text { and } |f^{\prime }({\mathbf {n}})|\le {\tilde{c}}\left( 1+\vert {\mathbf {n}}\vert ^{2N}\right) \text { for any } {\mathbf {n}}\in {\mathbb {R}}^3. \end{aligned}$$
(ii) By performing elementary calculations we can check that there exists a constant $C>0$ such that for any ${\mathbf {n}}\in {\mathbf {H}}^2$
  2.15 $$\begin{aligned} \Vert \mathrm {A}_1{\mathbf {n}}\Vert ^2&=\Vert \mathrm {A}_1{\mathbf {n}}+f({\mathbf {n}})-f({\mathbf {n}})\Vert ^2 \le 2 \Vert \mathrm {A}_1{\mathbf {n}}+f({\mathbf {n}})\Vert ^2+2 \Vert f({\mathbf {n}})\Vert ^2,\nonumber \\&\le 2 \Vert \mathrm {A}_1{\mathbf {n}}+f({\mathbf {n}})\Vert ^2+C \Vert {\mathbf {n}}\Vert ^{{\tilde{q}}}_{{\mathbf {L}}^{{\tilde{q}}}}+C , \end{aligned}$$
  where $${\tilde{q}}=4N+2$$.
(iii) Observe also that since the norm $||\cdot ||_2$ is equivalent to $||\cdot ||+ ||\mathrm {A}_1\cdot ||$ on $D(\mathrm {A}_1)$, there exists a constant $C>0$ such that
  2.16 $$\begin{aligned} \Vert {\mathbf {n}}\Vert ^2_2\le C (\Vert \mathrm {A}_1{\mathbf {n}}+f({\mathbf {n}})\Vert ^2 +\Vert {\mathbf {n}}\Vert ^{{\tilde{q}}}_{{\mathbf {L}}^{{\tilde{q}}}}+1), \text{ for } \text{ any } {\mathbf {n}}\in D(\mathrm {A}_1). \end{aligned}$$
(iv) Finally, since ${\mathbf {H}}^1\subset {\mathbf {L}}^{4N+2}$ for any $N \in I_d$, we can use the previous observation to conclude that ${\mathbf {n}}\in {\mathbf {H}}^2\subset {\mathbf {L}}^\infty$ whenever ${\mathbf {n}}\in {\mathbf {H}}^1$ and $\mathrm {A}_1{\mathbf {n}}+f({\mathbf {n}})\in {\mathbf {L}}^2$.

### The assumption on the coefficients of the noise

Let $(\Omega , {\mathcal {F}}, {\mathbb {P}})$ be a complete probability space equipped with a filtration ${\mathbb {F}}=\{{\mathcal {F}}_t: t\ge 0\}$ satisfying the usual conditions, *i.e.* the filtration is right-continuous and all null sets of ${\mathcal {F}}$ are elements of ${\mathcal {F}}_0$. Let $W_2=(W_2(t))_{t\ge 0}$ be a standard ${\mathbb {R}}$-valued Wiener process on $(\Omega , {\mathcal {F}},{\mathbb {F}}, {\mathbb {P}})$. Let us also assume that $\mathrm {K}_1$ is a separable Hilbert space and $W_1=(W_1(t))_{t\ge 0}$ is a $\mathrm {K}_1$-cylindrical Wiener process on $(\Omega , {\mathcal {F}},{\mathbb {F}}, {\mathbb {P}})$. Throughout this paper we assume that $W_2$ and $W_1$ are independent. Thus we can assume that $W=(W_1(t),W_2(t))$ is a $\mathrm {K}$-cylindrical Wiener process on $(\Omega , {\mathcal {F}},{\mathbb {F}}, {\mathbb {P}})$, where

$$\begin{aligned} \mathrm {K}=\mathrm {K}_1\times {\mathbb {R}}. \end{aligned}$$

#### Remark 2.8

If $\mathrm {K}_2$ is a Hilbert space such that the embedding $\mathrm {K}_1\subset \mathrm {K}_2$ is Hilbert–Schmidt, then $W_1$ can be viewed as a $\mathrm {K}_2$-valued Wiener process. Moreover, there exists a trace class symmetric nonnegative operator $Q\in {\mathcal {L}}(\mathrm {K}_2)$ such that $W_1$ has covariance *Q*. This $\mathrm {K}_2$-valued $\mathrm {K}_1$-cylindrical Wiener process is characterised by, for all $t\ge 0$,

$$\begin{aligned} \mathbb {E} e^{i \; {\langle x^*, W(t)\rangle }_{\mathrm {K}_2^*,\mathrm {K}_2}}= e^{-\frac{t}{2} \vert x^*\vert _{\mathrm {K}_1}^2}, \;\; x^*\in \mathrm {K}_2^*, \end{aligned}$$

where $\mathrm {K}_2^*$ is the dual space to $\mathrm {K}_2$ such that identifying $\mathrm {K}_1^*$ with $\mathrm {K}_1$ we have

$$\begin{aligned} \mathrm {K}_2^*\hookrightarrow \mathrm {K}_1^*=\mathrm {K}_1 \hookrightarrow \mathrm {K}_2. \end{aligned}$$

Let $\tilde{\mathrm {H}}$ be a Hilbert space and ${\mathscr {M}}^2(\Omega \times [0,T]; {\mathcal {T}}_2({\mathrm {K}}, \tilde{\mathrm {H}} ))$ the space of all equivalence classes of ${\mathbb {F}}$-progressively measurable processes $\Psi : \Omega \times [0,T]\rightarrow {\mathcal {T}}_2({\mathrm {K}}, \tilde{\mathrm {H}} )$ satisfying

$$\begin{aligned} {\mathbb {E}} \int _0^T \Vert \Psi (s)\Vert ^2_{{\mathcal {T}}_2({\mathrm {K}}, \tilde{\mathrm {H}} )}ds <\infty . \end{aligned}$$

From the theory of stochastic integration on infinite dimensional Hilbert space, see [35, Chapter 5, Section 26 ] and [14, Chapter 4], for any $\Psi \in {\mathscr {M}}^2(\Omega \times [0,T]; {\mathcal {T}}_2({\mathrm {K}}, \tilde{\mathrm {H}} ))$ the process *M* defined by

$$\begin{aligned} M(t) =\int _0^t \Psi (s)dW(s), t\in [0,T], \end{aligned}$$

is a $\tilde{\mathrm {H}}$-valued martingale. Moreover, we have the following Itô isometry

2.17 $$\begin{aligned} {\mathbb {E}}^\prime \biggl (\biggl \Vert \int _0^t \Psi (s) dW(s)\biggr \Vert ^2_{\tilde{\mathrm {H}} }\biggr ) ={\mathbb {E}}^\prime \biggl (\int _0^t \Vert \Psi (s) \Vert ^2_{{\mathcal {T}}_2({\mathrm {K}}, \tilde{\mathrm {H}})} ds \biggr ), \forall t\in [0,T], \end{aligned}$$

and the Burkholder–Davis–Gundy inequality

2.18 $$\begin{aligned} {\mathbb {E}}^\prime \biggl (\sup _{0\le s\le t}\biggl \Vert \int _0^s \Psi (s) dW(s)\biggr \Vert ^q_{\tilde{\mathrm {H}} }\biggr )&\le C_q {\mathbb {E}}^\prime \biggl ( \int _0^t \Vert \Psi (s) \Vert ^2_{{\mathcal {T}}_2({\mathrm {K}},\tilde{\mathrm {H}})}ds \biggr )^\frac{q}{2}, \nonumber \\&\forall t\in [0,T], \forall q\in (1,\infty ). \end{aligned}$$

We also have the following relation between Stratonovich and Itô’s integrals, see [5],

$$\begin{aligned} G({\mathbf {n}})\circ dW_2= \frac{1}{2} G^2({\mathbf {n}}) \,dt + G({\mathbf {n}})\,dW_2, \end{aligned}$$

where $G^2=G\circ G$ is defined by

$$\begin{aligned} G^2({\mathbf {n}})&=G\circ G({\mathbf {n}})= ({\mathbf {n}}\times {\mathbf {h}})\times {\mathbf {h}}, \text { for any } {\mathbf {n}}\in {\mathbf {L}}^2. \end{aligned}$$

We now introduce the set of hypotheses that the function *S* must satisfy in this paper.

#### Assumption 2.2

We assume that $S: \mathrm {H}\rightarrow {\mathcal {T}}_2(\mathrm {K}_1,\mathrm {H})$ is a globally Lipschitz mapping. In particular, there exists $\ell _3\ge 0$ such that

2.19 $$\begin{aligned} \Vert S({\mathbf {u}})\Vert ^2_{{\mathcal {T}}_2}:=\Vert S({\mathbf {u}})\Vert ^2_{{\mathcal {T}}_2(\mathrm {K}_1,\mathrm {H})}\le \ell _3 (1+\Vert {\mathbf {u}}\Vert ^2),\;\; \text{ for } \text{ any } {\mathbf {u}}\in \mathrm {H}. \end{aligned}$$

## Existence and uniqueness of a weak martingale solution

In this section, we are going to establish the existence of a weak martingale solution to (1.13)–(1.17) which, using all the notations in the previous section, can be formally written in the following abstract form

3.1 $$\begin{aligned}&d{\mathbf {v}}(t)+\biggl (\mathrm {A}{\mathbf {v}}(t)+B({\mathbf {v}}(t), {\mathbf {v}}(t))+M({\mathbf {n}}(t))\biggr )dt=S({\mathbf {v}}(t))dW_1(t), \end{aligned}$$

3.2 $$\begin{aligned}&d{\mathbf {n}}(t)+\biggl (\mathrm {A}_1{\mathbf {n}}(t)+ {\tilde{B}}({\mathbf {v}}(t),{\mathbf {n}}(t))+ f({\mathbf {n}}(t))-\frac{1}{2} G^2({\mathbf {n}}(t))\biggr )dt=G({\mathbf {n}}(t))dW_2(t), \end{aligned}$$

3.3 $$\begin{aligned}&{\mathbf {v}}(0)={\mathbf {v}}_0 \text { and } {\mathbf {n}}(0)={\mathbf {n}}_0. \end{aligned}$$

For this purpose, we use the Galerkin approximation to reduce the original system to a system of finite-dimensional ordinary stochastic differential equations (SDEs for short). We establish several crucial uniform a priori estimates which will be used to prove the tightness of the family of laws of the sequence of solutions of the system of SDEs on appropriate topological spaces. However, before we proceed further, we define what we mean by weak martingale solution.

### Definition 3.1

Let $\mathrm {K}_1$ be as in Remark 2.8. By a weak martingale solution to (3.1)–(3.3) we mean a system consisting of a complete and filtered probability space

$$\begin{aligned} (\Omega ^\prime , {\mathcal {F}}^\prime , {\mathbb {F}}^\prime , {\mathbb {P}}^\prime ), \end{aligned}$$

with the filtration ${\mathbb {F}}^\prime =({\mathcal {F}}_t^\prime )_{t\in [0,T]}$ satisfying the usual conditions, and ${\mathbb {F}}^\prime$-adapted stochastic processes

$$\begin{aligned} ({\mathbf {v}}(t), {\mathbf {n}}(t), {\bar{W}}_{1}(t), {\bar{W}}_{2}(t))_{t\in [0,T]} \end{aligned}$$

such that:

1. $({\bar{W}}_{1}(t))_{t\in [0,T]}$ (resp. $({\bar{W}}_{2}(t))_{t\in [0,T]}$) is a $\mathrm {K}_1$-cylindrical (resp. real-valued) Wiener process,
2. $$({\mathbf {v}}, {\mathbf {n}}): [0,T]\times \Omega ^\prime \rightarrow \mathrm {V}\times {\mathbf {H}}^2$$ and $${\mathbb {P}}^\prime $$-a.e.
  3.4 $$\begin{aligned}&({\mathbf {v}}, {\mathbf {n}}) \in C([0,T]; \mathrm {V}_{-\beta })\times C([0,T];{\mathbf {X}}_{\beta }), \text { for any } \beta \in \left( 0,\frac{1}{2}\right) , \end{aligned}$$
  3.5 $$\begin{aligned}&{\mathbb {E}}^\prime \sup _{0\le s\le T}\left[ \Vert {\mathbf {v}}(s)\Vert +\Vert \nabla {\mathbf {n}}(s)\Vert \right] + {\mathbb {E}}^\prime \int _0^T \left( \Vert \nabla {\mathbf {v}}(s)\Vert ^2+ \Vert \mathrm {A}_1{\mathbf {n}}(s)\Vert ^2\right) ds<\infty , \end{aligned}$$
3. for each $$(\Phi , \Psi )\in \mathrm {V}\times {\mathbf {L}}^2$$ we have for all $$t\in [0,T]$$$${\mathbb {P}}^\prime $$-a.s..
  3.6 $$\begin{aligned}&\langle {\mathbf {v}}(t)-{\mathbf {v}}_0, \Phi \rangle +\int _0^t \biggl \langle \mathrm {A}{\mathbf {v}}(s)+B({\mathbf {v}}(s), {\mathbf {v}}(s))+M({\mathbf {n}}(s)), \Phi \biggr \rangle _{\mathrm {V}^*,\mathrm {V}} ds\nonumber \\&\quad = \int _0^t \langle \Phi , S({\mathbf {v}}(s))d{\bar{W}}_1(s)\rangle , \end{aligned}$$
  and
  3.7 $$\begin{aligned}&\langle {\mathbf {n}}(t)-{\mathbf {n}}_0,\Psi \rangle +\int _0^t \biggl \langle \mathrm {A}_1{\mathbf {n}}(s)+ {\tilde{B}}({\mathbf {v}}(s),{\mathbf {n}}(s))+f({\mathbf {n}}(s))-\frac{1}{2} G^2({\mathbf {n}}(s)), \Psi \biggr \rangle ds\nonumber \\&\quad =\int _0^t \langle G({\mathbf {n}}(s)), \Psi \rangle d{\bar{W}}_2(s). \end{aligned}$$

Now we can state our first result in the following theorem.

### Theorem 3.2

If Assumptions 2.2 and 2.1 are satisfied, ${\mathbf {h}}\in {\mathbf {W}}^{1,3}\cap {\mathbf {L}}^\infty$, ${\mathbf {v}}_0\in \mathrm {H}$, ${\mathbf {n}}_0\in {\mathbf {H}}^1$, and $d=2,3$, then the system (3.1)–(3.3) has a weak martingale solution in the sense of Definition 3.1.

### Proof

The proof will be carried out in Sects. 3.1–3.3. $\square$

Before we state the uniqueness of the weak martingale solution we should make the following remark.

### Remark 3.3

We should note that the existence of weak martingale solutions stated in Theorem 3.2 still holds if we assume that the mapping $S(\cdot )$ is only continuous and satisfies a linear growth condition of the form (2.19).

To close this subsection we assume that $d=2$ and we state the following uniqueness result.

### Theorem 3.4

Let $d=2$ and assume that $({\mathbf {v}}_i,{\mathbf {n}}_i)$,   $i=1,2$ are two solutions of (3.1) and (3.3) defined on the same stochastic system $(\Omega , {\mathcal {F}}, {\mathbb {F}},{\mathbb {P}},W_1, W_2)$ and with the same initial condition $({\mathbf {v}}_0,{\mathbf {n}}_0)\in \mathrm {H}\times {\mathbf {H}}^1$, then for any $t\in (0,T]$ we have ${\mathbb {P}}$-a.s.

$$\begin{aligned} ({\mathbf {v}}_1(t),{\mathbf {n}}_1(t))=({\mathbf {v}}_2(t),{\mathbf {n}}_2(t) ). \end{aligned}$$

### Remark 3.5

Due to the continuity given in (3.4) the two solutions are indistinguishable. Therefore, uniqueness holds.

### Proof

The proof of this result will be carried out in Sect. 3.4. $\square$

### Galerkin approximation and a priori uniform estimates

As we mentioned earlier, the proof of the existence of weak martingale solution relies on the Galerkin and compactness methods. This subsection will be devoted to the construction of the approximating solutions and the proofs of crucial estimates satisfied by these solutions.

Recall that there exists an orthonormal basis $(\varphi _i)_{i=1}^n\subset {\mathcal {C}}^\infty$ of $\mathrm {H}$ consisting of the eigenvectors of the Stokes operator $\mathrm {A}$. Recall also that there exists an orthonormal basis $(\phi _i)_{i=1}^\infty \subset {\mathcal {C}}^\infty$ of ${\mathbf {L}}^2$ consisting of the eigenvectors of the Neumann Laplacian $\mathrm {A}_1$. For any $m\in {\mathbb {N}}$ let us define the following finite-dimensional spaces

$$\begin{aligned}&\mathrm {H}_m:={{\,\mathrm{linspan}\,}}\{\varphi _1, \ldots , \varphi _m\},\\&{\mathbf {L}}_m:={{\,\mathrm{linspan}\,}}\{\phi _1, \ldots , \phi _m\}. \end{aligned}$$

In this subsection, we introduce the finite-dimensional approximation of the system (3.1)–(3.3) and justify the existence of solution of such approximation. We also derive uniform estimates for the sequence of approximating solutions. To do so, denote by $\pi _m$ (resp. ${\hat{\pi }}_m$) the projection from $\mathrm {H}$ (resp. ${\mathbf {L}}^2$) onto $\mathrm {H}_m$ (resp. ${\mathbf {L}}_m$). These operators are self-adjoint, and their operator norms are equal to 1. Remark 6.3, Lemma 6.2 enable us to define the following mappings

$$\begin{aligned}&{B}_{m}: \mathrm {H}_m \ni {\mathbf {u}}\mapsto \pi _mB({\mathbf {u}},{\mathbf {u}}) \in \mathrm {H}_m,\\&{\tilde{B}}_{m}:\mathrm {H}_m\times {\mathbf {L}}_m \ni ({\mathbf {u}},{\mathbf {n}})\mapsto {\hat{\pi }}_m{\tilde{B}}({\mathbf {v}},{\mathbf {n}}) \in {\mathbf {L}}_m,\\&M_m: {\mathbf {L}}_m \ni {\mathbf {n}}\mapsto \pi _mM({\mathbf {n}}) \in \mathrm {H}_m,\\ \end{aligned}$$

From the definition of $${\mathbf {L}}_m$$ and the regularity of elements of the basis $$(\phi )_{i=1}^\infty $$ we infer that for any $${\mathbf {u}}\in {\mathbf {L}}_m$$$$|{\mathbf {u}}|^{2r}{\mathbf {u}}\in {\mathbf {L}}^2$$ for any $$r\in \{1,\ldots ,N\}$$. Hence the mapping $$f_m$$ defined by

$$\begin{aligned} f_m: {\mathbf {L}}_m \ni {\mathbf {n}}\mapsto {\hat{\pi }}_mf({\mathbf {n}}) \in {\mathbf {L}}_m, \end{aligned}$$

is well-defined. From the assumptions on *S* and ${\mathbf {h}}$ the following mappings are well-defined,

$$\begin{aligned}&S_m: \mathrm {H}_m \ni {\mathbf {u}}\mapsto \pi _m\circ S({\mathbf {u}})\in {\mathcal {T}}_2(\mathrm {K}_1, \mathrm {H}_m),\\&G_m: {\mathbf {L}}_m \ni {\mathbf {n}}\mapsto {\hat{\pi }}_mG({\mathbf {n}})\in {\mathbf {L}}_m,\\&G^2_m:{\mathbf {L}}_m \ni {\mathbf {n}}\mapsto {\hat{\pi }}_mG^2({\mathbf {n}}) \in {\mathbf {L}}_m. \end{aligned}$$

#### Lemma 3.6

For each m let $\Psi _m$ an $\Phi _m$ be two mappings on $\mathrm {H}_m \times {\mathbf {L}}_m$ defined by

$$\begin{aligned} \Psi _m({\mathbf {u}},{\mathbf {n}})=\begin{pmatrix} \mathrm {A}{\mathbf {u}}+ B_m({\mathbf {u}})+ M_m({\mathbf {n}})\\ \mathrm {A}_1{\mathbf {n}}+{\tilde{B}}_m({\mathbf {u}},{\mathbf {n}}) + f_m({\mathbf {n}})-\frac{1}{2} G^2_m({\mathbf {n}}) \end{pmatrix},\;\; ({\mathbf {u}}, {\mathbf {n}})\in \mathrm {H}_m \times {\mathbf {L}}_m, \end{aligned}$$

and

$$\begin{aligned} \Phi _m({\mathbf {u}},{\mathbf {n}})=\begin{pmatrix} S_m({\mathbf {u}}) &{} 0\\ 0 &{} G_m({\mathbf {n}}) \end{pmatrix}, ({\mathbf {u}}, {\mathbf {n}})\in \mathrm {H}_m \times {\mathbf {L}}_m. \end{aligned}$$

Then, the mappings $\Psi _m$ and $\Phi _m$ are locally Lipschitz.

#### Proof

The mapping $S_m$ is globally Lipschitz as the composition of a continuous linear operator and a globally Lipschitz mapping. Since $\mathrm {A}$, $\mathrm {A}_1$, $G_m$ and $G^2_m$ are linear, they are globally Lipschitz. Thus, $\Phi$ is also globally Lipschitz.

From the bilinearity of $B(\cdot ,\cdot )$, the boundedness of $\pi _m$ and Remark 6.3 we infer that there exists a constant $C>0$, depending on *m*, such that for any ${\mathbf {u}}, {\mathbf {v}}\in \mathrm {H}_m$

3.8 $$\begin{aligned} ||B_m({\mathbf {u}},{\mathbf {u}})-B_m({\mathbf {v}},{\mathbf {v}}) ||\le C[||{\mathbf {u}}-{\mathbf {v}}||_1 ||{\mathbf {v}}||_2 + ||{\mathbf {u}}||_1 ||{\mathbf {u}}-{\mathbf {v}}||_2]. \end{aligned}$$

Since the ${\mathbb {L}}^2$, ${\mathbb {H}}^1$ and ${\mathbb {H}}^2$ norms are equivalent on the finite dimensional space $\mathrm {H}_m$ we infer that for any $m \in {\mathbb {N}}$ there exists a constant $C>0$, depending on *m*, such that

3.9 $$\begin{aligned} ||B_m({\mathbf {u}},{\mathbf {u}})-B_m({\mathbf {v}},{\mathbf {v}}) ||\le C[||{\mathbf {u}}-{\mathbf {v}}||||{\mathbf {v}}||+ ||{\mathbf {u}}||||{\mathbf {u}}-{\mathbf {v}}||], \end{aligned}$$

from which we infer that for any number $R>0$ there exists a constant $C_R>0$, also depending on *m*, such that

$$\begin{aligned} ||B_m({\mathbf {u}},{\mathbf {u}})-B_m({\mathbf {v}},{\mathbf {v}}) ||\le C_R ||{\mathbf {u}}-{\mathbf {v}}||, \end{aligned}$$

for any ${\mathbf {u}},{\mathbf {v}}\in \mathrm {H}_m$ with $\Vert {\mathbf {u}}||,\Vert {\mathbf {v}}\Vert \le R$. That is, $B_m(\cdot ):=B_m(\cdot ,\cdot )$ is locally Lipschitz. Thanks to (6.11) one can also use the same idea to show that $M_m$ is locally Lipschitz with Lipschitz constant depending on *m*. Now, for any $r\in \{1,\ldots ,N\}$ there exists a constant $C>0$ such that for any ${\mathbf {n}}_1,{\mathbf {n}}_2\in {\mathbf {L}}_m$

$$\begin{aligned}&|||{\mathbf {n}}_1|^{2r}{\mathbf {n}}_1-|{\mathbf {n}}_2|^{2r}{\mathbf {n}}_2||\le C \Vert \vert {\mathbf {n}}_1\vert ^{2r}\Vert _{{\mathbf {L}}^\infty }||{\mathbf {n}}_1-{\mathbf {n}}_2||\\&\quad +C ||{\mathbf {n}}_1-{\mathbf {n}}_2||||{\mathbf {n}}_2\left( \sum _{k=0}^{2r-1}\vert {\mathbf {n}}_1\vert ^{2r-1-k}\vert {\mathbf {n}}_2|^{k}\right) ||_{{\mathbf {L}}^\infty }, \end{aligned}$$

from which we easily derive the local Lipschitz property of $f_m$.

Finally, thanks to (6.9) there exists a constant $C>0$, which depends on $m\in {\mathbb {N}}$, such that

$$\begin{aligned} ||{\tilde{B}}_m({\mathbf {u}}_1,{\mathbf {n}}_1)-{\tilde{B}}_m ({\mathbf {u}}_2, {\mathbf {n}}_2)||\le C \left[ ||{\mathbf {u}}_1- {\mathbf {u}}_2 ||||{\mathbf {n}}_2||+ ||{\mathbf {u}}_2||||{\mathbf {n}}_1 -{\mathbf {n}}_2 ||\right] , \end{aligned}$$

where we have used the equivalence of all norms on the finite dimensional space $\mathrm {H}_m\times {\mathbf {L}}_m$ again. Now, it is clear that the mapping $\Psi$ is locally Lipschitz. $\square$

Let ${\mathbf {n}}_{0m}={\hat{\pi }}_m{\mathbf {n}}_0$ and ${\mathbf {v}}_{0m}=\pi _m{\mathbf {v}}_0$. The Galerkin approximation to (3.1)–(3.3) is

3.10 $$\begin{aligned}&d{\mathbf {v}}_m(t)+\big [\mathrm {A}{\mathbf {v}}_m(t)+{B}_{m}({\mathbf {v}}_m(t))+M_m({\mathbf {n}}_m(t))\big ]dt=S_m({\mathbf {v}}_m(t))dW_1(t), \qquad \quad \end{aligned}$$

3.11 $$\begin{aligned}&d{\mathbf {n}}_m(t)+\big [\mathrm {A}_1{\mathbf {n}}_m(t)+{\tilde{B}}_{m}({\mathbf {v}}_m(t),{\mathbf {n}}_m(t))+f_m({\mathbf {n}}_m(t))\big ]dt\nonumber \\&\quad =\frac{1}{2} G^2_m({\mathbf {n}}_m(t))+G_m({\mathbf {n}}_m(t))dW_2(t). \end{aligned}$$

The Eqs. (3.10)–(3.11) with initial condition ${\mathbf {v}}_m(0)={\mathbf {v}}_{0m} \text { and } {\mathbf {n}}_m(0)={\mathbf {n}}_{0m}$ form a system of stochastic ordinary differential equations which can be rewritten as

3.12 $$\begin{aligned} d{\mathbf {y}}_m + \Psi _m({\mathbf {y}}_m) dt = \Phi _m({\mathbf {y}}_m) dW,\quad {\mathbf {y}}_m(0)=({\mathbf {v}}_{0m}, {\mathbf {n}}_{0m}) \end{aligned}$$

where ${\mathbf {y}}_m:=({\mathbf {u}}_m,{\mathbf {n}}_m)$, $W:=(W_1, W_2)$. Due to Lemma 3.6 the mappings $\Psi _m$ and $\Phi _m$ are locally Lipschitz. Hence, owing to [1, 38, Theorem 38, p. 303] it has a unique local maximal solution $({\mathbf {v}}_m,{\mathbf {n}}_m; T_m)$ where $T_m$ is a stopping time.

#### Remark 3.7

In case we assume that $S(\cdot )$ is only continuous and satisfies (2.19), $S_m$ is only continuous and locally bounded. However, with this assumption, we can still justify the existence, possibly non-unique, of a weak local martingale solution to (3.10)–(3.11) by using results in [26, Chapter IV, Section 2, pp 167–177].

We now derive uniform estimates for the approximating solutions. For this purpose, let $\tau _{R,m}$, $m,R\in {\mathbb {N}}$, be a stopping time defined by

3.13 $$\begin{aligned} \tau _{R,m}= \inf \{t\in [0,T]; ||{\mathbf {n}}_m(t)||_1^2 + ||{\mathbf {v}}_m(t)||^2 \ge R^2 \}\wedge T. \end{aligned}$$

#### Proposition 3.8

If all the assumptions of Theorem 3.2 are satisfied, then for any $p\ge 2$ there exists a positive constant $C_p$ such that we have for all $R>0$ and $t \in (0,T]$

3.14 $$\begin{aligned}&\sup _{m\in {\mathbb {N}}}\biggl ({\mathbb {E}} \sup _{s\in [0, t\wedge \tau _{R,m}]}\Vert {\mathbf {n}}_m(s)\Vert ^p+p \int _0^{t\wedge \tau _{R,m}} \Vert {\mathbf {n}}_m(s) \Vert ^{p-2} \Vert \nabla {\mathbf {n}}_m(s)\Vert ^2 ds\nonumber \\&\quad + \,p \int _0^{t\wedge \tau _{R,m}} \Vert {\mathbf {n}}_m(s) \Vert ^{p-2} \Vert {\mathbf {n}}_m(s)\Vert ^{2N+2}_{{\mathbf {L}}^{2N+2}} ds\biggr )\le {\mathbb {E}}{\mathfrak {G}}_0(T,p), \end{aligned}$$

where

3.15 $$\begin{aligned} {\mathfrak {G}}_0(T,p):=\Vert {\mathbf {n}}_0\Vert ^p (C_p+C_p e^{C_p T}). \end{aligned}$$

#### Proof

The proof will be given in Sect. 4. $\square$

We also have the following estimates.

#### Proposition 3.9

If all the assumptions of Theorem 3.2 are satisfied, then there exists ${\tilde{\ell }}>0$ such that for all $p \in [1,\infty )$, for all $R>0$ and $t\in (0,T]$

3.16 $$\begin{aligned}&\sup _{m \in {\mathbb {N}}}{\mathbb {E}} \biggl [\sup _{0\le s\le t\wedge \tau _{R,m}}\left( \Vert {\mathbf {v}}_m(s)\Vert ^{2}+{\tilde{\ell }}\Vert {\mathbf {n}}_m(s)\Vert ^2 +\Vert \nabla {\mathbf {n}}_m(s)\Vert ^{2}+ \int _{\mathcal {O}}F({\mathbf {n}}_m(s,x)) dx \right) ^p\nonumber \\&\quad \qquad + \left( \int _0^{t\wedge \tau _{R,m}} \left( \Vert \nabla {\mathbf {v}}_m(s)\Vert ^2+ \Vert \mathrm {A}_1{\mathbf {n}}_m(s)+f({\mathbf {n}}_m(s))\Vert ^2\right) \right) ^p\biggr ] \nonumber \\&\qquad \le {\mathfrak {G}}_1(T, p), \quad t\in [0,T],\,m\in {\mathbb {N}}, \end{aligned}$$

and

3.17 $$\begin{aligned}&\sup _{m \in {\mathbb {N}}} {\mathbb {E}}\biggl [\int _0^{t\wedge \tau _{R,m}} \Vert \mathrm {A}_1{\mathbf {n}}_m(s)\Vert ^2 ds\biggr ]^p \le {\mathfrak {G}}_1(T, p\cdot (2N+1)),\nonumber \\&\quad t\in [0,T],\,m\in {\mathbb {N}}, \end{aligned}$$

where

3.18 $$\begin{aligned} {\mathfrak {G}}_1(T, p):= & {} \biggl [\left( \Vert {\mathbf {v}}_0\Vert ^2 +\Vert {\mathbf {n}}_0\Vert ^2+\Vert \nabla {\mathbf {n}}_0\Vert ^2+\int _{\mathcal {O}}F({\mathbf {n}}_0(x)) dx\right) ^p+ \,\kappa T+\kappa {\mathfrak {G}}_0(T,p)\biggr ] \nonumber \\&\quad \times \left[ 1+ \kappa T(T+1) e^{\kappa (T+1)T }\right] . \end{aligned}$$

Here, $\kappa >0$ is a constant which depends only on *p* and ${\tilde{\ell }}$, and ${\mathfrak {G}}_0$ is defined in (3.15).

#### Proof

The proof of (3.16) will be given in Sect. 4.

The estimate (3.17) easily follows from (3.16), (3.14) and item (ii) of Remark 2.7 (see also item (iii) of the same remark). $\square$

In the next step we will take the limit $R\rightarrow \infty$ in the above estimates, but before proceeding further, we state and prove the following lemma.

#### Lemma 3.10

Let $$\tau _{R,m}$$, $$R, m \in {\mathbb {N}}$$ be the stopping times defined in (3.13). Then we have for any $$m \in {\mathbb {N}}$$$${\mathbb {P}}$$–a.s.

$$\begin{aligned} \lim _{R\rightarrow \infty }\tau _{R,m} =T . \end{aligned}$$

#### Proof

Since $({\mathbf {v}}_m,{\mathbf {n}}_m)(\cdot \wedge \tau _{R,m}):[0,T]\rightarrow \mathrm {H}_m\times {\mathbf {L}}_m$ is continuous we have

$$\begin{aligned} R^2{\mathbb {P}} (\tau _{R,m}<t )&\le {\mathbb {E}}\left[ 1_{\tau _{R,m}<t}(||{\mathbf {v}}_m(\tau _{R,m})||^2+||{\mathbf {n}}_m(\tau _{R,m}) ||_1^2)\right] \\&\le {\mathbb {E}}\left[ 1_{\tau _{R,m}<t}(||{\mathbf {v}}_m(\tau _{R,m})||^2+||{\mathbf {n}}_m(\tau _{R,m}) ||_1^2)\right] \\&\quad + {\mathbb {E}}\left[ 1_{\tau _{R,m}\ge t}(||{\mathbf {v}}_m(\tau _{R,m})||^2+||{\mathbf {n}}_m(\tau _{R,m}) ||_1^2)\right] \\&= {\mathbb {E}}\left[ ||{\mathbf {v}}_m(\tau _{R,m})||^2+||{\mathbf {n}}_m(\tau _{R,m}) ||_1^2\right] , \end{aligned}$$

for any $m \in {\mathbb {N}}$ and $t\in [0,T]$. From the last line of the above chain of inequalities and Proposition 3.9 we infer that

3.19 $$\begin{aligned} {\mathbb {P}}(\tau _{R,m}<t)\le \frac{1}{R^2} {\mathfrak {S}}_1(T,2). \end{aligned}$$

Hence

$$\begin{aligned} \lim _{R\rightarrow \infty }{\mathbb {P}}(\tau _{R,m}<t)=0 \hbox { for all } t\in [0,T] \hbox { and } m \in {\mathbb {N}}, \end{aligned}$$

which implies that there exists a subsequence $\tau _{R_k,m}$ such that $\tau _{R_k,m}\rightarrow T$ a.s., which along with the fact that $(\tau _{R,m})_{R\in {\mathbb {N}}}$ is increasing, yields that $\tau _{R,m}\nearrow T$ a.s. for any $m \in {\mathbb {N}}$. This completes the proof of the lemma. $\square$

We now state the following corollary.

#### Corollary 3.11

If all the assumptions of Theorem 3.2 are satisfied, then we have

3.20 $$\begin{aligned}&\sup _{m\in {\mathbb {N}}}\biggl ({\mathbb {E}} \sup _{s\in [0, T]}\Vert {\mathbf {n}}_m(s)\Vert ^p+p \int _0^{T} \Vert {\mathbf {n}}_m(s) \Vert ^{p-2} \Vert \nabla {\mathbf {n}}_m(s)\Vert ^2 ds\nonumber \\&\quad + \,p \int _0^{T} \Vert {\mathbf {n}}_m(s) \Vert ^{p-2} \Vert {\mathbf {n}}_m(s)\Vert ^{2N+2}_{{\mathbf {L}}^{2N+2}} ds\biggr )\le {\mathbb {E}}{\mathfrak {G}}_0(T,p). \end{aligned}$$

Furthermore, there exists ${\tilde{\ell }}>0$ such that for all $p \in [1,\infty )$

3.21 $$\begin{aligned}&\sup _{m \in {\mathbb {N}}}{\mathbb {E}} \biggl [\sup _{0\le s\le T}\left( \Vert {\mathbf {v}}_m(s)\Vert ^{2}+{\tilde{\ell }}\Vert {\mathbf {n}}_m(s)\Vert ^2 +\Vert \nabla {\mathbf {n}}_m(s)\Vert ^{2}+ \int _{\mathcal {O}}F({\mathbf {n}}_m(s,x)) dx \right) ^p\nonumber \\&\quad \qquad + \left( \int _0^{T} \left( \Vert \nabla {\mathbf {v}}_m(s)\Vert ^2+ \Vert \mathrm {A}_1{\mathbf {n}}_m(s)+f({\mathbf {n}}_m(s))\Vert ^2\right) \right) ^p\biggr ] \nonumber \\&\qquad \le {\mathfrak {G}}_1(T, p), \quad t\in [0,T],\,m\in {\mathbb {N}}, \end{aligned}$$

and

3.22 $$\begin{aligned} \sup _{m \in {\mathbb {N}}} {\mathbb {E}}\biggl [\int _0^{T} \Vert \mathrm {A}_1{\mathbf {n}}_m(s)\Vert ^2 ds\biggr ]^p \le {\mathfrak {G}}_1(T, p\cdot (2N+1)), \quad t\in [0,T],\,m\in {\mathbb {N}}.\qquad \quad \end{aligned}$$

The quantities ${\mathfrak {G}}_0$ and ${\mathfrak {G}}_1$ are defined in (3.15) and (3.18), respectively.

#### Proof

Thanks to Lemma  3.10 the inequalities (3.20), (3.21) and (3.22) can be established by using Fatou’s lemma and passing to the limit (as $R\rightarrow \infty$) in (3.14), (3.16) and (3.17). $\square$

In the next proposition, we prove two uniform estimates for ${\mathbf {v}}_m$ and ${\mathbf {n}}_m$ which are very crucial for our purpose.

#### Proposition 3.12

In addition to the assumptions of Theorem  3.2, let $\alpha \in (0,\frac{1}{2})$ and $p\in [2,\infty )$ such that $1-\frac{d}{4}\ge \alpha -\frac{1}{p}$. Then, there exist positive constants $\bar{\kappa }_5$ and $\bar{\kappa }_6$ such that we have

3.23 $$\begin{aligned} \sup _{m\in {\mathbb {N}}} {\mathbb {E}}\Vert {\mathbf {v}}_m\Vert ^2_{W^{\alpha ,p}(0,T; \mathrm {V}^*)}\le \bar{\kappa }_5, \end{aligned}$$

and

3.24 $$\begin{aligned} \sup _{m\in {\mathbb {N}}}{\mathbb {E}} \Vert {\mathbf {n}}_m\Vert ^2_{W^{\alpha ,p}(0,T;{\mathbf {L}}^{2})}\le \bar{\kappa }_6. \end{aligned}$$

#### Proof

We rewrite the equation for ${\mathbf {v}}_m$ as

$$\begin{aligned} \begin{aligned} {\mathbf {v}}_m(t)&={\mathbf {v}}_{0m}-\int _0^t \mathrm {A}{\mathbf {v}}_m(s)ds -\int _0^t {B}_{m}({\mathbf {v}}_m(s),{\mathbf {v}}_m(s))ds -\int _0^t M_m({\mathbf {n}}_m(s)) ds\\&\quad +\int _0^t S_m({\mathbf {v}}_m(s))dW_1(s),\\&= {\mathbf {v}}_{0m}+\sum _{i=1}^4 I_m^i(t). \end{aligned} \end{aligned}$$

Since $\mathrm {A}\in {\mathcal {L}}(\mathrm {V},\mathrm {V}^*)$, we infer from (3.21) along with Corollary 3.11 that there exists a certain constant $C>0$ such that

3.25 $$\begin{aligned} \sup _{m\in {\mathbb {N}}}{\mathbb {E}} \Vert I^1_m\Vert _{W^{1,2}(0,T; \mathrm {V}^*)}^2= & {} \sup _{m \in {\mathbb {N}}}{\mathbb {E}}\biggl \Vert \int _0^\cdot \mathrm {A}{\mathbf {v}}_m(s)ds\biggr \Vert _{W^{1,2}(0,T; \mathrm {V}^*)}^2\nonumber \\&\le C,\quad m\in {\mathbb {N}}. \end{aligned}$$

Applying [18, Lemma 2.1] and (2.19) in Assumption 2.2 we infer that there exists a constant $c>0$ such that that for any $\alpha \in (0,\frac{1}{2})$ and $p\in [2,\infty )$

$$\begin{aligned} \begin{aligned} \sup _{m \in {\mathbb {N}}} {\mathbb {E}}\Vert I^4_m\Vert ^p_{W^{\alpha , p}(0,T;\mathrm {H})}&= \sup _{m \in {\mathbb {N}}} {\mathbb {E}} \biggl \Vert \int _0^\cdot S_m({\mathbf {v}}_m(s))dW_1(s) \biggr \Vert ^p_{W^{\alpha , p}(0,T;\mathrm {H})}\\&\le c {\mathbb {E}}\int _0^T \Vert S_m({\mathbf {v}}_m(t))\Vert _{{\mathcal {T}}_2(\mathrm {K}_1; \mathrm {H})}^p dt,\\&\le c \ell _3^p {\mathbb {E}}\int _0^T(1+ \Vert {\mathbf {v}}_m(t)\Vert ^p)ds. \end{aligned} \end{aligned}$$

Now, invoking (3.21) and Corollary 3.11 we derive that there exists a constant $C>0$ such that

3.26 $$\begin{aligned} \sup _{m\in {\mathbb {N}}} {\mathbb {E}}\Vert I^4_m\Vert ^p_{W^{\alpha , p}(0,T;\mathrm {H})}\le C. \end{aligned}$$

Now, we treat the term $I^3_m(t)$. From (2.9) we infer that there exists a constant $C>0$ such that for any $m \in {\mathbb {N}}$

$$\begin{aligned} \Vert M_m({\mathbf {n}}_m)\Vert ^2_{L^{\frac{4}{d}}(0,T; \mathrm {V}^*) }&\le C \left( \int _0^T ||\nabla {\mathbf {n}}_m(t)||^{\frac{2(4-d)}{d}} ||\nabla ^2 {\mathbf {n}}_m(t)||^2 dt \right) ^\frac{d}{2}\\&\le C \sup _{t\in [0,T]}||\nabla {\mathbf {n}}_m(t)||^{4-d}\left( \int _0^T ||\nabla ^2 {\mathbf {n}}_m(t)||^2 dt \right) ^\frac{d}{2}. \end{aligned}$$

Hence, there exists a constant $C>0$ such that

$$\begin{aligned} \sup _{m \in {\mathbb {N}}}{\mathbb {E}} \Vert M_m({\mathbf {n}}_m)\Vert ^2_{L^{\frac{4}{d}}(0,T; \mathrm {V}^*) }\le C \biggl [{\mathbb {E}} \bigg (\sup _{0\le t\le T}\Vert \nabla {\mathbf {n}}_m(t)\Vert ^{2(4-d)} \bigg ) {\mathbb {E}}\biggl (\int _0^T \Vert {\mathbf {n}}_m(t)\Vert ^2_2 dt \biggr )^d \biggr ]^\frac{1}{2}, \end{aligned}$$

from which altogether with (3.21), (3.22) and Corollary 3.11 we infer that there exists a constant $C>0$ such that

3.27 $$\begin{aligned} \sup _{m \in {\mathbb {N}}}{\mathbb {E}} \Vert I^3_m\Vert ^2_{W^{1,\frac{4}{d}}(0,T; \mathrm {V}^*)}=\sup _{m \in {\mathbb {N}}}{\mathbb {E}}\biggl \Vert \int _0^t M_m({\mathbf {n}}_m(s)) ds\biggr \Vert ^2_{W^{1,\frac{4}{d}}(0,T; \mathrm {V}^*)} \le C.\qquad \end{aligned}$$

Using (6.8) and an argument similar to the proof of the estimate for $I_m^3$ we conclude that there exists a constant $C>0$ such that

$$\begin{aligned}&\sup _{m \in {\mathbb {N}}}{\mathbb {E}}\biggl \Vert \int _0^\cdot {B}_{m}({\mathbf {v}}_m(s),{\mathbf {v}}_m(s))ds\biggr \Vert ^2_{W^{1,\frac{4}{d}}(0,T; \mathrm {V}^*)} \\&\quad \le C \biggl [{\mathbb {E}}\bigg ( \sup _{0\le t\le T}\Vert {\mathbf {v}}_m(t)\Vert ^{2(4-d)} \bigg ){\mathbb {E}}\biggl (\int _0^T \Vert {\mathbf {v}}_m(t)\Vert ^2_2 dt \biggr )^d \biggr ]^\frac{1}{2}, \end{aligned}$$

from which along with (3.21) and Corollary 3.11 we conclude that there exists a constant $C>0$ such that

3.28 $$\begin{aligned} \sup _{m\in {\mathbb {N}}}{\mathbb {E}} \Vert I^2_m\Vert ^2_{W^{1,\frac{4}{d}}(0,T; \mathrm {V}^*) }&=\sup _{m \in {\mathbb {N}}}{\mathbb {E}}\biggl \Vert \int _0^\cdot {B}_{m}({\mathbf {v}}_m(s),{\mathbf {v}}_m(s))ds\biggr \Vert ^2_{W^{1,\frac{4}{d}}(0,T; \mathrm {V}^*) }<C. \end{aligned}$$

By [44, Section 11, Corollary 19] we have the continuous imbedding

3.29 $$\begin{aligned} W^{1,\frac{4}{d}}(0,T;\mathrm {V}^*) \subset W^{\alpha , p}(0,T; \mathrm {V}^*), \end{aligned}$$

for $\alpha \in (0,\frac{1}{2})$ and $p\in [2,\infty )$ such that $1-\frac{d}{4}\ge \alpha -\frac{1}{p}$. Owing to Eqs. (3.25), (3.27), (3.26) and (3.28) and this continuous embedding we infer that (3.23) holds.

The second equations for the Galerkin approximation is written as

$$\begin{aligned} \begin{aligned} {\mathbf {n}}_m(t)&={\mathbf {n}}_{0m}-\int _0^t \mathrm {A}_1{\mathbf {n}}_m(s) ds -\int _0^t{\hat{\pi }}_m[ {\tilde{B}}_{m}({\mathbf {v}}_m(s),{\mathbf {n}}_m(s))] ds -\int _0^t f_m({\mathbf {n}}_m(s)) ds\\&\quad +\frac{1}{2} \int _0^t G^2_m({\mathbf {n}}_m(s)) ds +\int _0^t G_m({\mathbf {n}}_m(s)) dW_2(s),\\&=: {\mathbf {n}}_{0m}+\sum _{j=1}^5 J^j_m(t). \end{aligned} \end{aligned}$$

From (3.22) and Corollary 3.11 we clearly see that

3.30 $$\begin{aligned} \sup _{m\in {\mathbb {N}}}{\mathbb {E}} \Vert J^1_m\Vert ^2_{W^{1,2}(0,T;{\mathbf {L}}^2)}= \sup _{m \in {\mathbb {N}}}{\mathbb {E}} \biggl \Vert \int _0^\cdot \mathrm {A}_1{\mathbf {n}}_m(s) ds\biggr \Vert ^2_{W^{1,2}(0,T;{\mathbf {L}}^2)}\le C. \end{aligned}$$

From (6.9) we infer that there exists a constant $c>0$ such that

$$\begin{aligned} \Vert {\hat{\pi }}_m[ {\tilde{B}}_{m}({\mathbf {v}}_m(s),{\mathbf {n}}_m(s))] \Vert \le c \left( \Vert {\mathbf {v}}_m(t)\Vert \Vert \nabla {\mathbf {n}}_m(t)\Vert \right) ^{\frac{4-d}{4}}\left( \Vert \nabla {\mathbf {v}}_m(t)\Vert \Vert \nabla ^2 {\mathbf {n}}_m(t)\Vert \right) ^{\frac{d}{4}}. \end{aligned}$$

Thus,

$$\begin{aligned} \begin{aligned}&\Vert {\hat{\pi }}_m[ {\tilde{B}}_{m}({\mathbf {v}}_m(s),{\mathbf {n}}_m(s))] \Vert _{L^\frac{d}{4}(0,T;{\mathbf {L}}^2)}^2\\&\quad \le c \sup _{0\le t\le T}\left( \Vert {\mathbf {v}}_m(t)\Vert \Vert \nabla {\mathbf {n}}_m(t)\Vert \right) ^{\frac{4-d}{2}} \biggl [\int _0^T \Vert \nabla {\mathbf {v}}_m(t)\Vert ^2 dt \biggr ]^{\frac{d}{4}}\\&\qquad \times \biggl [\int _0^T( \Vert {\mathbf {n}}_m(t)\Vert ^2+ \Vert \Delta {\mathbf {n}}_m(t)\Vert ^2) dt \biggr ]^{\frac{d}{4}}. \end{aligned} \end{aligned}$$

Taking the mathematical expectation and using Hölder’s inequality lead to

$$\begin{aligned} \begin{aligned}&\sup _{m\in {\mathbb {N}}}{\mathbb {E}}\Vert {\hat{\pi }}_m[ {\tilde{B}}_{m}({\mathbf {v}}_m(s),{\mathbf {n}}_m(s))] \Vert _{L^\frac{d}{4}(0,T;{\mathbf {L}}^2)}^2\\&\quad \le c \sup _{m \in {\mathbb {N}}} \biggl [{\mathbb {E}}\sup _{0\le t\le T}\Vert {\mathbf {v}}_m(t)\Vert ^{2(4-d)} {\mathbb {E}}\sup _{0\le t\le T}\Vert \nabla {\mathbf {n}}_m(t)\Vert ^{2(4-d)} \biggr ]^\frac{1}{4} \\&\qquad \times \sup _{m \in {\mathbb {N}}}\biggl [{\mathbb {E}}\left( \int _0^T \Vert \nabla {\mathbf {v}}_m(t)\Vert ^2 dt\right) ^d {\mathbb {E}}\left( \int _0^T(\Vert {\mathbf {n}}_m(t)\Vert ^2+ \Vert \Delta {\mathbf {n}}_m(t)\Vert ^2) dt\right) ^d\biggr ]^\frac{1}{4}, \end{aligned} \end{aligned}$$

which along with (3.21), (3.22) and Corollary 3.11 yield

3.31 $$\begin{aligned} \sup _{m\in {\mathbb {N}}}{\mathbb {E}}\Vert J^2_m\Vert ^2_{W^{1,\frac{d}{4}}(0,T;{\mathbf {L}}^2)}= & {} \sup _{m \in {\mathbb {N}}}{\mathbb {E}} \biggl \Vert \int _0^\cdot {\hat{\pi }}_m[ {\tilde{B}}_{m}({\mathbf {v}}_m(s),{\mathbf {n}}_m(s))] ds\biggr \Vert ^2_{W^{1,\frac{d}{4}}(0,T;{\mathbf {L}}^2)}\nonumber \\&\le C, \end{aligned}$$

for some constant $C>0$.

There exists a constant $c>0$ such that for any $m\in {\mathbb {N}}$ and $t\in [0,T]$ we have

$$\begin{aligned} \begin{aligned} \Vert G^2_m({\mathbf {n}}_m(t))\Vert&\le \Vert {\mathbf {h}}\Vert _{{\mathbf {L}}^\infty }\Vert {\mathbf {n}}_m(t)\Vert _{{\mathbf {L}}^\infty }\Vert {\mathbf {n}}_m(t)\Vert ,\\&\le c \Vert {\mathbf {h}}\Vert _{{\mathbf {L}}^\infty } \left( \Vert {\mathbf {n}}_m(t)\Vert ^2 + \Vert {\mathbf {n}}_m(t)\Vert \Vert \nabla {\mathbf {n}}_m(t)\Vert + \Vert {\mathbf {n}}_m(t)\Vert \Vert \Delta {\mathbf {n}}_m(t)\Vert \right) , \end{aligned} \end{aligned}$$

which along with (3.21), (3.22) and Corollary 3.11 yields that there exists a constant $C>0$ such that

3.32 $$\begin{aligned} \sup _{m\in {\mathbb {N}}}{\mathbb {E}}\Vert J^4_m\Vert ^2_{W^{1,2}(0,T;{\mathbf {L}}^2) }=\sup _{m \in {\mathbb {N}}}{\mathbb {E}}\biggl \Vert \frac{1}{2} \int _0^\cdot G^2_m({\mathbf {n}}_m(s)) ds\biggr \Vert ^2_{W^{1,2}(0,T;{\mathbf {L}}^2) }\le C.\qquad \end{aligned}$$

For the polynomial nonlinearity *f* we have: for any $N \in I_d$ there exists a constant $C>0$ such that

3.33 $$\begin{aligned} \sup _{m\in {\mathbb {N}}}{\mathbb {E}} \Vert J^3_m\Vert ^2_{W^{1,2}(0,T; {\mathbf {L}}^2)}&\le C {\mathbb {E}}\left( \int _0^T \Vert f({\mathbf {n}}_m(s))\Vert ^2 ds\right) ^2 \nonumber \\&\le C {\mathbb {E}}\left( \int _0^T ||{\mathbf {n}}_m(s) ||^{4N+2}_{{\mathbf {L}}^{4N+2}} ds\right) ^2\le C T {\mathbb {E}}\sup _{0\le s\le T}||{\mathbf {n}}_m(s)||^{8N+2}_{{\mathbf {H}}^1} \nonumber \\&\le C , \end{aligned}$$

where we have used the continuous embedding ${\mathbf {H}}^1 \subset {\mathbf {L}}^{4N+2}$ and the estimates (3.20) and (3.21) .

For any ${\mathbf {h}}\in {\mathbf {L}}^\infty ({\mathcal {O}})$, using the embedding ${\mathbf {H}}^2\hookrightarrow {\mathbf {L}}^\infty$ we have

3.34 $$\begin{aligned} \Vert {\mathbf {h}}\times {\mathbf {n}}_m(t)\Vert ^p\le \Vert {\mathbf {h}}\Vert _{{\mathbf {L}}^\infty }^p \Vert {\mathbf {n}}_m(t)\Vert ^p, \end{aligned}$$

from which along with [18, Lemma 2.1], (3.34), (3.21) and Corollary 3.11 we derive that there exists a constant $C>0$ such that for any $\alpha \in (0,\frac{1}{2})$ and $p\in [2,\infty )$

3.35 $$\begin{aligned} \sup _{m\in {\mathbb {N}}}{\mathbb {E}} \Vert J^5_m\Vert ^p_{W^{\alpha ,p}(0,T; {\mathbf {L}}^2)}=\sup _{m \in {\mathbb {N}}}{\mathbb {E}}\biggl \Vert \int _0^\cdot G_m({\mathbf {n}}_m(s)) dW_2 \biggr \Vert ^p_{W^{\alpha ,p}(0,T; {\mathbf {L}}^2)} \le C.\qquad \end{aligned}$$

Combining all these estimates complete the proof of our proposition. $\square$

### Tightness and compactness results

This subsection is devoted to the study of the tightness of the Galerkin solutions and derive several weak convergence results. The estimates from the previous subsection play an important role in this part of the paper.

Let $p\in [2,\infty )$ and $\alpha \in (0,\frac{1}{2})$ be as in Proposition 3.12. Let us consider the spaces

$$\begin{aligned} {\mathfrak {X}}_1&= L^2(0,T; \mathrm {V})\cap W^{\alpha , p}(0,T;\mathrm {V}^*),\\ {\mathfrak {Y}}_1&= L^2(0,T;{\mathbf {H}}^2)\cap W^{\alpha , p}(0,T;{\mathbf {L}}^{r}). \end{aligned}$$

Recall that $\mathrm {V}_\beta$, $\beta \in {\mathbb {R}}$, is the domain of the of the fractional power operator $\mathrm {A}^\beta$. Similarly, ${\mathbf {X}}_\beta$ is the domain of $(I+\mathrm {A}_1)^{\beta }$. If $\gamma > \beta$, then the embedding $\mathrm {V}_\gamma \subset \mathrm {V}_\beta$ (resp. ${\mathbf {X}}_\gamma \subset {\mathbf {X}}_\beta$) is compact. We set

$$\begin{aligned} {\mathfrak {X}}_2&=L^\infty (0,T;\mathrm {H})\cap W^{\alpha , p}(0,T; \mathrm {V}^*),\\ {\mathfrak {Y}}_2&=L^\infty (0,T;{\mathbf {H}}^1)\cap W^{\alpha , p}(0,T; {\mathbf {L}}^2), \end{aligned}$$

and for $$\beta \in (0,\frac{1}{2})$$

$$\begin{aligned} {\mathfrak {S}}_1&= L^2(0,T;\mathrm {H})\cap C([0,T];\mathrm {V}_{-\beta }),\\ {\mathfrak {S}}_2&= L^2(0,T;{\mathbf {H}}^1)\cap C([0,T];{\mathbf {X}}_{\beta }). \end{aligned}$$

We shall prove the following important result.

#### Theorem 3.13

Let $p\in [2,\infty )$ and $\alpha \in (0,\frac{1}{2})$ be as in Proposition 3.12 and $\beta \in (0,\frac{1}{2})$ such that $p\beta >1$. The family of laws $\{{\mathcal {L}}({\mathbf {v}}_m, {\mathbf {n}}_m): m\in {\mathbb {N}}\}$ is tight on the Polish space ${\mathfrak {S}}_1\times {\mathfrak {S}}_2$.

#### Proof

We firstly prove that $\{{\mathcal {L}}({\mathbf {v}}_m): m\in {\mathbb {N}}\}$ is tight on $L^2(0,T;\mathrm {H})$. For this aim, we first observe that for a fixed number $R>0$ we have

$$\begin{aligned}&\displaystyle {\mathbb {P}}\left( \Vert {\mathbf {v}}_m\Vert _{{\mathfrak {X}}_1}> R\right) \le {\mathbb {P}}\left( \Vert {\mathbf {v}}_m\Vert _{L^2(0,T;\mathrm {V})}>\frac{R}{2}\right) +{\mathbb {P}}\left( \Vert {\mathbf {v}}_m\Vert _{W^{\alpha ,p}(0,T;\mathrm {V}^*)}>\frac{R}{2}\right) ,\\&\qquad \qquad \qquad \qquad \qquad \displaystyle \le \frac{4}{R^2} {\mathbb {E}}\left( \Vert {\mathbf {v}}_m\Vert ^2_{L^2(0,T;\mathrm {V})}+\Vert {\mathbf {v}}_m\Vert _{W^{\alpha ,p}(0,T;\mathrm {V}^*)}\right) , \end{aligned}$$

from which along with (3.21), (3.23), and (3.24) we infer that

3.36 $$\begin{aligned} \sup _{m\in {\mathbb {N}}}{\mathbb {P}}\left( \Vert {\mathbf {v}}_m\Vert _{{\mathfrak {X}}_1}> R\right) \le \frac{4C}{R^2}. \end{aligned}$$

Since ${\mathfrak {X}}_1$ is compactly embedded into $L^2(0,T;\mathrm {H})$, we conclude that the laws of ${\mathbf {v}}_m$ form a family of probability measures which is tight on $L^2(0,T;\mathrm {H})$. Secondly, the same argument is used to prove that the laws of ${\mathbf {n}}_m$ are tight on $L^2(0,T;{\mathbf {H}}^1)$. Next, we choose $\beta \in (0,\frac{1}{2})$ and $p\in [2,\infty )$ such that $p\beta >1$ is satisfied. By [43, Corollary 5 of Section 8] the spaces ${\mathfrak {X}}_2$ and ${\mathfrak {Y}}_2$ are compactly imbedded in $C([0,T];\mathrm {V}_{-\beta })$ and $C([0,T];{\mathbf {X}}_{\beta })$, respectively. Hence the same argument as above provides us with the tightness of $\{{\mathcal {L}}({\mathbf {v}}_m):m\in {\mathbb {N}}\}$ and $\{{\mathcal {L}}({\mathbf {n}}_m):m\in {\mathbb {N}}\}$ on $C([0,T];\mathrm {V}_{-\beta })$ and $C([0,T];{\mathbf {X}}_{\beta })$. Now we can easily conclude the proof of the theorem. $\square$

Throughout the remaining part of this paper we assume that $\alpha$, *p* and $\beta$ are as in Theorem 3.13. We also use the notation from Remark 2.8.

#### Proposition 3.14

Let ${\mathfrak {S}}={\mathfrak {S}}_1\times {\mathfrak {S}}_2\times C([0,T];\mathrm {K}_2)\times C([0,T];{\mathbb {R}})$. There exist a Borel probability measure $\mu$ on ${\mathfrak {S}}$ and a subsequence of $({\mathbf {v}}_m,{\mathbf {n}}_m, W_1, W_2)$ such that their laws weakly converge to $\mu$.

#### Proof

Thanks to the above lemma the laws of $\{({\mathbf {v}}_m,{\mathbf {n}}_m, W_1, W_2):m\in {\mathbb {N}}\}$ form a tight family on ${\mathfrak {S}}$. Since ${\mathfrak {S}}$ is a Polish space, we get the result from the application of Prohorov’s theorem. $\square$

The following result relates the above convergence in law to almost sure convergence.

#### Proposition 3.15

Let $\alpha , \beta \in (0,\frac{1}{2})$ be as in Theorem 3.13. Then, there exist a complete probability space $(\Omega ^\prime , {\mathcal {F}}^\prime , {\mathbb {P}}^\prime )$ and a sequence of ${\mathfrak {S}}$-valued random variables, denoted by $\{ (\bar{{\mathbf {v}}}_m,\bar{{\mathbf {n}}}_m, W_1^m, W_2^m):m\in {\mathbb {N}}\}$, defined on $(\Omega ^\prime , {\mathcal {F}}^\prime , {\mathbb {P}}^\prime )$ such that their laws are equal to the laws of $\{({\mathbf {v}}_m,{\mathbf {n}}_m, W_1, W_2):m\in {\mathbb {N}}\}$ on ${\mathfrak {S}}$. Also, there exists an ${\mathfrak {S}}$-random variable $({\mathbf {v}}, {\mathbf {n}}, {\bar{W}}_1, {\bar{W}}_2)$ defined on $(\Omega ^\prime , {\mathcal {F}}^\prime , {\mathbb {P}}^\prime )$ such that

3.37 $$\begin{aligned}&{\mathcal {L}}({\mathbf {v}}, {\mathbf {n}}, {\bar{W}}_{1}, {\bar{W}}_{2})=\mu \text { on } {\mathfrak {S}}, \end{aligned}$$

3.38 $$\begin{aligned}&\bar{{\mathbf {v}}}_m\rightarrow {\mathbf {v}}\text { for }m\rightarrow \infty \text { in } L^2(0,T;\mathrm {H}) \,\, {\mathbb {P}}^\prime \text {-a.s.}, \end{aligned}$$

3.39 $$\begin{aligned}&\bar{{\mathbf {v}}}_m\rightarrow {\mathbf {v}}\text { for }m\rightarrow \infty \text { in } C([0,T];\mathrm {V}_{-\beta }) \,\, {\mathbb {P}}^\prime \text {-a.s.}, \end{aligned}$$

3.40 $$\begin{aligned}&\bar{{\mathbf {n}}}_m\rightarrow {\mathbf {n}}\text { for }m\rightarrow \infty \text { in } L^2(0,T;{\mathbf {H}}^1) \,\, {\mathbb {P}}^\prime \text {-a.s.}, \end{aligned}$$

3.41 $$\begin{aligned}&\bar{{\mathbf {n}}}_m\rightarrow {\mathbf {n}}\text { for }m\rightarrow \infty \text { in } C([0,T];{\mathbf {X}}_{\beta }) \,\, {\mathbb {P}}^\prime \text {-a.s.}, \end{aligned}$$

3.42 $$\begin{aligned}&{\bar{W}}_1^m \rightarrow {\bar{W}}_{1}\text { for }m\rightarrow \infty \text { in } C([0,T];\mathrm {K}_2) \,\, {\mathbb {P}}^\prime \text {-a.s.}, \end{aligned}$$

3.43 $$\begin{aligned}&{\bar{W}}_2^m \rightarrow {\bar{W}}_{2}\text { for }m\rightarrow \infty \text { in } C([0,T];{\mathbb {R}}) \,\, {\mathbb {P}}^\prime \text {-a.s.} \end{aligned}$$

#### Proof

Proposition 3.15 is a consequence of Proposition 3.14 and Skorokhod’s Theorem. $\square$

Let $${\mathfrak {X}}_3=L^\infty (0,T;\mathrm {H})\cap L^2(0,T; \mathrm {V})$$ and $${\mathfrak {Y}}_3= L^\infty (0,T;{\mathbf {H}}^1)\cap L^2(0,T; {\mathbf {H}}^2)$$.

#### Proposition 3.16

If all the assumptions of Theorem 3.2 are verified, then for any $p\ge 2$ and $m\in {\mathbb {N}}$ the pair of processes $(\bar{{\mathbf {v}}}_m, \bar{{\mathbf {n}}}_m)$ satisfies the following estimates on the new probability space $(\Omega ^\prime , {\mathcal {F}}^\prime , {\mathbb {P}}^\prime )$:

3.44 $$\begin{aligned}&\sup _{m\in {\mathbb {N}}}\biggl ({\mathbb {E}}^\prime \biggl [ \sup _{t\in [0,T]}\Vert \bar{{\mathbf {n}}}_m(t)\Vert ^p+p \int _0^T \Vert \bar{{\mathbf {n}}}_m(s) \Vert ^{p-2} \Vert \nabla \bar{{\mathbf {n}}}_m(s)\Vert ^2 ds \nonumber \\&\qquad + p \int _0^t \Vert \bar{{\mathbf {n}}}_m(s) \Vert ^{p-2} \Vert \bar{{\mathbf {n}}}_m(s)\Vert ^{2N+2}_{{\mathbf {L}}^{2N+2}} ds\biggr ]\biggr )\le {\mathfrak {G}}_0(T,p), \end{aligned}$$

3.45 $$\begin{aligned}&{\mathbb {E}}^\prime \biggl [ \sup _{0\le s\le T}\left( \Vert \bar{{\mathbf {v}}}_m(s)\Vert ^{2}+{\tilde{\ell }} \Vert \bar{{\mathbf {n}}}_m(s) \Vert ^2+\Vert \nabla \bar{{\mathbf {n}}}_m(s)\Vert ^{2}+\int _{\mathcal {O}}F(\bar{{\mathbf {n}}}_m(s,x)) dx \right) ^p\nonumber \\&\qquad + \int _0^T \left( \Vert \nabla \bar{{\mathbf {v}}}_m(s)\Vert ^2+ \Vert \mathrm {A}_1\bar{{\mathbf {n}}}_m(s)+f(\bar{{\mathbf {n}}}_m(s))\Vert ^2\right) \biggr ]^p \le {\mathfrak {G}}_1(T,p), \end{aligned}$$

3.46 $$\begin{aligned}&{\mathbb {E}}^\prime \biggl [\int _0^T \Vert \mathrm {A}_1\bar{{\mathbf {n}}}_m(s)\Vert ^2 ds\biggr ]^p \le {\mathfrak {G}}_1(T,p\cdot (2N+1)), \end{aligned}$$

where ${\mathfrak {G}}_0(T,p)$, ${\tilde{\ell }}$ and ${\mathfrak {G}}_1(T,p)$ are defined in Propositions 3.8 and 3.9, respectively.

Furthermore, there exists a constant $C>0$ such that

3.47 $$\begin{aligned}&\sup _{m \in {\mathbb {N}}}{\mathbb {E}}^\prime \biggl [\int _0^T \Vert {B}_{m}(\bar{{\mathbf {v}}}_m(t),\bar{{\mathbf {v}}}_m(t) \Vert _{\mathrm {V}^*}^{\frac{4}{d}} dt\biggr ]^\frac{d}{2}\le C, \end{aligned}$$

3.48 $$\begin{aligned}&\sup _{m\in {\mathbb {N}}}{\mathbb {E}}^\prime \biggl [\int _0^T \Vert M_m(\bar{{\mathbf {n}}}_m(t))\Vert _{\mathrm {V}^*}^{\frac{d}{4}} dt\biggr ]^\frac{d}{2} \le C, \end{aligned}$$

3.49 $$\begin{aligned}&\sup _{m\in {\mathbb {N}}}{\mathbb {E}}^\prime \biggl [\int _0^T \Vert {\tilde{B}}_{m}(\bar{{\mathbf {v}}}_m(t), \bar{{\mathbf {n}}}_m(t)) \Vert _{{\mathbf {L}}^2}^\frac{d}{4} dt\biggr ]^\frac{d}{2}\le C, \end{aligned}$$

3.50 $$\begin{aligned}&\sup _{m\in {\mathbb {N}}} {\mathbb {E}}^\prime \int _0^T \Vert f_m(\bar{{\mathbf {n}}}_m(t))\Vert ^r_{{\mathbf {L}}^{r}} dt\le C, \end{aligned}$$

where $$r=\frac{2N+2}{2N+1}$$.

#### Proof

Consider the function $\Phi ({\mathbf {u}}, {\mathbf {e}})$ on ${\mathfrak {X}}_3\times {\mathfrak {Y}}_3\subset {\mathfrak {S}}_1\times {\mathfrak {S}}_2$ defined by

$$\begin{aligned} \Phi ({\mathbf {u}},{\mathbf {e}})=\sup _{0\le s\le T}\left[ \Vert {\mathbf {u}}(s)\Vert ^{2p}+\Vert \nabla {\mathbf {e}}(s)\Vert ^{2p}\right] + {\tilde{\kappa }}_0 \biggl [\int _0^T \left( \Vert \nabla {\mathbf {u}}(s)\Vert ^2+ \Vert \Delta {\mathbf {e}}(s)\Vert ^2\right) ds\biggr ]^p \end{aligned}$$

$\Phi$ is on ${\mathfrak {S}}_1\times {\mathfrak {S}}_2$ a continuous function, thus Borel measurable. Thanks to (3.37) for any $m\in {\mathbb {N}}$ the processes $({\mathbf {v}}_m,{\mathbf {n}}_m)$ and $(\bar{{\mathbf {v}}}_m, \bar{{\mathbf {n}}}_m)$ are identical in law. Therefore, we derive that

$$\begin{aligned} {\mathbb {E}} \Phi ({\mathbf {v}}_m,{\mathbf {n}}_m)= {\mathbb {E}}^\prime \Phi (\bar{{\mathbf {v}}}_m, \bar{{\mathbf {n}}}_m),\quad m\in {\mathbb {N}}, \end{aligned}$$

which altogether with the estimates (3.21), (3.22) and Corollary 3.11 yield (3.45). The estimates (3.47), (3.48) and (3.49) can be shown using similar idea to the proof of (3.28), (3.27), (3.31). The estimate (3.50) easily follows from the continuous embedding ${\mathbf {L}}^2\subset {\mathbf {L}}^r$, $r=\frac{2n+2}{2N+1}\in (1,2)$, and (3.33). $\square$

We prove several convergence results which are for the proof of our existence result.

#### Proposition 3.17

Let $\beta \in (0,\frac{1}{2})$. We can extract a subsequence $\{(\bar{{\mathbf {v}}}_{m_k},\bar{{\mathbf {n}}}_{m_k}):k\in {\mathbb {N}}\}$ from $\{(\bar{{\mathbf {v}}}_m, \bar{{\mathbf {n}}}_m):m\in {\mathbb {N}}\}$ such that

3.51 $$\begin{aligned}&\bar{{\mathbf {v}}}_{m_k}\rightarrow {\mathbf {v}}\,\,\text {strongly in}\,\,L^{2}(\Omega ^\prime \times [0,T];\mathrm {H}\, ), \end{aligned}$$

3.52 $$\begin{aligned}&\bar{{\mathbf {v}}}_{m_k}\rightarrow {\mathbf {v}}\,\,\text {strongly in}\,\,L^4(\Omega ^\prime ; C([0,T];\mathrm {V}_{-\beta })\, ), \end{aligned}$$

3.53 $$\begin{aligned}&\bar{{\mathbf {n}}}_{m_k}\rightarrow {\mathbf {n}}\,\,\text {strongly in}\,\,L^{2}(\Omega ^\prime \times [0,T];{\mathbf {H}}^1\, ), \end{aligned}$$

3.54 $$\begin{aligned}&\bar{{\mathbf {n}}}_{m_k}\rightarrow {\mathbf {n}}\,\,\text {weakly in}\,\,L^{2}(\Omega ^\prime \times [0,T];{\mathbf {H}}^2\, ), \end{aligned}$$

3.55 $$\begin{aligned}&\bar{{\mathbf {n}}}_{m_k}\rightarrow {\mathbf {n}}\,\,\text {strongly in}\,\,L^4(\Omega ^\prime ;C([0,T];{\mathbf {X}}_{\beta })\, ) \end{aligned}$$

3.56 $$\begin{aligned}&\bar{{\mathbf {n}}}_{m_k}\rightarrow {\mathbf {n}}\text { strongly in } {\mathfrak {S}}_2 \,\, {\mathbb {P}}^\prime \text {-a.s.}, \end{aligned}$$

3.57 $$\begin{aligned}&\bar{{\mathbf {n}}}_{m_k}\rightarrow {\mathbf {n}}\text { for almost everywhere } (x,t) \text { and } {\mathbb {P}}^\prime \text {-a.s.}. \end{aligned}$$

#### Proof

From (3.45) and Banach–Alaoglu’s theorem we infer that there exists a subsequence $\bar{{\mathbf {v}}}_{m_k}$ of $\bar{{\mathbf {v}}}_m$ satisfying

3.58 $$\begin{aligned} \bar{{\mathbf {v}}}_{m_k}\rightarrow {\mathbf {v}}\text { weakly in } L^{2p}(\Omega ^\prime ;L^2(0,T;\mathrm {H})), \end{aligned}$$

for any $p\in [2,\infty )$. Now let us consider the positive nondecreasing function $\varphi (x)=x^{2p}$, $p\in [2,\infty )$, defined on ${\mathbb {R}}_+$. The function $\varphi$ obviously satisfies

3.59 $$\begin{aligned} \lim _{x\rightarrow \infty }\frac{\varphi (x)}{x}=\infty . \end{aligned}$$

Thanks to the estimate ${\mathbb {E}}^\prime \sup _{t\in [0,T]}\Vert \bar{{\mathbf {v}}}_{m_k}\Vert ^{2p}\le C$ (see (3.45)), we have

3.60 $$\begin{aligned} \sup _{k\ge 1}{\mathbb {E}}^\prime (\varphi (\Vert \bar{{\mathbf {v}}}_{m_k}\Vert _{L^2(0,T;\mathrm {H})}))<\infty , \end{aligned}$$

which along with the uniform integrability criteria in [27, Chapter 3,Exercice 6] implies that the family $\{\Vert \bar{{\mathbf {v}}}_{m_k}\Vert _{L^2(0,T;\mathrm {H})}:m\in {\mathbb {N}}\}$ is uniform integrable with respect to the probability measure. Thus, we can deduce from Vitali’s Convergence Theorem (see, for instance, [27, Chapter 3,Proposition 3.2]) and (3.38) that

$$\begin{aligned} {\mathbb {E}}^\prime \Vert \bar{{\mathbf {v}}}_{m_k}\Vert ^2_{L^2(0,T;\mathrm {H})} \rightarrow {\mathbb {E}}^\prime \Vert {\mathbf {v}}\Vert ^2_{L^2(0,T;\mathrm {H})}. \end{aligned}$$

From this and (3.58) we derive that

3.61 $$\begin{aligned} \bar{{\mathbf {v}}}_{m_k}\rightarrow {\mathbf {v}}\,\,\text {strongly in}\,\,L^{2}(\Omega ^\prime \times [0,T];\mathrm {H}\, ). \end{aligned}$$

Thanks to (3.40)–(3.43) in Proposition 3.15 and (3.45) we can use the same argument as above to show the convergence (3.52)–(3.55). By the tightness of the laws of $\{\bar{{\mathbf {n}}}_m:m\in {\mathbb {N}}\}$ on ${\mathfrak {S}}_2$ we can extract a subsequence still denoted by $\{\bar{{\mathbf {n}}}_{m_k}:k\in {\mathbb {N}}\}$ such that (3.56) and (3.57) hold. $\square$

The stochastic processes ${\mathbf {v}}$ and ${\mathbf {n}}$ satisfy the following properties.

#### Proposition 3.18

We have

3.62 $$\begin{aligned}&{\mathbb {E}}^\prime \sup _{t\in [0, T]}\Vert {\mathbf {v}}(t)\Vert ^p<\infty , \end{aligned}$$

3.63 $$\begin{aligned}&{\mathbb {E}}^\prime \sup _{t\in [0, T]}\Vert {\mathbf {n}}(t)\Vert ^p_{{\mathbf {H}}^1}<\infty , \end{aligned}$$

for any $$p\in [2, \infty )$$.

#### Proof

One can argue exactly as in [6, Proof of (4.12), page 20], so we omit the details. $\square$

#### Proposition 3.19

Let $d\in \{2,3\}$ and $T\ge 0$. There exist four processes ${\mathfrak {B}}_1, {\mathfrak {M}}\in L^2(\Omega ^\prime ;L^\frac{4}{d}(0,T;\mathrm {V}^*) )$, ${\mathfrak {B}}_2\in L^2(\Omega ^\prime ;L^\frac{d}{4}(0,T;{\mathbf {L}}^2) )$ and ${\mathfrak {f}} \in {\mathbf {L}}^{\frac{2N+2}{2N+1}}(\Omega ^\prime \times [0,T]\times {\mathcal {O}})$ such that

3.64 $$\begin{aligned}&B_{m_k}(\bar{{\mathbf {v}}}_{m_k}, \bar{{\mathbf {v}}}_{m_k}) \rightarrow {\mathfrak {B}}_1, \text { weakly in }L^2(\Omega ^\prime ;L^\frac{d}{4}(0,T;\mathrm {V}^*) ), \end{aligned}$$

3.65 $$\begin{aligned}&M_{m_k}(\bar{{\mathbf {n}}}_{m_k}) \rightarrow {\mathfrak {M}}, \text { weakly in }L^2(\Omega ^\prime ;L^\frac{d}{4}(0,T;\mathrm {V}^*) ),\end{aligned}$$

3.66 $$\begin{aligned}&{\tilde{B}}_{m_k}(\bar{{\mathbf {v}}}_{m_k},\bar{{\mathbf {n}}}_{m_k}) \rightarrow {\mathfrak {B}}_2, \text { weakly in }L^2(\Omega ^\prime ;L^\frac{d}{4}(0,T;{\mathbf {L}}^2) ),\end{aligned}$$

3.67 $$\begin{aligned}&f_{m_k}(\bar{{\mathbf {n}}}_{m_k}) \rightarrow {\mathfrak {f}},\text { weakly in } L^{\frac{2N+2}{2N+1}}(\Omega ^\prime \times [0,T]\times {\mathcal {O}}; {\mathbb {R}}^3). \end{aligned}$$

#### Proof

Note that Proposition 3.16 remains valid with $\bar{{\mathbf {n}}}_m$ replaced by $\bar{{\mathbf {n}}}_{m_k}$. Thus, Proposition 3.19 follows from Eqs. (3.47)–(3.50) and application of Banach–Alaoglu’s theorem. $\square$

### Passage to the limit and the end of proof of Theorem 3.2

In this subsection we prove several convergences which will enable us to conclude that the limiting objects that we found in Proposition 3.15 are in fact a weak martingale solution to our problem.

Proposition 3.17 will be used to prove the following result.

#### Proposition 3.20

For any process $\Psi \in L^2(\Omega ^\prime ;L^\frac{4}{4-d} (0,T;\mathrm {V}))$, the following identity holds

3.68 $$\begin{aligned} \begin{aligned} \lim _{k\rightarrow \infty }{\mathbb {E}}^\prime \int _0^T \langle {B}_{m_k}(\bar{{\mathbf {v}}}_{m_k}(t), \bar{{\mathbf {v}}}_{m_k}(t)), \Psi (t) \rangle _{\mathrm {V}^*,\mathrm {V}} dt&={\mathbb {E}}^\prime \int _0^T \langle {\mathfrak {B}}_1(t), \Psi (t) \rangle _{\mathrm {V}^*,\mathrm {V}}dt,\\&={\mathbb {E}}^\prime \int _0^T \langle B({\mathbf {v}}(t), {\mathbf {v}}(t)), \Psi (t)\rangle _{\mathrm {V}^*,\mathrm {V}} dt. \end{aligned} \end{aligned}$$

#### Proof

Let

$$\begin{aligned} {\mathbb {D}}=\left\{ \Phi =\sum _{i=1}^k \mathbb {1}_{D_i}\mathbb {1}_{J_i} \psi _i: D_i\subset \Omega , J_i\subset [0,T] \text { is measurable}, \psi _i \in {\mathcal {V}}\right\} . \end{aligned}$$

Owing to [49, Proposition 21.23] and the density of ${\mathbb {D}}$ in $L^2(\Omega , {\mathbb {P}};L^\frac{4}{4-d}(0,T;\mathrm {V}))$ (see, for instance, [39, Theorem 3.2.6]), in order to show that the identity (3.68) holds it is enough to check that

$$\begin{aligned} \lim _{k\rightarrow \infty } {\mathbb {E}}^\prime \int _0^T \mathbb {1}_J(t)\mathbb {1}_D \langle {B}_{m_k}(\bar{{\mathbf {v}}}_{m_k}(t), \bar{{\mathbf {v}}}_{m_k}(t))-B({\mathbf {v}}(t), {\mathbf {v}}(t) ), \psi \rangle _{\mathrm {V}^*,\mathrm {V}} dt= 0, \end{aligned}$$

for any $\Phi =\mathbb {1}_D 1_J \psi \in {\mathbb {D}}$. For this purpose we first note that

$$\begin{aligned} \begin{aligned} \left\langle {B}_{m_k}(\bar{{\mathbf {v}}}_{m_k},\bar{{\mathbf {v}}}_{m_k}(t))-B({\mathbf {v}}, {\mathbf {v}}), \psi \right\rangle _{\mathrm {V}^*,\mathrm {V}}&= \left\langle {\tilde{B}}_{m_k}(\bar{{\mathbf {v}}}_{m_k}-{\mathbf {v}}, \bar{{\mathbf {v}}}_{m_k}),\psi \right\rangle _{\mathrm {V}^*,\mathrm {V}}\\&\quad + \left\langle {\tilde{B}}_{m_k}({\mathbf {v}}, \bar{{\mathbf {v}}}_{m_k}-{\mathbf {v}}),\psi \right\rangle _{\mathrm {V}^*,\mathrm {V}},\\&= I_1+I_2. \end{aligned} \end{aligned}$$

The mapping $\langle {B}_{m_k}({\mathbf {u}}, \cdot ),\psi \rangle _{\mathrm {V}^*,\mathrm {V}}$ from $L^2(\Omega ^\prime ; L^2(0,T;\mathrm {V})$ into $L^2(\Omega ^\prime ;L^\frac{4}{d}(0,T;{\mathbb {R}}))$ is linear and continuous. Therefore if $\bar{{\mathbf {v}}}_{m_k}$ converges to ${\mathbf {v}}$ weakly in $L^2(\Omega ^\prime ; L^2(0,T;\mathrm {V})$ then $I_2$ converges to 0 weakly in $L^2(\Omega ^\prime ;L^\frac{4}{d}(0,T;{\mathbb {R}}))$. To deal with $I_1$ we recall that

$$\begin{aligned} \begin{aligned}&\biggl |{\mathbb {E}}^\prime \int _0^T \mathbb {1}_J\mathbb {1}_D (\omega ^\prime ,t)\langle {B}_{m_k}(\bar{{\mathbf {v}}}_{m_k}(t)-{\mathbf {v}}(t), \bar{{\mathbf {v}}}_{m_k}(t)),\psi \rangle _{\mathrm {V}^*,\mathrm {V}} dt \biggr |\\&\quad \le \Vert \psi \Vert _{{\mathbb {L}}^\infty } \biggl [{\mathbb {E}}^\prime \int _0^T\Vert \nabla \bar{{\mathbf {v}}}_{m_k}(t)\Vert ^2 dt \biggr ]^\frac{1}{2} \\&\qquad \times \biggl [{\mathbb {E}}^\prime \int _0^T\Vert \bar{{\mathbf {v}}}_{m_k}(t)-{\mathbf {v}}(t)\Vert ^2 dt \biggr ]^\frac{1}{2}. \end{aligned} \end{aligned}$$

Thanks to (3.45) and the convergence (3.51) we see that the right-hand side of above inequality converges to 0 as $m_k$ goes to infinity. Hence $I_1$ converges to 0 weakly in $L^2(\Omega ^\prime ;L^\frac{4}{d}(0,T;{\mathbb {R}}))$. This ends the proof of our proposition. $\square$

In the next proposition we will prove that ${\mathfrak {M}}$ coincides with $M({\mathbf {n}})$.

#### Proposition 3.21

Assume that $d<4$. For any process $\Psi \in L^2(\Omega ^\prime ;L^\frac{4}{4-d} (0,T;\mathrm {V}))$, the following identity holds

3.69 $$\begin{aligned} \begin{aligned} {\mathbb {E}}^\prime \int _0^T \langle {\mathfrak {M}}(t), \Psi (t) \rangle _{\mathrm {V}^*,\mathrm {V}}dt ={\mathbb {E}}^\prime \int _0^T \langle M({\mathbf {n}}(t)), \Psi (t)\rangle _{\mathrm {V}^*,\mathrm {V}} dt. \end{aligned} \end{aligned}$$

#### Proof

Since $\pi _m$ strongly converges to the identity operator *Id* in $L^2(\Omega ^\prime ;L^\frac{d}{4}(0,T;\mathrm {V}^*)),$ it is enough to show that (3.69) is true for $M(\bar{{\mathbf {n}}}_{m_k}(t))$ in place of $M_{m_k}(\bar{{\mathbf {n}}}_{m_k}(t))$. By the relation (2.5) we have

3.70 $$\begin{aligned} \begin{aligned}&\langle M(\bar{{\mathbf {n}}}_{m_k}(t))-M({\mathbf {n}}(t)), \psi \rangle _{\mathrm {V}^*,\mathrm {V}}\\&\quad =\sum _{i,j,k}\int _{\mathcal {O}}\partial _{x_j} \psi ^i \partial _{x_i} \bar{{\mathbf {n}}}_{m_k}^{(k)}(t) \left( \partial _{x_j}\bar{{\mathbf {n}}}_{m_k}^{(k)}(t)-\partial _{x_i}{\mathbf {n}}^{(k)}(t) \right) dx\\&\qquad +\sum _{i,j,k}\int _{\mathcal {O}}\partial _{x_j}\psi ^i \partial _{x_j}{\mathbf {n}}^{(k)}(t) \left( \partial _{x_i}\bar{{\mathbf {n}}}_{m_k}^{(k)}(t)-\partial _{x_i}{\mathbf {n}}^{(k)}(t) \right) dx, \end{aligned} \end{aligned}$$

for any $\psi \in {\mathcal {V}}$. From this inequality we infer that

3.71 $$\begin{aligned} \begin{aligned}&\biggl |{\mathbb {E}}^\prime \int _0^T \mathbb {1}_J(\omega ^\prime ,t)\langle M(\bar{{\mathbf {n}}}_{m_k}(t))-M({\mathbf {n}}(t)), \psi \rangle _{\mathrm {V}^*,\mathrm {V}}dt \biggl |\\&\quad \le C \Vert \nabla \psi \Vert \biggl [{\mathbb {E}}^\prime \int _0^T \Vert \nabla (\bar{{\mathbf {n}}}_{m_k}(t)-{\mathbf {n}}(t))\Vert ^2 dt \biggr ]^\frac{1}{2} \\&\qquad \times \biggl (\biggl [{\mathbb {E}}^\prime \sup _{0\le t \le T}\Vert \nabla \bar{{\mathbf {n}}}_{m_k}(t)\Vert ^2\biggr ]^\frac{1}{2} + \biggl [{\mathbb {E}}^\prime \sup _{0\le t\le T}\Vert \nabla {\mathbf {n}}(t)\Vert ^2\biggr ]^\frac{1}{2} \biggr ). \end{aligned} \end{aligned}$$

Owing to the estimate (3.45) and the convergence (3.53) we infer that the left hand side of the last inequality converges to 0 as $m_k$ goes to infinity. Now, arguing as in the proof of (3.68) we easily conclude the proof of the proposition. $\square$

#### Proposition 3.22

Let $$d\in \{2,3\}$$. Then,

$$\begin{aligned} {\mathfrak {B}}_2= {\tilde{B}}({\mathbf {v}}, {\mathbf {n}}) \text { in } L^2(\Omega ^\prime ;L^\frac{d}{4}(0,T;{\mathbf {L}}^2) ). \end{aligned}$$

#### Proof

The statement in the proposition is equivalent to say that $\{{\tilde{B}}_{m_k}(\bar{{\mathbf {v}}}_{m_k}(t), \bar{{\mathbf {n}}}_{m_k}(t)):k\in {\mathbb {N}}\}$ converges to ${\tilde{B}}({\mathbf {v}}(t),{\mathbf {n}}(t))$ weakly in $L^2(\Omega ^\prime ;L^\frac{d}{4}(0,T;{\mathbf {L}}^2) )$ as $k\rightarrow \infty$ . To prove this we argue as above, but we consider the set

$$\begin{aligned} {\mathbb {D}}=\{\Phi =\mathbb {1}_J\mathbb {1}_D \mathbb {1}_K: J\subset \Omega ^\prime , D\subset [0,T], K\subset {\mathcal {O}}\text { is measurable}\}. \end{aligned}$$

For any $\Phi \in {\mathbb {D}}$ we have

3.72 $$\begin{aligned} \begin{aligned}&\biggl |{\mathbb {E}}^\prime \int _{[0,T]\times {\mathcal {O}}} {\tilde{B}}_{m_k}(\bar{{\mathbf {v}}}_{m_k}(t),\bar{{\mathbf {n}}}_{m_k}(t))-{\tilde{B}}({\mathbf {v}}(t), {\mathbf {n}}(t))\Phi (\omega ^\prime ,t,x)dxdt\biggr |\\&\quad \le \biggl [{\mathbb {E}}^\prime \int _0^T \Vert \bar{{\mathbf {v}}}_{m_k}(t)-{\mathbf {v}}(t)\Vert ^2dt \biggr ]^\frac{1}{2} \biggl [{\mathbb {E}}^\prime \int _0^T \Vert \nabla \bar{{\mathbf {n}}}_{m_k}(t)\Vert ^2 dt\biggr ]^\frac{1}{2}\\&\qquad +\biggl [{\mathbb {E}}^\prime \int _0^T \Vert {\mathbf {v}}(t)\Vert ^2 dt\biggr ]^\frac{1}{2} \biggl [{\mathbb {E}}^\prime \int _0^T \Vert \nabla \left( \bar{{\mathbf {v}}}_{m_k}(t)-{\mathbf {v}}(t)\right) \Vert ^2dt \biggr ]^\frac{1}{2} \end{aligned} \end{aligned}$$

Thanks to (3.45) and (3.53) we deduce that the left hand side of the last inequality converges to 0 as $m_k$ goes to infinity. This proves our claim. $\square$

The following convergence is also important.

#### Proposition 3.23

Let *r* be as in Proposition 3.12, *i.e.*, $r=\frac{2N+2}{2N+1}\in (1,2)$, and $\beta \in (0,1)$. Then,

3.73 $$\begin{aligned} {\mathfrak {f}} = f({\mathbf {n}}) \text { in } L^r(\Omega ^\prime \times [0,T]\times {\mathcal {O}}; {\mathbb {R}}^3). \end{aligned}$$

#### Proof

To prove (3.73), first remark that by definition the embedding ${\mathbf {X}}_{\beta } \subset {\mathbf {L}}^r$ is continuous for any $\beta \in (0,\frac{1}{2})$. The convergence (3.57) implies that for any $k=0,\ldots ,N$

3.74 $$\begin{aligned} \vert \bar{{\mathbf {n}}}_{m_k}\vert ^{2k} \bar{{\mathbf {n}}}_{m_k}\rightarrow \vert {\mathbf {n}}\vert ^{2k} {\mathbf {n}}\text { for almost everywhere } (x,t) \text { and } {\mathbb {P}}^\prime \text {-a.s.}. \end{aligned}$$

Since $f(\bar{{\mathbf {n}}}_{m_k})$ is bounded in $L^r(\Omega ^\prime \times [0,T]\times {\mathcal {O}}; {\mathbb {R}}^3)$ we can infer from [34, Lemma 1.3, pp. 12] and the convergence (3.74) that

$$\begin{aligned} f(\bar{{\mathbf {n}}}_{m_k})\rightarrow f({\mathbf {n}}) \text { weakly in } L^r(\Omega ^\prime \times [0,T]\times {\mathcal {O}}; {\mathbb {R}}^3) , \end{aligned}$$

which with the uniqueness of weak limit implies the sought result. $\square$

To simplify notation let us define the processes ${\mathcal {M}}^1_{m_k}(t)$ and ${\mathcal {M}}^2_{m_k}(t)$, $t \in [0,T]$ by

$$\begin{aligned} {\mathcal {M}}_{m_k}^1(t)&=\bar{{\mathbf {v}}}_{m_k}(t)-\bar{{\mathbf {v}}}_{m_k}(0)\\&\quad +\int _0^t\biggl (\mathrm {A}\bar{{\mathbf {v}}}_{m_k}(s) +{\tilde{B}}_{m_k}(\bar{{\mathbf {v}}}_{m_k}(s),\bar{{\mathbf {v}}}_{m_k}(s))-M_{m_k}(\bar{{\mathbf {n}}}_{m_k}(s)) \biggr )ds, \end{aligned}$$

and

$$\begin{aligned} \begin{aligned}&{\mathcal {M}}^2_{m_k}(t)=\bar{{\mathbf {n}}}_{m_k}(t)-\bar{{\mathbf {n}}}_{m_k}(0)+\int _0^t \biggl (\mathrm {A}_1\bar{{\mathbf {n}}}_{m_k}(s)+{\tilde{B}}_{m_k}(\bar{{\mathbf {v}}}_{m_k}(s),\bar{{\mathbf {n}}}_{m_k}(s))-f_{m_k}(\bar{{\mathbf {n}}}_{m_k}(s))\biggr )ds\\&\qquad -\int _0^tG^2_{m_k}(\bar{{\mathbf {n}}}_{m_k}(s))ds. \end{aligned} \end{aligned}$$

#### Proposition 3.24

Let ${\mathcal {M}}^1(t)$ and ${\mathcal {M}}^{2}(t)$, $t \in [0,T]$, be defined by

3.75 $$\begin{aligned}&{\mathcal {M}}^1(t)={\mathbf {v}}(t)-{\mathbf {v}}_0+\int _0^t\biggl (\mathrm {A}{\mathbf {v}}(s)+B({\mathbf {v}}(s),{\mathbf {v}}(s))-M({\mathbf {n}}(s))\biggr )ds, \end{aligned}$$

3.76 $$\begin{aligned}&{\mathcal {M}}^2(t)={\mathbf {n}}(t)-{\mathbf {n}}_0+\int _0^t\biggl (\mathrm {A}_1{\mathbf {n}}(s)+{\tilde{B}}({\mathbf {v}}(s),{\mathbf {n}}(s))-f({\mathbf {n}}(s))\biggr )-\int _0^t G^2({\mathbf {n}}(s)) ds, \end{aligned}$$

for any $t\in (0,T]$. Then, for any $t\in (0,T]$

$$\begin{aligned} {\mathcal {M}}_{m_k}^1(t) \text { converges weakly in } L^2(\Omega ^\prime ;\mathrm {V}^*) \text { to } {\mathcal {M}}^1(t),\\ {\mathcal {M}}_{m_k}^2(t) \text { converges weakly in } L^2(\Omega ^\prime ;{\mathbf {L}}^2) \text { to } {\mathcal {M}}^2(t),\\ \end{aligned}$$

as $$k \rightarrow \infty $$.

#### Proof

Let $t\in (0,T]$, we first prove that ${\mathcal {M}}^1_{m_k}(t)\rightarrow {\mathcal {M}}^1(t)$ weakly in $L^2(\Omega ^\prime ;\mathrm {V}^*)$ as *k* goes to infinity. To this end we take an arbitrary $\xi \in L^2(\Omega ^\prime ;\mathrm {V})$. We have

$$\begin{aligned} \begin{aligned}&{\mathbb {E}}^\prime \biggl [\langle {\mathcal {M}}^1_{m_k}(t), \xi \rangle _{\mathrm {V}^*,\mathrm {V}} \biggr ] \\&\quad = {\mathbb {E}}^\prime \biggl [\langle \bar{{\mathbf {v}}}_{m_k}(t)-\bar{{\mathbf {v}}}_{m_k}(0), \xi \rangle - \int _0^t \langle \nabla \bar{{\mathbf {v}}}_{m_k}(s),\nabla \xi \rangle ds-\int _0^t \langle M_{m_k}(\bar{{\mathbf {n}}}_{m_k}(s)),\xi \rangle _{\mathrm {V}^*,\mathrm {V}} \,ds\biggr ]\\&\qquad +{\mathbb {E}}^\prime \biggl [\int _0^t \langle B_{m_k}(\bar{{\mathbf {v}}}_{m_k}(s), \bar{{\mathbf {v}}}_{m_k}(s)), \xi \rangle _{\mathrm {V}^*,\mathrm {V}}\, ds \biggr ]. \end{aligned} \end{aligned}$$

Thanks to the pointwise convergence in $C([0,T];\mathrm {V}_{-\beta })$, thus in $C([0,T];\mathrm {V}^*)$, and the convergences (3.64), (3.68), (3.65) and (3.69) we obtain

$$\begin{aligned}&\lim _{m\rightarrow \infty } {\mathbb {E}}^\prime \biggl [\langle {\mathcal {M}}^1_{m_k}(t), \xi \rangle _{\mathrm {V}^*,\mathrm {V}}\biggr ]\\&\quad = {\mathbb {E}}^\prime \biggl [\langle {\mathbf {v}}(t)-{\mathbf {v}}_0, \xi \rangle - \int _0^t \langle \nabla {\mathbf {v}}(s),\nabla \xi \rangle ds-\int _0^t \langle M({\mathbf {n}}(s)),\xi \rangle _{\mathrm {V}^*,\mathrm {V}} ds\biggr ]\\&\qquad +\, {\mathbb {E}}^\prime \biggl [\int _0^t \langle B({\mathbf {v}}(s), {\mathbf {v}}(s)), \xi \rangle _{\mathrm {V}^*,\mathrm {V}} ds \biggr ], \end{aligned}$$

which proves the sought convergence.

Second, we prove that for any $$t\in (0,T]$$$${\mathcal {M}}^2_{m_k}(t)\rightarrow {\mathcal {M}}^2(t)$$ weakly in $$L^2(\Omega ^\prime ;{\mathbf {L}}^2)$$ as *k* tends to infinity. For this purpose, observe that $$G^2_{m_k}(\cdot )$$ is a linear mapping from $$L^2(\Omega ^\prime ; C([0,T];{\mathbf {L}}^2))$$ into itself and it satisfies

3.77 $$\begin{aligned} {\mathbb {E}}^\prime \Vert G^2_{m_k}({\mathbf {n}})\Vert ^p_{C([0,T];{\mathbf {L}}^2)}\le c \Vert {\mathbf {h}}\Vert ^2_{{\mathbf {L}}^\infty } {\mathbb {E}}^\prime \Vert {\mathbf {n}}\Vert ^p_{C([0,T];{\mathbf {L}}^2)}, \end{aligned}$$

for any $p\in [2,\infty )$. So it is not difficult to show that

3.78 $$\begin{aligned} G_{m_k}(\bar{{\mathbf {n}}}_{m_k})\rightarrow G({\mathbf {n}}) \text { strongly in } L^2(\Omega ^\prime ;C([0,T];{\mathbf {L}}^2)). \end{aligned}$$

Thanks to this observation, the convergences (3.55), (3.73), (3.66) and Proposition 3.22 we can use the same argument as above to show that

3.79 $$\begin{aligned} \lim _{m\rightarrow \infty } \langle {\mathcal {M}}^2_{m_k}(t), \xi \rangle =\langle {\mathcal {M}}^2(t),\xi \rangle , \end{aligned}$$

for any $t\in (0,T]$ and $\xi \in L^2(\Omega ^\prime ;{\mathbf {L}}^{2})$. This completes the proof of Proposition 3.24. $\square$

Let ${\mathcal {N}}$ be the set of null sets of ${\mathcal {F}}^\prime$ and for any $t\ge 0$ and $k\in {\mathbb {N}}$, let

3.80 $$\begin{aligned}&\hat{{\mathcal {F}}}^{m_k}_t:=\sigma \biggl (\sigma \biggl ((\bar{{\mathbf {v}}}_{m_k}(s), \bar{{\mathbf {n}}}_{m_k}(s), {\bar{W}}_1^{m_k}(s), {\bar{W}}_2^{m_k}(s)); s\le t\biggr )\cup {\mathcal {N}}\biggr ),\nonumber \\&{{\mathcal {F}}}^\prime _t:=\sigma \biggl (\sigma \Big (({\mathbf {v}}(s),{\mathbf {n}}(s), {\bar{W}}_1(s),{\bar{W}}_2(s)); s\le t\Big )\cup {\mathcal {N}}\biggr ). \end{aligned}$$

Let us also define the stochastic processes ${\mathfrak {M}}^1_{m_k}$ and ${\mathfrak {M}}^2_{m_k}$ by

$$\begin{aligned}&{\mathfrak {M}}_{m_k}^1(t)= \int _0^t S_{m_k}(\bar{{\mathbf {v}}}_{m_k}(s)) d{\bar{W}}_{1}^{m_k}(s)\\&{\mathfrak {M}}^2_{m_k}(t)=\int _0^t G_{m_k}(\bar{{\mathbf {n}}}_{m_k}(s)) d{\bar{W}}_{2}^{m_k}(s), \end{aligned}$$

for any $$t\in [0,T]$$.

From Proposition 3.24 we see that $({\mathbf {v}}, {\mathbf {n}})$ is a solution to our problem if we can show that the processes ${\bar{W}}_{1}$ and ${\bar{W}}_{2}$ defined in Proposition 3.15 are Wiener processes and ${\mathcal {M}}^1$, ${\mathcal {M}}^2$ are stochastic integrals with respect to ${\bar{W}}_{1}$ and ${\bar{W}}_{2}$ with integrands $(S({\mathbf {v}}(t)))_{t\in [0,T]}$ and $(G({\mathbf {n}}(t)))_{t\in [0,T]}$, respectively. These will be the subjects of the following two propositions.

#### Proposition 3.25

We have the following facts:

1. the stochastic process $\left( {\bar{W}}_{1}(t)\right) _{t\in [0,T]}$ (resp. $\left( {\bar{W}}_{2}(t)\right) _{t\in [0,T]}$) is a $\mathrm {K}_1$-cylindrical $\mathrm {K}_2$-valued Wiener process (resp. ${\mathbb {R}}$-valued standard Brownian motion) on $(\Omega ^\prime , {\mathcal {F}}^\prime , {\mathbb {P}}^\prime )$.
2. For any *s* and *t* such that $0\le s<t\le T$, the increments ${\bar{W}}_{1}(t)-{\bar{W}}_{1}(s)$ and ${\bar{W}}_{2}(t)-{\bar{W}}_{2}(s)$ are independent of the $\sigma$-algebra generated by ${\mathbf {v}}(r), \,\,{\mathbf {n}}(r), \,\, {\bar{W}}_{1}(r),\,\, {\bar{W}}_{2}(r)$, $r\in [0,s]$.
3. Finally, ${\bar{W}}_{1}$ and ${\bar{W}}_{2}$ are mutually independent.

#### Proof

We will just establish the proposition for ${\bar{W}}_{1}$, the same method applies to ${\bar{W}}_{2}$. To this end we closely follow [6], but see also [36, Lemma 9.9] for an alternative proof.

*Proof of item (1).* By Proposition 3.15 the laws of $({\mathbf {v}}_{m_k}, {\mathbf {n}}_{m_k}, W_1, W_2)$ are equal to those of the stochastic process $(\bar{{\mathbf {v}}}_{m_k},\bar{{\mathbf {n}}}_{m_k}, {\bar{W}}_{1}^{m_k},{\bar{W}}_{2}^{m_k})$ on ${\mathfrak {S}}$. Hence, it is easy to check that ${\bar{W}}_{1}^{m_k}$ (resp. ${\bar{W}}_{2}^{m_k}$) form a sequence of $\mathrm {K}_1$-cylindrical $\mathrm {K}_2$-valued Wiener process (resp. ${\mathbb {R}}$-valued Wiener process). Moreover, for $0\le s<t\le T$ the increments ${\bar{W}}_{1}^{m_k}(t)-{\bar{W}}_{1}^{m_k}(s)$ (resp. ${\bar{W}}_{2}^{m_k}(t)-{\bar{W}}_{2}^{m_k}(s)$) are independent of the $\sigma$-algebra generated by the stochastic process $\left( \bar{{\mathbf {v}}}_{m_k}(r),\bar{{\mathbf {n}}}_{m_k}(r), {\bar{W}}_{1}^{m_k}(r), {\bar{W}}_{2}^{m_k}(r)\right)$, for $r\in [0,s]$.

Now, we will check that ${\bar{W}}_{1}$ is a $\mathrm {K}_1$-cylindrical $\mathrm {K}_2$-valued Wiener process by showing that the characteristic function of its finite dimensional distributions is equal to the characteristic function of a Gaussian random variable. For this purpose let $k\in {\mathbb {N}}$ and $s_0=0<s_1<\dots <s_k\le T$ be a partition of [0, *T*]. For each ${\mathbf {u}}\in \mathrm {K}_2^*$ we have

$$\begin{aligned} {\mathbb {E}}^\prime \biggl [e^{i\sum _{j=1}^k {\langle \,{\mathbf {u}}, {\bar{W}}_{1}^{m_k}(s_j)-{\bar{W}}_{1}^{m_k}(s_{j-1})\rangle }_{\mathrm {K}_2^*,\mathrm {K}_2}}\biggr ] =e^{-\frac{1}{2} \sum _{j=1}^k \,(s_j-s_{j-1}) \vert {\mathbf {u}}\vert ^2_{\mathrm {K}_1} }, \end{aligned}$$

where $i^2=-1$. Thanks to (3.42) and the Lebesgue Dominated Convergence Theorem, we have

$$\begin{aligned} \begin{aligned} \lim _{m\rightarrow \infty }{\mathbb {E}}^\prime \biggl [e^{i\sum _{j=1}^k {\langle \,{\mathbf {u}}, {\bar{W}}_{1}^{m_k}(s_j)-{\bar{W}}_{1}^{m_k}(s_{j-1})\rangle }_{\mathrm {K}_2^*,\mathrm {K}_2}}\biggr ]&= {\mathbb {E}}^\prime \biggl [e^{i\sum _{j=1}^k {\langle \,{\mathbf {u}}, {\bar{W}}_{1}(s_j)-{\bar{W}}_{1}(s_{j-1})\rangle }_{\mathrm {K}_2^*,\mathrm {K}_2}}\biggr ]\\&= e^{-\frac{1}{2} \sum _{j=1}^k (s_j-s_{j-1})\vert {\mathbf {u}}\vert ^2_{\mathrm {K}_1}} \end{aligned} \end{aligned}$$

from which we infer that the finite dimensional distributions of ${\bar{W}}_{1}$ follow a Gaussian distribution. The same idea can be carried out to prove that the finite dimensional distributions of ${\bar{W}}_{2}$ are Gaussian.

*Proof of item (2).* Next, we prove that the increments ${\bar{W}}_{1}(t)-{\bar{W}}_{1}(s)$ and ${\bar{W}}_{2}(t)-{\bar{W}}_{2}(s)$, $0\le s<t\le T$ are independent of the $\sigma$-algebra generated by $\left( {\mathbf {v}}(r), \,\,{\mathbf {n}}(r), \,\, {\bar{W}}_{1}(r),\,\, {\bar{W}}_{2}(r)\right)$ for $r\in [0,s]$. To this end, let us consider $\{\phi _j: j=1,\dots ,k\}\subset C_b(\mathrm {V}_{-\beta }\times {\mathbf {H}}^1)$ and $\{\psi _j: j=1,\dots , k\}\subset C_b(\mathrm {K}_2\times {\mathbb {R}})$, where for any Banach space ${\mathbf {B}}$ the space $C_b({\mathbf {B}})$ is defined

$$\begin{aligned} C_b({\mathbf {B}})=\{\phi : {\mathbf {B}}\rightarrow {\mathbb {R}}, \phi \text { is continuous and bounded}\}. \end{aligned}$$

Also, let $0\le r_1<\dots<r_k\le s<t\le T$, $\psi \in C_b(\mathrm {K}_2)$, and $\zeta \in C_b({\mathbb {R}})$. For each $k\in {\mathbb {N}}$, there holds

$$\begin{aligned} \begin{aligned}&{\mathbb {E}}^\prime \biggl [\biggl (\prod _{j=1}^k \phi _j(\bar{{\mathbf {v}}}_{m_k}(r_j),\bar{{\mathbf {n}}}_{m_k}(r_j))\prod _{j=1}\psi _j({\bar{W}}_{1}^{m_k}(r_j), {\bar{W}}_{2}^{m_k}(r_j) ) \biggr )\\&\qquad \times \psi ({\bar{W}}_{1}^{m_k}(t)-{\bar{W}}_{1}^{m_k}(s))\zeta ({\bar{W}}_{2}^{m_k}(t)-{\bar{W}}_{2}^{m_k}(s)) \biggr ]\\&\quad = {\mathbb {E}}^\prime \biggl [\prod _{j=1}^k \phi _j(\bar{{\mathbf {v}}}_{m_k}(r_i), \bar{{\mathbf {n}}}_{m_k}(r_j))\prod _{j=1}\psi _j({\bar{W}}_{1}^{m_k}(r_j), {\bar{W}}_{2}^{m_k}(r_j))\biggr ]\\&\qquad \times {\mathbb {E}}^\prime \left( \zeta ({\bar{W}}_{1}^{m_k}(t)-{\bar{W}}_{1}^{m_k}(s))\right) {\mathbb {E}}^\prime \left( \psi ({\bar{W}}_{2}^{m_k}(t)-{\bar{W}}_{2}^{m_k}(s))\right) . \end{aligned} \end{aligned}$$

Thanks to (3.39), (3.41), (3.42), (3.43) and the Lebesgue Dominated Convergence Theorem, the same identity is true with $({\mathbf {v}}, {\mathbf {n}}, {\bar{W}}_{1}, {\bar{W}}_{2})$ in place of $(\bar{{\mathbf {v}}}_{m_k}, \bar{{\mathbf {n}}}_{m_k}, {\bar{W}}_{1}^{m_k}, {\bar{W}}_{2}^{m_k})$. This completes the proof of the second item of the proposition.

*Proof of item (3).* By using the characteristic functions of the process ${\bar{W}}_{1}^{m_k}, {\bar{W}}_{2}^{m_k}, {\bar{W}}_{1}$ and ${\bar{W}}_{2}$ , item (3) can be easily proved as in the proof of item (1), so we omit the details. $\square$

#### Proposition 3.26

For each $t\in (0,T]$ we have

3.81 $$\begin{aligned}&{\mathcal {M}}^1(t)=\int _0^t S({\mathbf {v}}(s))d{\bar{W}}_{1}(s) \text { in } L^2(\Omega ^\prime , \mathrm {V}^*), \end{aligned}$$

3.82 $$\begin{aligned}&{\mathcal {M}}^2(t)=\int _0^t ({\mathbf {n}}(s)\times {\mathbf {h}}) d{\bar{W}}_{2}(s) \text { in } L^2(\Omega , {\mathbf {X}}_{\beta }). \end{aligned}$$

#### Proof

The same argument given in [6] can be used without modification to establish (3.82), thus we only prove (3.81). The proof we give below can also be adapted to the proof of (3.82).

We will closely follow the idea in [4] to establish (3.81). For this purpose, let us fix $t\in (0,T]$ and for any $\varepsilon >0$ let $\eta _\varepsilon :{\mathbb {R}} \rightarrow {\mathbb {R}}$ be a standard mollifier with support in (0, *t*). For $R\in \{S, S_{m_k}\}$, ${\mathbf {u}}\in \{\bar{{\mathbf {v}}}_{m_k}, {\mathbf {v}}\}$ and $s\in (0,t]$ let us set

$$\begin{aligned} R^\varepsilon ({\mathbf {u}}(s))&= (\eta _\varepsilon \star R({\mathbf {u}}(\cdot )))(s)\\&= \int _{-\infty }^{\infty } \eta _\varepsilon (s-r) R({\mathbf {u}}(r)) dr. \end{aligned}$$

We recall that, since *R* is Lipschitz, $R^\varepsilon$ is Lipschitz. We also have the following two important facts, see, for instance, [2, Section 1.3]:

- for any $p \in [1,\infty )$ there exists a constant $C>0$ such that for any $\varepsilon >0$ we have
  3.83 $$\begin{aligned} \int _0^t \Vert R^\varepsilon ({\mathbf {u}}(s)) \Vert ^p_{{\mathcal {T}}_2(\mathrm {K}_1,\mathrm {H})} ds \le C \int _0^t \Vert R({\mathbf {u}}(s)) \Vert ^p_{{\mathcal {T}}_2(\mathrm {K}_1,\mathrm {H})} ds. \end{aligned}$$
- For any $p \in [1,\infty )$, we have
  3.84 $$\begin{aligned} \lim _{\varepsilon \rightarrow 0} \int _0^t \Vert R^\varepsilon ({\mathbf {u}}(s))-R({\mathbf {u}}(s)) \Vert ^p_{{\mathcal {T}}_2(\mathrm {K}_1,\mathrm {H})} ds=0. \end{aligned}$$

Now, let ${\mathcal {M}}^\varepsilon _{m_k}$ and ${\mathcal {M}}^\varepsilon$ be respectively defined by

$$\begin{aligned}&{\mathcal {M}}^\varepsilon _{m_k}(t)= \int _0^t S^\varepsilon _{m_k}(\bar{{\mathbf {v}}}_{m_k}(s)) d{\bar{W}}_{1}^{m_k}(s),\\&{\mathcal {M}}^\varepsilon (t)=\int _0^t S^\varepsilon ({\mathbf {v}}(s))d{\bar{W}}_{1}(s), \end{aligned}$$

for $t\in (0,T]$. From the Itô isometry, (3.83) and some elementary calculations we infer that there exists a constant $C>0$ such that for any $\varepsilon >0$ and $m_k\in {\mathbb {N}}$

3.85 $$\begin{aligned} {\mathbb {E}}^\prime \Vert {\mathcal {M}}_{m_k}(t)-{\mathcal {M}}^\varepsilon _{m_k}(t)\Vert ^2&= {\mathbb {E}}^\prime \int _0^t \Vert S(\bar{{\mathbf {v}}}_{m_k}(s))-S_{m_k}^\varepsilon (\bar{{\mathbf {v}}}_{m_k}(s)) \Vert ^2_{{\mathcal {T}}_2(\mathrm {K}_1,\mathrm {H})} ds,\nonumber \\&\le C {\mathbb {E}}^\prime \int _0^t \Vert S(\bar{{\mathbf {v}}}_{m_k}(s))-S({\mathbf {v}}(s)) \Vert ^2_{{\mathcal {T}}_2(\mathrm {K}_1,\mathrm {H})} ds \end{aligned}$$

3.86 $$\begin{aligned}&\quad + C{\mathbb {E}}^\prime \int _0^t \Vert S({\mathbf {v}}(s))-S^\varepsilon ({\mathbf {v}}(s)) \Vert ^2_{{\mathcal {T}}_2(\mathrm {K}_1,\mathrm {H})} ds. \end{aligned}$$

From Assumption 2.2 and (3.51) we derive that the first term in the right hand side of the last estimate converges to 0 as $m_k\rightarrow \infty$. Owing to (3.83) and (3.62) the sequence in the second term of (3.86) is uniformly integrable with respect to the probability measure ${\mathbb {P}}^\prime$. Thus, from (3.84) and the Vitali Convergence Theorem we infer that

$$\begin{aligned} \lim _{\varepsilon \rightarrow 0 }{\mathbb {E}}^\prime \int _0^t \left\| S({\mathbf {v}}(s))-S^\varepsilon ({\mathbf {v}}(s)) \right\| ^2_{{\mathcal {T}}_2(\mathrm {K}_1,\mathrm {H})} ds=0 . \end{aligned}$$

Hence, for any $t\in (0,T]$

3.87 $$\begin{aligned} \lim _{\varepsilon \rightarrow 0}\lim _{k \rightarrow \infty } {\mathbb {E}}^\prime \left\| {\mathcal {M}}_{m_k}(t)-{\mathcal {M}}^\varepsilon _{m_k}(t)\right\| ^2=0. \end{aligned}$$

In a similar way, we can prove that

3.88 $$\begin{aligned} \lim _{\varepsilon \rightarrow 0}\lim _{k \rightarrow \infty } {\mathbb {E}}^\prime \left\| {\mathcal {M}}^2 (t)-{\mathcal {M}}^\varepsilon (t)\right\| ^2=0. \end{aligned}$$

Next, we will prove that

3.89 $$\begin{aligned} \lim _{\varepsilon \rightarrow 0}\lim _{k \rightarrow \infty } {\mathbb {E}}^\prime \left\| {\mathcal {M}}^\varepsilon _{m_k} (t)-{\mathcal {M}}^\varepsilon (t)\right\| ^2=0. \end{aligned}$$

To this end, we first observe that

3.90 $$\begin{aligned} \begin{aligned} {\mathcal {M}}^\varepsilon _{m_k} (t)-{\mathcal {M}}^\varepsilon (t)&= \int _0^t S^\varepsilon _{m_k}(\bar{{\mathbf {v}}}_{m_k}(s)) {\bar{W}}_{1}^{m_k}(s)- \int _0^t S^\varepsilon _{m_k}({\mathbf {v}}(s)) d{\bar{W}}_{1}(s) \\&\quad + \int _0^t S^\varepsilon _{m_k}({\mathbf {v}}(s)) d{\bar{W}}_{1}(s) -\int _0^t S^\varepsilon ({\mathbf {v}}(s)) d{\bar{W}}_{1}(s)\\&= I^{\varepsilon }_{m_k,1} (t)+ I^\varepsilon _{m_k,2}(t). \end{aligned} \end{aligned}$$

Second, by integration by parts we derive that

$$\begin{aligned} \begin{aligned} I^{\varepsilon }_{m_k,1}(t)&= \int _0^t \left[ \eta _\varepsilon ^\prime \star S_{m_k}({\mathbf {v}}(\cdot )) \right] (s) {\bar{W}}_{1}(s) ds-\int _0^t \left[ \eta _\varepsilon ^\prime \star S_{m_k}(\bar{{\mathbf {v}}}_{m_k}(\cdot ))\right] (s) {\bar{W}}_{1}^{m_k}(s) ds\\&= \int _0^t \left[ \eta _\varepsilon ^\prime \star S_{m_k}(\bar{{\mathbf {v}}}_{m_k}(\cdot ))\right] (s)\left[ {\bar{W}}_{1}^{m_k}(s)-{\bar{W}}_{1}(s)\right] ds \\&\quad + \int _0^t \left[ S^\varepsilon _{m_k}(\bar{{\mathbf {v}}}_{m_k}(s))-S^\varepsilon _{m_k}({\mathbf {v}}(s))\right] d{\bar{W}}_{1}(s)\\&= J^\varepsilon _{m_k,1} (t)+ J^\varepsilon _{m_k,2}(t). \end{aligned} \end{aligned}$$

On one hand, by Proposition 3.25 the processes ${\bar{W}}_{1}^{m_k}$ and ${\bar{W}}_{1}$ are both $\mathrm {K}_1$-cylindrical $\mathrm {K}_2$-valued Wiener processes, thus, for any integer $p\ge 4$ there exists a constant $C>0$ such that

$$\begin{aligned} \sup _{m_k\in {\mathbb {N}}} {\mathbb {E}}^\prime \sup _{s \in [0,T]} \left( \Vert {\bar{W}}_{1}^{m_k}(s)\Vert ^p_{\mathrm {K}_2} + \Vert {\bar{W}}_{1}(s) \Vert ^p_{\mathrm {K}_2}\right) \le C Q T^\frac{p}{2}. \end{aligned}$$

Hence, the sequence $\int _0^t \Vert {\bar{W}}_{1}^{m_k}(s) -{\bar{W}}_{1}(s)\Vert ^2_{\mathrm {K}_2} ds$ is uniformly integrable with respect to the probability measure ${\mathbb {P}}^\prime$, and from (3.42) and the Vitali Convergence Theorem we infer that

3.91 $$\begin{aligned} \lim _{m_k\rightarrow \infty } {\mathbb {E}}^\prime \int _0^t \Vert {\bar{W}}_{1}^{m_k}(s) -{\bar{W}}_{1}(s)\Vert ^2_{\mathrm {K}_2} ds=0. \end{aligned}$$

On the other hand, for any $\varepsilon >0$ there exists a constant $C(\varepsilon )$ such that

$$\begin{aligned} \begin{aligned} {\mathbb {E}}^\prime \int _0^t \left\| \left[ \eta ^\prime _\varepsilon \star S_{m_k}(\bar{{\mathbf {v}}}_{m_k}(\cdot ))\right] (s)\right\| ^2_{{\mathcal {T}}_2(\mathrm {K}_1,\mathrm {H})} ds \le C(\varepsilon ) T {\mathbb {E}}^\prime \sup _{t\in [0,T]} \left\| S_{m_k}(\bar{{\mathbf {v}}}_{m_k}(t)) \right\| ^2_{{\mathcal {T}}_2(\mathrm {K}_1,\mathrm {H})}, \end{aligned} \end{aligned}$$

from which along with Assumption 2.2 and (3.44) we infer that for any $\varepsilon >0$ there exists a constant $C>0$ such that for any $m_k\in {\mathbb {N}}$ we have

$$\begin{aligned} \begin{aligned} {\mathbb {E}}^\prime \int _0^t \left\| \left[ \eta ^\prime _\varepsilon \star S_{m_k}(\bar{{\mathbf {v}}}_{m_k}(\cdot ))\right] (s)\right\| ^2_{{\mathcal {T}}_2\left( \mathrm {K}_1,\mathrm {H}\right) } ds \le C(\varepsilon ) T . \end{aligned} \end{aligned}$$

Thus, from these two observation along with (3.91) we derive that

$$\begin{aligned} \lim _{\varepsilon \rightarrow 0}\lim _{k \rightarrow \infty } {\mathbb {E}}^\prime \left\| J^\varepsilon _{m_k,1} (t) \right\| ^2 = 0, t\in (0, T]. \end{aligned}$$

Using the same argument as in the proof of (3.87) and (3.88) we easily show that

$$\begin{aligned} \lim _{\varepsilon \rightarrow 0}\lim _{k \rightarrow \infty }\left( {\mathbb {E}}^\prime \Vert J^\varepsilon _{m_k,2} (t) \Vert ^2 + {\mathbb {E}}^\prime \Vert I^\varepsilon _{m_k,2}(t) \Vert ^2 \right) =0. \end{aligned}$$

Hence, we have just established that

3.92 $$\begin{aligned} { \lim _{\varepsilon \rightarrow 0}\lim _{k \rightarrow \infty }} {\mathbb {E}}^\prime \Vert I^\varepsilon _{m_k,1}(t) \Vert ^2+ \Vert I^\varepsilon _{m_k,2}(t) \Vert ^2 =0, t \in (0,T], \end{aligned}$$

which along with (3.90) implies that

3.93 $$\begin{aligned} \lim _{\varepsilon \rightarrow 0}\lim _{k \rightarrow \infty } {\mathbb {E}}^\prime \Vert {\mathcal {M}}^\varepsilon _{m_k} (t)-{\mathcal {M}}^\varepsilon (t) \Vert ^2=0. \end{aligned}$$

The identities (3.87), (3.88) and (3.93) imply that for any $t\in (0,T]$

3.94 $$\begin{aligned} \lim _{k \rightarrow \infty }{\mathbb {E}}^\prime \Vert {\mathcal {M}}^1_{m_k}(t) -{\mathcal {M}}^1(t) \Vert ^2=0. \end{aligned}$$

To conclude the proof of the proposition we need to show that ${\mathbb {P}}^\prime$-a.s.

3.95 $$\begin{aligned} {\mathcal {M}}^1_{m_k}(t) -\int _0^t S_{m_k}(\bar{{\mathbf {v}}}_{m_k}(s))d{\bar{W}}_{1}^{m_k}(s)=0, \end{aligned}$$

for any $t\in (0,T]$. To this end, let ${\mathcal {M}}^1_m$ and ${\mathcal {M}}^\varepsilon _m$ be the analogue of ${\mathcal {M}}^1_{m_k}$ and ${\mathcal {M}}^\varepsilon _{m_k}$ with $m_k$ and $\bar{{\mathbf {v}}}_{m_k}$ replaced by *m* and $\bar{{\mathbf {v}}}_m$, respectively. For any ${\mathbf {u}}\in L^2(0,T; \mathrm {V}^{*})$ we set

$$\begin{aligned} \varphi ({\mathbf {u}}) =\frac{\int _0^T \Vert {\mathbf {u}}(s) \Vert ^2_{\mathrm {V}^*} ds }{1+ \int _0^T \Vert {\mathbf {u}}(s) \Vert ^2_{\mathrm {V}^*}ds }. \end{aligned}$$

Since $(\bar{{\mathbf {v}}}_{m_k}, \bar{{\mathbf {n}}}_{m_k}, {\bar{W}}_{1}^{m_k})$ and $(\bar{{\mathbf {v}}}_m, \bar{{\mathbf {n}}}_m, W_1)$ have the same law and $\varphi (\cdot )$ is continuous as a mapping from ${\mathfrak {S}}_1\times {\mathfrak {S}}_2\times C([0,T];\mathrm {K}_1)$ into ${\mathbb {R}}$, we infer that

$$\begin{aligned} {\mathbb {E}} \varphi \left( {\mathcal {M}}^1_m - {\mathcal {M}}^\varepsilon _m\right) ={\mathbb {E}}^\prime \varphi \left( {\mathcal {M}}^1_{m_k} - {\mathcal {M}}^\varepsilon _{m_k}\right) . \end{aligned}$$

Note that arguing as above we can show that as $\varepsilon \rightarrow 0$ we have

$$\begin{aligned} {\mathbb {E}} \varphi \left( {\mathcal {M}}^1_m - {\mathfrak {M}}^1_m\right) ={\mathbb {E}}^\prime \varphi \left( {\mathcal {M}}^1_{m_k} - {\mathfrak {M}}^1_{m_k}\right) , \end{aligned}$$

where

$$\begin{aligned} {\mathfrak {M}}^1_m(\cdot ) =\int _0^{\cdot } S_m(\bar{{\mathbf {v}}}_m(s))dW_1(s). \end{aligned}$$

Since $\bar{{\mathbf {v}}}_m$ and $\bar{{\mathbf {n}}}_m$ are the solution of the Galerkin approximation, we have ${\mathbb {P}}$-a.s. $\varphi ({\mathcal {M}}^1_m - {\mathfrak {M}}^1_m)=0$, from which we infer that

$$\begin{aligned} {\mathbb {E}}^\prime \varphi \left( {\mathcal {M}}^1_{m_k} - {\mathcal {M}}_{m_k}\right) =0. \end{aligned}$$

This last identity implies that $${\mathbb {P}}^\prime $$-a.s. $$ {\mathcal {M}}^1_{m_k}(t) - {\mathcal {M}}_{m_k}(t)=0 $$ for almost all $$t\in (0,T]$$. Since the mappings $$ {\mathcal {M}}^1_{m_k}(\cdot )$$$$ {\mathcal {M}}_{m_k}(\cdot )$$ are continuous in $$\mathrm {V}^*$$ and agree for almost everywhere $$t \in (0,T]$$, necessarily they agree for all $$t\in (0,T]$$. Thus, we have proved the identity (3.95) which along with (3.94) implies the desired equality (3.75). $$\square $$

Now we give the promised proof of the existence of a weak martingale solution.

#### Proof of Theorem 3.2

Endowing the complete probability space $$(\Omega ^\prime , {\mathcal {F}}^\prime , {\mathbb {P}}^\prime )$$ with the filtration $${\mathbb {F}}^\prime =({\mathcal {F}}_t^\prime )_{t\ge 0}$$ which satisfies the usual condition, and combining Propositions 3.24, 3.25 and 3.26 we have just constructed a complete filtered probability space and stochastic processes $${\mathbf {v}}(t), {\mathbf {n}}(t),$$$${\bar{W}}_{1}(t), {\bar{W}}_{2}(t)$$ which satisfy all the items of Definition 3.1. $$\square $$

### Proof of the pathwise uniqueness of the weak solution in the 2-D case

This subsection is devoted to the proof of the uniqueness stated in Theorem 3.4. Before proceeding to the actual proof of this pathwise uniqueness, we state and prove the following lemma.

#### Lemma 3.27

For any $\alpha _8>0$ and $\alpha _9>0$ there exist $C(\alpha _8)>0$, $C_1(\alpha _9)>0$ and $C_2(\alpha _9)>0$ such that

3.96 $$\begin{aligned}&|\langle f({\mathbf {n}}_1)-f({\mathbf {n}}_2), {\mathbf {n}}_1-{\mathbf {n}}_2\rangle |\le \alpha _8 \Vert \nabla {\mathbf {n}}_1-\nabla {\mathbf {n}}_2\Vert ^2 + C(\alpha _8) \Vert {\mathbf {n}}_1-{\mathbf {n}}_2\Vert ^2 \varphi ({\mathbf {n}}_1,{\mathbf {n}}_2), \end{aligned}$$

3.97 $$\begin{aligned}&|\langle f({\mathbf {n}}_1)-f({\mathbf {n}}_2), \mathrm {A}_1{\mathbf {n}}_1-\mathrm {A}_1{\mathbf {n}}_2\rangle |\le \alpha _9 \Vert \mathrm {A}_1{\mathbf {n}}_1-\mathrm {A}_1{\mathbf {n}}_2\Vert ^2\nonumber \\&\quad + C_1(\alpha _9) \Vert \nabla {\mathbf {n}}_1-\nabla {\mathbf {n}}_2\Vert ^2 \varphi ({\mathbf {n}}_1,{\mathbf {n}}_2) \nonumber \\&\quad +C_2(\alpha _9) \Vert {\mathbf {n}}_1-{\mathbf {n}}_2\Vert ^2 \varphi ({\mathbf {n}}_1,{\mathbf {n}}_2), \end{aligned}$$

where

$$\begin{aligned} \varphi ({\mathbf {n}}_1,{\mathbf {n}}_2):= C\left( 1+ \Vert {\mathbf {n}}_1\Vert ^{2N}_{{\mathbf {L}}^{4N+2}} +\Vert {\mathbf {n}}_2\Vert ^{2N}_{{\mathbf {L}}^{4N+2}}\right) ^2. \end{aligned}$$

#### Proof of Lemma 3.27

It is enough to prove the estimate (3.96) for the special case $f({\mathbf {n}}):=a_N |{\mathbf {n}}|^{2N} {\mathbf {n}}$. For this purpose we recall that

$$\begin{aligned} |{\mathbf {n}}_1|^{2N}{\mathbf {n}}_1{-}|{\mathbf {n}}_2|^{2N}{\mathbf {n}}_2{=} \vert {\mathbf {n}}_1\vert ^{2N}({\mathbf {n}}_1{-}{\mathbf {n}}_2)+ {\mathbf {n}}_2(\vert {\mathbf {n}}_1\vert {-}\vert {\mathbf {n}}_2\vert )\left( \sum _{k=0}^{2N-1}\vert {\mathbf {n}}_1\vert ^{2N-k-1}\vert {\mathbf {n}}_2|^{k} \right) , \end{aligned}$$

from which we easily deduce that

$$\begin{aligned} |\langle f({\mathbf {n}}_1)-f({\mathbf {n}}_2), {\mathbf {n}}_1-{\mathbf {n}}_2\rangle |\le C \int _{\mathcal {O}}\left( 1+\vert {\mathbf {n}}_1\vert ^{2N}+\vert {\mathbf {n}}_2\vert ^{2N} \right) |{\mathbf {n}}_1-{\mathbf {n}}_2|^2 dx, \end{aligned}$$

for any ${\mathbf {n}}_1, {\mathbf {n}}_2\in {\mathbb {L}}^{2N+2}({\mathcal {O}})$. Now, invoking the Hölder, Gagliardo–Nirenberg and Young inequalities we infer that

$$\begin{aligned}&|\langle f({\mathbf {n}}_1)-f({\mathbf {n}}_2), {\mathbf {n}}_1-{\mathbf {n}}_2\rangle |\le C \Vert {\mathbf {n}}_1-{\mathbf {n}}_2\Vert _{{\mathbf {L}}^4}^2\left( 1+ \Vert {\mathbf {n}}_1\Vert _{{\mathbf {L}}^{4N+2}}^{2N}+||{\mathbf {n}}_2\Vert _{{\mathbf {L}}^{4N+2}}^{2N} \right) \\&\quad \le C \Vert {\mathbf {n}}_1-{\mathbf {n}}_2\Vert \Vert \nabla \left( {\mathbf {n}}_1-{\mathbf {n}}_2\right) \Vert \left( 1+ \Vert {\mathbf {n}}_1\Vert _{{\mathbf {L}}^{4N+2}}^{2N}+||{\mathbf {n}}_2\Vert _{{\mathbf {L}}^{4N+2}}^{2N} \right) \\&\quad \le \alpha _8 \Vert \nabla \left( {\mathbf {n}}_1-{\mathbf {n}}_2\right) \Vert ^2 +C(\alpha _8)\Vert {\mathbf {n}}_1-{\mathbf {n}}_2 \Vert ^2 \left( 1+ \Vert {\mathbf {n}}_1\Vert _{{\mathbf {L}}^{4N+2}}^{2N}+||{\mathbf {n}}_2\Vert _{{\mathbf {L}}^{4N+2}}^{2N}\right) ^2. \end{aligned}$$

The last line of the above chain of inequalities implies (3.96).

Using the fact that ${\mathbf {H}}^1\subset {\mathbf {L}}^{4N+2}$ for any $N\in {\mathbb {N}}$ and the same argument as in the proof of (3.96) we derive that

$$\begin{aligned}&|\langle f({\mathbf {n}}_1)-f({\mathbf {n}}_2), \mathrm {A}_1{\mathbf {n}}_1-\mathrm {A}_1{\mathbf {n}}_2\rangle |\\&\quad \le C \int _{\mathcal {O}}\left( 1+\vert {\mathbf {n}}_1\vert ^{2N}+\vert {\mathbf {n}}_2\vert ^{2N} \right) |{\mathbf {n}}_1-{\mathbf {n}}_2|\vert \mathrm {A}_1\left( {\mathbf {n}}_1-{\mathbf {n}}_2\right) \vert dx\\&\quad \le C \Vert {\mathbf {n}}_1-{\mathbf {n}}_2\Vert _{{\mathbf {L}}^{4N+2}}\Vert \mathrm {A}_1[{\mathbf {n}}_1-{\mathbf {n}}_2]\Vert \left( 1+ \Vert {\mathbf {n}}_1\Vert _{{\mathbf {L}}^{4N+2}}^{2N}+||{\mathbf {n}}_2\Vert _{{\mathbf {L}}^{4N+2}}^{2N} \right) \\&\quad \le C \Vert {\mathbf {n}}_1-{\mathbf {n}}_2\Vert _{{\mathbf {H}}^1} \Vert \mathrm {A}_1\left[ {\mathbf {n}}_1-{\mathbf {n}}_2\right] \Vert \left( 1+ \Vert {\mathbf {n}}_1\Vert _{{\mathbf {L}}^{4N+2}}^{2N}+||{\mathbf {n}}_2\Vert _{{\mathbf {L}}^{4N+2}}^{2N} \right) \\&\quad \le \alpha _9 \Vert \mathrm {A}_1\left[ {\mathbf {n}}_1-{\mathbf {n}}_2\right] \Vert ^2 +C(\alpha _9)\Vert {\mathbf {n}}_1-{\mathbf {n}}_2 \Vert _{{\mathbf {H}}^1}^2 \left( 1+ \Vert {\mathbf {n}}_1\Vert _{{\mathbf {L}}^{4N+2}}^{2N}+||{\mathbf {n}}_2\Vert _{{\mathbf {L}}^{4N+2}}^{2N} \right) ^2. \end{aligned}$$

From the last line we easily deduce the proof of (3.97). $\square$

Now, we give the promised proof of the uniqueness of our solution.

#### Proof of Theorem 3.4

Let ${\mathbf {v}}={\mathbf {v}}_1-{\mathbf {v}}_2$ and ${\mathbf {n}}={\mathbf {n}}_1-{\mathbf {n}}_2$. These processes satisfy $({\mathbf {v}}(0),{\mathbf {n}}(0))=(0,0)$ and the stochastic equations

$$\begin{aligned} \begin{aligned}&d{\mathbf {v}}(t){+}\biggl (\mathrm {A}{\mathbf {v}}(t){+}B({\mathbf {v}}(t),{\mathbf {v}}_1(t)){+}B({\mathbf {v}}_2(t),{\mathbf {v}}(t))\biggr )dt\\&\quad =-\biggl (M({\mathbf {n}}(t),{\mathbf {n}}_1(t))+M({\mathbf {n}}_2,{\mathbf {n}})\biggr )dt\\&\qquad +[S({\mathbf {v}}_1(t))-S_2({\mathbf {v}}_2(t))]dW_1(t),\\ \end{aligned} \end{aligned}$$

and

$$\begin{aligned} \begin{aligned}&d{\mathbf {n}}(t){+}\biggl (\mathrm {A}_1{\mathbf {n}}(t){+}{\tilde{B}}({\mathbf {v}}(t),{\mathbf {n}}_1(t)){+}{\tilde{B}}({\mathbf {v}}_2(t),{\mathbf {n}}(t))\biggr )dt= -[f({\mathbf {n}}_2(t)){-}f({\mathbf {n}}_1(t))]dt \\&\quad +\frac{1}{2} G^2({\mathbf {n}}(t))dt+G({\mathbf {n}}(t))dW_2(t). \end{aligned} \end{aligned}$$

Firstly, from Young’s inequality and (6.8) we infer that for any $\alpha _1>0$ there exists a constant $C(\alpha _1)>0$ such that

$$\begin{aligned} |\langle B({\mathbf {v}},{\mathbf {v}}_1),{\mathbf {v}}\rangle _{\mathrm {V}^*,\mathrm {V}}|\le \alpha _1 \Vert \nabla {\mathbf {v}}\Vert ^2+C(\alpha _1)\Vert {\mathbf {v}}_1\Vert ^2 \Vert \nabla {\mathbf {v}}_1\Vert ^2 \Vert {\mathbf {v}}\Vert ^2. \end{aligned}$$

Secondly, Young’s inequality and (2.9) yield that for any $\alpha _2>0$, $\alpha _3>0$, $\alpha _4>0$ and $\alpha _7>0$ there exist constants $C(\alpha _2,\alpha _3)>0$ and $C(\alpha _7,\alpha _4)>0$ such that

3.98 $$\begin{aligned}&|\langle M\left( {\mathbf {n}}_2,{\mathbf {n}}\right) ,{\mathbf {v}}\rangle _{\mathrm {V}^*,\mathrm {V}}|\le ||\nabla {\mathbf {v}}||||\nabla {\mathbf {n}}_2 ||^\frac{1}{2} \left( ||{\mathbf {n}}_2 ||+ ||\mathrm {A}_1{\mathbf {n}}_2||\right) ^\frac{1}{2} ||\nabla {\mathbf {n}}||^\frac{1}{2} \left( ||{\mathbf {n}}||+ ||\mathrm {A}_1{\mathbf {n}}||\right) ^\frac{1}{2}\nonumber \\&\quad \le \alpha _2 \Vert \nabla {\mathbf {v}}\Vert ^2+\alpha _3 \left( \Vert \mathrm {A}_1{\mathbf {n}}\Vert ^2+\Vert {\mathbf {n}}\Vert ^2\right) +C\left( \alpha _2,\alpha _3\right) \Vert \nabla {\mathbf {n}}_2\Vert ^2 \left( \Vert \mathrm {A}_1{\mathbf {n}}_2\Vert ^2 +\Vert {\mathbf {n}}_2 \Vert ^2\right) \Vert \nabla {\mathbf {n}}\Vert ^2,\nonumber \\&|\langle M\left( {\mathbf {n}},{\mathbf {n}}_1\right) ,{\mathbf {v}}\rangle _{\mathrm {V}^*,\mathrm {V}}|\le \alpha _7 \Vert \nabla {\mathbf {v}}\Vert ^2+\alpha _4 \left( \Vert \mathrm {A}_1{\mathbf {n}}\Vert ^2+\Vert {\mathbf {n}}\Vert ^2\right) \nonumber \\&\quad +C\left( \alpha _7,\alpha _4\right) \Vert \nabla {\mathbf {n}}_1\Vert ^2 \left( \Vert \mathrm {A}_1{\mathbf {n}}_1\Vert ^2+\Vert {\mathbf {n}}_1\Vert ^2\right) \Vert \nabla {\mathbf {n}}\Vert ^2. \end{aligned}$$

Thirdly, from Young’s inequality and (6.9) we derive that for any $\alpha _5>0$ there exists a constant $C(\alpha _5)>0$ such that

3.99 $$\begin{aligned}&|\langle {\tilde{B}}\left( {\mathbf {v}}_2,{\mathbf {n}}\right) , \mathrm {A}_1{\mathbf {n}}\rangle |\le \left( \Vert {\mathbf {n}}\Vert +\Vert \mathrm {A}_1{\mathbf {n}}\Vert \right) ^\frac{3}{2} \Vert {\mathbf {v}}_2 \Vert ^\frac{1}{2} \Vert \nabla {\mathbf {v}}_2 \Vert ^\frac{1}{2} \Vert \nabla {\mathbf {n}}\Vert ^\frac{1}{2}\nonumber \\&\qquad \alpha _5 \left( \Vert \mathrm {A}_1{\mathbf {n}}\Vert ^2+ \Vert {\mathbf {n}}\Vert ^2\right) +C\left( \alpha _5\right) \Vert {\mathbf {v}}_2\Vert ^2 \Vert \nabla {\mathbf {v}}_2\Vert ^2 \Vert \nabla {\mathbf {n}}\Vert ^2. \end{aligned}$$

From Hölder’s inequality, Gagliardo–Nirenberg’s inequality (6.1) and the Sobolev embedding ${\mathbf {H}}^2\subset {\mathbf {L}}^\infty$ we infer that for any $\alpha _6>0$ there exists $C(\alpha _6)>0$ such that

$$\begin{aligned} |\langle {\tilde{B}}({\mathbf {v}},{\mathbf {n}}_1),{\mathbf {n}}\rangle |&\le \Vert {\mathbf {v}}\Vert \Vert \nabla {\mathbf {n}}_1\Vert \Vert {\mathbf {n}}\Vert _{{\mathbf {L}}^\infty },\\&\le \alpha _6 \left( \Vert {\mathbf {n}}\Vert ^2+ \Vert \mathrm {A}_1{\mathbf {n}}\Vert ^2\right) + C(\alpha _6) \Vert {\mathbf {v}}\Vert ^2\Vert \nabla {\mathbf {n}}_1\Vert ^2. \end{aligned}$$

From the proof of Proposition 3.9 we see that there exists a constant $C>0$ which depends only on $\Vert {\mathbf {h}}\Vert _{{\mathbb {W}}^{1,3}}$ and $\Vert {\mathbf {h}}\Vert _{{\mathbb {L}}^\infty }$ such that

$$\begin{aligned} \Vert \nabla G\left( {\mathbf {n}}\right) \Vert ^2&\le C\left( \Vert \nabla {\mathbf {n}}\Vert ^2+\Vert {\mathbf {n}}\Vert ^2\right) ,\\ \Vert \nabla G^2\left( {\mathbf {n}}\right) \Vert ^2&\le C\left( \Vert \nabla {\mathbf {n}}\Vert ^2+\Vert {\mathbf {n}}\Vert ^2\right) ,\\ |\langle \nabla G^2\left( {\mathbf {n}}\right) , \nabla {\mathbf {n}}\rangle |&\le C\left( \Vert \nabla {\mathbf {n}}\Vert ^2+\Vert {\mathbf {n}}\Vert ^2\right) . \end{aligned}$$

Owing to the Lipschitz property of *S* we have

3.100 $$\begin{aligned} \Vert S({\mathbf {v}}_1)-S({\mathbf {v}}_2)\Vert ^2_{{\mathcal {T}}_2(\mathrm {K}_1, \mathrm {H})}\le C \Vert {\mathbf {v}}\Vert ^2. \end{aligned}$$

Now, let $\varphi ({\mathbf {n}}_1,{\mathbf {n}}_2)$ be as in Lemma 3.27 and

$$\begin{aligned} \Psi (t)=e^{-\int _0^t (\psi _1(s)+\psi _2(s)+\psi _3(s) )ds}, \text { for any } t>0, \end{aligned}$$

where

$$\begin{aligned} \psi _1(s)&:= C(\alpha _1)\Vert {\mathbf {v}}_1(s)\Vert ^2 \Vert \nabla {\mathbf {v}}_1(s)\Vert ^2+ C(\alpha _6)\Vert \nabla {\mathbf {n}}_1(s) \Vert ^2,\\ \psi _3(s)&:= [C(\alpha _8) +C_2(\alpha _9) ] \varphi ({\mathbf {n}}_1(s),{\mathbf {n}}_2(s)), \end{aligned}$$

and

$$\begin{aligned} \begin{aligned} \psi _2(s)&:=C(\alpha _2,\alpha _3)\Vert \nabla {\mathbf {n}}_2(s)\Vert ^2(\Vert {\mathbf {n}}_2(s) \Vert ^2+\Vert \mathrm {A}_1{\mathbf {n}}_2(s)\Vert ^2)\\&\quad +\,C(\alpha _7,\alpha _4)\Vert \nabla {\mathbf {n}}_1(s)\Vert ^2(\Vert {\mathbf {n}}_1(s)\Vert ^2+ \Vert \mathrm {A}_1{\mathbf {n}}_1(s) \Vert ^2)\\&\quad +\,C(\alpha _5) \Vert {\mathbf {v}}_2(s)\Vert ^2\Vert \nabla {\mathbf {v}}_2(s)\Vert ^2 +C_1(\alpha _9) \varphi ({\mathbf {n}}_1(s),{\mathbf {n}}_2(s)). \end{aligned} \end{aligned}$$

Now applying Itô’s formula to $\Vert {\mathbf {n}}(t)\Vert ^2$ and $\Psi (t)\Vert {\mathbf {n}}(t)\Vert ^2$ yield

$$\begin{aligned} \begin{aligned}&d\left[ \Psi (t)\Vert {\mathbf {n}}(t)\Vert ^2\right] =-2\Psi (t)\Vert \nabla {\mathbf {n}}(t)\Vert ^2dt-2 \Psi (t) \langle {\tilde{B}}({\mathbf {v}}(t),{\mathbf {n}}_1(t)),{\mathbf {n}}(t)\rangle \\&\qquad -2 \langle f({\mathbf {n}}_2(t))-f({\mathbf {n}}_2(t)),{\mathbf {n}}(t)\rangle dt+\Psi ^\prime (t) \Vert {\mathbf {n}}(t)\Vert ^2. \end{aligned} \end{aligned}$$

Using the same argument we can show that $\Psi (t)\Vert \nabla {\mathbf {n}}(t)\Vert ^2$ and $\Psi (t)\Vert {\mathbf {v}}(t)\Vert ^2$ satisfy

$$\begin{aligned} \begin{aligned}&d\left[ \Psi (t)\Vert \nabla {\mathbf {n}}(t)\Vert ^2\right] =\Psi (t)\biggl ( -\Vert \mathrm {A}_1{\mathbf {n}}(t)\Vert ^2+\langle {\tilde{B}}({\mathbf {v}}(t),{\mathbf {n}}_1(t))+{\tilde{B}}({\mathbf {v}}_2(t),{\mathbf {n}}(t)), \mathrm {A}_1{\mathbf {n}}(t)\rangle \biggl )dt\\&\quad +\,\Psi (t)\biggl (2 \langle f({\mathbf {n}}_2(t))-f({\mathbf {n}}_1(t)),\mathrm {A}_1{\mathbf {n}}(t)\rangle +\langle \nabla G^2({\mathbf {n}}(t)),\nabla {\mathbf {n}}(t)\rangle \biggr )dt\\&\quad +\,\Vert G({\mathbf {n}}(t))\Vert ^2 dt+ \Psi ^\prime (t)\Vert \nabla {\mathbf {n}}(t)\Vert ^2 dt +2\Psi (t)\langle \nabla G({\mathbf {n}}(t)),\nabla {\mathbf {n}}(t)\rangle dW_2(t), \end{aligned} \end{aligned}$$

and

$$\begin{aligned} \begin{aligned}&d[\Psi (t)\Vert {\mathbf {v}}(t)\Vert ^2]=-2\Psi (t)\biggl (\Vert \nabla {\mathbf {v}}(t)\Vert ^2 +\langle B({\mathbf {v}}(t),{\mathbf {v}}_1(t))+M({\mathbf {n}}(t),{\mathbf {n}}_1(t)),{\mathbf {v}}(t)\rangle _{\mathrm {V}^*,\mathrm {V}}\biggr )dt\\&\quad -2\Psi (t)M({\mathbf {n}}_2(t),{\mathbf {n}}(t)),{\mathbf {v}}(t)\rangle _{\mathrm {V}^*,\mathrm {V}} dt+\Psi (t)\Vert S({\mathbf {v}}_1(t))-S({\mathbf {v}}_2(t))\Vert ^2_{{\mathcal {T}}_2} dt\\&\quad +\Psi ^\prime (t) \Vert {\mathbf {v}}(t)\Vert ^2 dt \\&\quad +2\Psi (t)\langle {\mathbf {v}}(t),[S({\mathbf {v}}_1(t))-S({\mathbf {v}}_2(t))]dW_1(t)\rangle . \end{aligned} \end{aligned}$$

Summing up these last three equalities side by side and using the inequalities (3.97)–(3.100) imply

$$\begin{aligned} \begin{aligned}&d\left[ \Psi (t)\left( \Vert {\mathbf {v}}(t)\Vert ^2+\Vert {\mathbf {n}}(t)\Vert ^2+\Vert \nabla {\mathbf {n}}(t)\Vert ^2\right) \right] \\&\qquad +2\Psi (t)\left[ \Vert \nabla {\mathbf {v}}(t)\Vert ^2+\Vert \nabla {\mathbf {n}}(t)\Vert ^2+\Vert \mathrm {A}_1{\mathbf {n}}(t)\Vert ^2\right] dt\\&\quad \le 2\Psi (t)\left( C \left[ \Vert {\mathbf {v}}(t)\Vert ^2+\Vert {\mathbf {n}}(t)\Vert ^2+\Vert \nabla {\mathbf {n}}(t)\Vert ^2 \right] dt+\langle \nabla G({\mathbf {n}}(t)),\nabla {\mathbf {n}}(t)\rangle dW_2(t)\right) \\&\qquad +2\Psi (t)\left( \langle {\mathbf {v}}(t),\left[ S({\mathbf {v}}_1(t))-S({\mathbf {v}}_2(t))\right] dW_1(t)\rangle +\left[ \alpha _9+ \sum _{j=3}^6 \alpha _j\right] \Vert \mathrm {A}_1{\mathbf {n}}(t)\Vert ^2\right) \\&\qquad +\Psi (t)\left[ \psi _2(t) \Vert \nabla {\mathbf {n}}(t)\Vert ^2 +\psi _1(t) \Vert {\mathbf {v}}(t)\Vert ^2 + \psi _3(t) \Vert {\mathbf {n}}(t)\Vert ^2 \right] dt\\&\qquad +\Psi ^\prime (t)\left( \Vert {\mathbf {v}}(t)\Vert ^2+\Vert {\mathbf {n}}(t)\Vert ^2+\Vert \nabla {\mathbf {n}}(t)\Vert ^2\right) dt\\&\qquad +(\alpha _1+\alpha _2+\alpha _7)\Vert \nabla {\mathbf {v}}(t)\Vert ^2+\alpha _8 \Vert \nabla {\mathbf {n}}(t)\Vert ^2 dt. \end{aligned} \end{aligned}$$

Notice that by the choice of $\Psi$ we have

$$\begin{aligned} \begin{aligned}&\Psi (t)\Big [\psi _2(t) \Vert \nabla {\mathbf {n}}(t)\Vert ^2 +\psi _1(t) \Vert {\mathbf {v}}(t)\Vert ^2 + \psi _3(t) \Vert {\mathbf {n}}(t)\Vert ^2 \Big ] \\&\quad +\Psi ^\prime (t)\biggl (\Vert {\mathbf {v}}(t)\Vert ^2+\Vert {\mathbf {n}}(t)\Vert ^2+\Vert \nabla {\mathbf {n}}(t)\Vert ^2\biggr ) \le 0. \end{aligned} \end{aligned}$$

Hence by choosing $$\alpha _j=\alpha _9=\frac{1}{10}$$, $$j=3,\ldots ,6,$$$$\alpha _i=\alpha _7=\frac{1}{6}, i=2,3$$ and $$\alpha _8=\frac{1}{2}$$ we see that

$$\begin{aligned} \begin{aligned}&d[\Psi (t)\left( \Vert {\mathbf {v}}(t)\Vert ^2+\Vert {\mathbf {n}}(t)\Vert ^2+\Vert \nabla {\mathbf {n}}(t)\Vert ^2\right) ]+\Psi (t)\Big [\Vert \nabla {\mathbf {v}}(t)\Vert ^2+\Vert \mathrm {A}_1{\mathbf {n}}(t)\Vert ^2+\Vert \nabla {\mathbf {n}}(t)\Vert ^2\Big ]dt\\&\quad \le 2\Psi (t)\biggl (C \biggl [\Vert {\mathbf {v}}(t)\Vert ^2+\Vert {\mathbf {n}}(t)\Vert ^2+\Vert \nabla {\mathbf {n}}(t)\Vert ^2 \biggr ]dt+\langle \nabla G({\mathbf {n}}(t)),\nabla {\mathbf {n}}(t)\rangle dW_2(t)\\&\qquad +\langle {\mathbf {v}}(t),[S({\mathbf {v}}_1(t))-S({\mathbf {v}}_2(t))]dW_1(t) \rangle \biggr ). \end{aligned} \end{aligned}$$

Next, integrating and taking the mathematical expectation yield

$$\begin{aligned} \begin{aligned}&{\mathbb {E}}\biggl [\Psi (t)\left( \Vert {\mathbf {v}}(t)\Vert ^2+\Vert {\mathbf {n}}(t)\Vert ^2+\Vert \nabla {\mathbf {n}}(t)\Vert ^2\right) \biggr ]\\&\qquad +{\mathbb {E}} \int _0^t \Psi (s)\Big [\Vert \nabla {\mathbf {v}}(s)\Vert ^2+\Vert \mathrm {A}_1{\mathbf {n}}(s)\Vert ^2+2\Vert \nabla {\mathbf {n}}(s)\Vert ^2\Big ]ds\\&\quad \le C \int _0^t {\mathbb {E}}\biggl [\Psi (s)\left( \Vert {\mathbf {v}}(s)\Vert ^2+\Vert {\mathbf {n}}(s)\Vert ^2+\Vert \nabla {\mathbf {n}}(s)\Vert ^2 \right) \biggr ]ds, \end{aligned} \end{aligned}$$

from which along with Gronwall’s inequality we infer that for any $t\in [0,T]$

$$\begin{aligned} {\mathbb {E}}\left( \Psi (t) \Vert {\mathbf {v}}(t)\Vert ^2 + \Vert {\mathbf {n}}(t)\Vert ^2 + \Vert \nabla {\mathbf {n}}(t)\Vert ^2\right) =0. \end{aligned}$$

$$\square $$

## Uniform estimates for the approximate solutions

This section is devoted to the crucial uniform estimates stated in Propositions 3.8 and 3.9.

### Proof of Proposition 3.8

Let us note that for the sake of simplicity we write $\tau _m$ instead of $\tau _{R,m}$. Let $\Psi (\cdot )$ be the mapping defined by $\Psi ({\mathbf {n}})=\frac{1}{2} ||{\mathbf {n}}||^{p}$ for any ${\mathbf {n}}\in {\mathbf {L}}^2$. This mapping is twice Fréchet differentiable with first and second derivatives defined by

$$\begin{aligned} \Psi ^\prime ({\mathbf {n}})[{\mathbf {g}}]&=p \Vert {\mathbf {n}}\Vert ^{p-2} \langle {\mathbf {n}}, {\mathbf {g}}\rangle ,\\ \Psi ^{\prime \prime }[{\mathbf {g}},{\mathbf {k}}]&=p(p-2)\Vert {\mathbf {n}}\Vert ^{p-4}\langle {\mathbf {n}},{\mathbf {k}}\rangle \langle {\mathbf {n}}, {\mathbf {g}}\rangle +p \Vert {\mathbf {n}}\Vert ^{p-2} \langle {\mathbf {g}}, {\mathbf {k}}\rangle . \end{aligned}$$

By straightforward calculations one can check that if ${\mathbf {g}}\in {\mathbf {L}}^2$ and ${\mathbf {g}}\perp _{{\mathbb {R}}^3} {\mathbf {n}}$ then $\Psi ^\prime ({\mathbf {n}})[{\mathbf {g}}]=0$ and $\Psi ''({\mathbf {n}})[{\mathbf {g}},{\mathbf {g}}]=p \Vert {\mathbf {n}}\Vert ^{p-2}\Vert {\mathbf {g}}\Vert ^2.$

Note that by the self-adjointness of ${\tilde{\pi }}_m$ we have

$$\begin{aligned} \left\langle {\hat{\pi }}_mX_m, {\mathbf {n}}_m\right\rangle =\left\langle X_m, {\mathbf {n}}_m\right\rangle , \end{aligned}$$

where $X_m\in \{G({\mathbf {n}}_m), G^2({\mathbf {n}}_m), {\tilde{B}}({\mathbf {v}}_m,{\mathbf {n}}_m), f({\mathbf {n}}_m)\}$. Thanks to Assumption 2.1 we also have

$$\begin{aligned} {\hat{\pi }}_mf({\mathbf {n}})=f({\mathbf {n}}), \text{ for } \text{ any } {\mathbf {n}}\in {\mathbf {L}}_m. \end{aligned}$$

Since ${\mathbf {v}}_m$ is a divergence free function it follows from lemma 6.1 that

$$\begin{aligned} \langle {\hat{\pi }}_m{\tilde{B}}({\mathbf {v}}_m,{\mathbf {n}}_m),{\mathbf {n}}_m(t)\rangle =\langle {\tilde{B}}({\mathbf {v}}_m,{\mathbf {n}}_m),{\mathbf {n}}_m\rangle =0. \end{aligned}$$

Now, applying Itô’s formula to $\Psi ({\mathbf {n}}_m(t\wedge \tau _m))$ yields

$$\begin{aligned} \begin{aligned}&\Psi ({\mathbf {n}}_m(t\wedge \tau _m))= \Psi ({\mathbf {n}}_{m}(0))-\int _0^{t\wedge \tau _m} \Psi '({\mathbf {n}}_m(s))[A{\mathbf {n}}_m(s)\\&\quad +{\tilde{B}}({\mathbf {v}}_m(s),{\mathbf {n}}_m(s)+ f({\mathbf {n}}_m(s))]\\&\quad +\frac{1}{2} \int _0^{t\wedge \tau _m} \Big (\Psi '({\mathbf {n}}_m(s))[G^2({\mathbf {n}}_m(s))] + \Psi ''({\mathbf {n}}_m(s))[G({\mathbf {n}}_m(s),G({\mathbf {n}}_m(s)]\Big ) ds\\&\quad + \int _0^{t\wedge \tau _m} \Psi '({\mathbf {n}}_m(s))[G({\mathbf {n}}_m(s))] dW_2(s). \end{aligned} \end{aligned}$$

The stochastic integral vanishes because ${\mathbf {n}}_m\times {\mathbf {h}}\perp {\mathbf {n}}_m$ in ${\mathbb {R}}^3$ and

$$\begin{aligned} \langle G({\mathbf {n}}_m),{\mathbf {n}}_m\rangle&= \langle ({\mathbf {n}}_m\times {\mathbf {h}}), {\mathbf {n}}_m\rangle ,\\&= \langle {\mathbf {n}}_m\times {\mathbf {h}}, {\mathbf {n}}_m\rangle ,\\&=0. \end{aligned}$$

Since ${\mathbf {v}}_m$ is a divergence free function it follows from (6.10) that

$$\begin{aligned} \langle {\tilde{B}}({\mathbf {v}}_m,{\mathbf {n}}_m),{\mathbf {n}}_m\rangle =0. \end{aligned}$$

From the identity

$$\begin{aligned} \langle (b\times a)\times a, b\rangle _{{\mathbb {R}}^3,{\mathbb {R}}^3}=-||a\times b||^2_{{\mathbb {R}}^3}, \end{aligned}$$

we infer that

$$\begin{aligned}&\Psi '({\mathbf {n}}_m)[G^2({\mathbf {n}}_m)] + \Psi ''({\mathbf {n}}_m)[G({\mathbf {n}}_m),G({\mathbf {n}}_m)]\\&\quad =2p\Vert {\mathbf {n}}_m\Vert ^{2(p-1)}\Big [\langle G^2({\mathbf {n}}_m), {\mathbf {n}}_m\rangle +||G({\mathbf {n}}_m)||^2\Big ]\\&\quad = 0. \end{aligned}$$

Consequently,

4.1 $$\begin{aligned} \begin{aligned}&||{\mathbf {n}}_m(t\wedge \tau _m)||^{p}=||{\mathbf {n}}_m(0)||^{p}-p \int _0^{t\wedge \tau _m} \Vert {\mathbf {n}}_m(s)\Vert ^{p-2} ||\nabla {\mathbf {n}}_m(s) ||^2 ds\\&\quad -p\int _0^{t\wedge \tau _m} \Vert {\mathbf {n}}_m(s) \Vert ^{p-2} \langle f({\mathbf {n}}_m(s)), {\mathbf {n}}_m(s)\rangle ds. \end{aligned} \end{aligned}$$

Now, by Assumption 2.1 that there exists a polynomial ${\tilde{F}}(r)=\sum _{l=1}^{N+1} b_l r^l$ with ${\tilde{F}}(0)=0$ and $b_{N+1}>0$ such that

$$\begin{aligned} \langle f({\mathbf {n}}_m), {\mathbf {n}}_m\rangle = \int _{{\mathcal {O}}} {\tilde{F}}\left( \vert {\mathbf {n}}_m(x)\vert ^2\right) dx. \end{aligned}$$

In fact, it follows from Assumption 2.1 that

$$\begin{aligned} \left\langle {\tilde{f}}(\vert {\mathbf {n}}_m\vert ^2){\mathbf {n}}_m, {\mathbf {n}}_m\right\rangle&= \int _{{\mathcal {O}}} {\tilde{f}}\left( \vert {\mathbf {n}}_m(x)\vert ^2\right) \vert {\mathbf {n}}_m(x)\vert ^2 dx\\&= \int _{{\mathcal {O}}} \sum _{k=0}^{N} a_k \left( \vert {\mathbf {n}}_m(x)\vert ^2\right) ^{k+1} dx\\&= \int _{{\mathcal {O}}} \sum _{l=1}^{N+1} a_{l-1} \left( \vert {\mathbf {n}}_m(x)\vert ^2\right) ^{l} dx. \end{aligned}$$

Thanks to this observation we can use [8, Lemma 8.7] to infer that there exists $c>0$ such that

$$\begin{aligned} \frac{a_{N+1}}{2} \int _{{\mathcal {O}}} |{\mathbf {n}}_m(x)|^{2N+2} dx-c \int _{{\mathcal {O}}} |{\mathbf {n}}_m(x)|^2 dx \le \langle f({\mathbf {n}}_m), {\mathbf {n}}_m\rangle . \end{aligned}$$

From this estimate and (4.1) we deduce that there exists a constant $C>0$ independent of $m\in {\mathbb {N}}$ such that

4.2 $$\begin{aligned} \begin{aligned}&||{\mathbf {n}}_m(t\wedge \tau _m)||^{p}+p \int _0^{t\wedge \tau _m} \Vert {\mathbf {n}}_m(s)\Vert ^{p-2} ||\nabla {\mathbf {n}}_m(s) ||^2 ds\\&\qquad + p \int _0^{t\wedge \tau _m} \Vert {\mathbf {n}}_m(s) \Vert ^{p-2} \Vert {\mathbf {n}}_m(s) \Vert ^{2N+2}_{{\mathbf {L}}^{2N+2}} ds\\&\quad \le C \int _0^{t\wedge \tau _m} \Vert {\mathbf {n}}_m(s) \Vert ^p ds + \Vert {\mathbf {n}}_m(0)\Vert ^p, \end{aligned} \end{aligned}$$

from which along with the fact that $||{\mathbf {n}}_m(0)||=||{\tilde{\pi }}_m {\mathbf {n}}_0||\le ||{\mathbf {n}}_0||$ and an application of the Gronwall lemma we complete the proof of our proposition. $\square$

### Proof of Proposition 3.9

Let us note that that for the sake of simplicity we write $\tau _m$ instead of $\tau _{R,m}$. By the self-adjointness of $\pi _m$ we have

$$\begin{aligned} \langle {\hat{\pi }}_mY_m, {\mathbf {v}}_m\rangle =\langle Y_m, {\mathbf {v}}_m\rangle , \end{aligned}$$

where $Y_m\in \{B({\mathbf {v}}_m,{\mathbf {v}}_m), M({\mathbf {n}}_m,{\mathbf {n}}_m)\}$. A similar remark holds for those operators involving ${\hat{\pi }}_m$ (see the proof of Proposition 3.8).

Application of Itô’s formula to $\Phi ({\mathbf {v}}_m(t\wedge \tau _m))=\frac{1}{2} ||{\mathbf {v}}_m(t\wedge \tau _m)||^2$, $t\in [0,T)$, yields

4.3 $$\begin{aligned} \begin{aligned}&\frac{1}{2} ||{\mathbf {v}}_m(t\wedge \tau _m) ||^2{-}||\pi _m{\mathbf {v}}_0||^2={-}\int _0^{t\wedge \tau _m }\biggl \langle \mathrm {A}{\mathbf {v}}_m(s){+}B({\mathbf {v}}_m(s)){+}M({\mathbf {n}}_m(s)), {\mathbf {v}}_m\biggr \rangle \, ds\\&\quad +\frac{1}{2} \int _0^{t\wedge \tau _m}||S({\mathbf {v}}_m)||^2_{{\mathcal {T}}_2(\mathrm {K}_1,\mathrm {H})}\, ds +\int _0^{t\wedge \tau _m }\left\langle {\mathbf {v}}_m(s), S({\mathbf {v}}_m(s))dW_1(s)\right\rangle . \end{aligned} \end{aligned}$$

We now introduce the mapping $\Psi$ defined by

$$\begin{aligned} \Psi ({\mathbf {n}})=\frac{1}{2}||\nabla {\mathbf {n}}||^2+\frac{1}{2} \int _{\mathcal {O}}F(\vert {\mathbf {n}}(x) \vert ^2)\,\, dx, {\mathbf {n}}\in {\mathbf {H}}^1. \end{aligned}$$

Thanks to Assumption 2.1 one can apply [8, Lemma 8.10] to infer that the mapping $\Psi (\cdot )$ is twice Fréchet differentiables and its first and second derivatives of $\Psi$ are given by

$$\begin{aligned} \Psi ^\prime ({\mathbf {n}}){\mathbf {g}}&=\langle \nabla {\mathbf {n}}, \nabla {\mathbf {g}}\rangle +\langle f({\mathbf {n}}), {\mathbf {g}}\rangle ,\\ \Psi ^{\prime \prime }({\mathbf {n}})({\mathbf {g}}, {\mathbf {g}})&=\langle \nabla {\mathbf {g}}, \nabla {\mathbf {g}}\rangle +\int _{\mathcal {O}}{\tilde{f}}({\mathbf {n}}) |{\mathbf {g}}|^2 dx +\int _{\mathcal {O}}[{\tilde{f}}^\prime ({\mathbf {n}}) ][{\mathbf {n}}\cdot {\mathbf {g}}]^2\,\, dx, \end{aligned}$$

$\text { for all } {\mathbf {n}}, {\mathbf {g}} \in {\mathbf {H}}^1.$ Observe that if ${\mathbf {g}}\perp {\mathbf {n}}$ in ${\mathbb {R}}^3$, then

$$\begin{aligned} \Psi ^{\prime \prime }({\mathbf {n}})({\mathbf {g}}, {\mathbf {g}})=\langle \nabla {\mathbf {g}}, \nabla {\mathbf {g}}\rangle +\int _{\mathcal {O}}{\tilde{f}}({\mathbf {n}}) |{\mathbf {g}}|^2 dx. \end{aligned}$$

Note also that

$$\begin{aligned} \Psi ^\prime ({\mathbf {n}}){\mathbf {g}}= \langle -\mathrm {A}_1{\mathbf {n}}, {\mathbf {g}}\rangle + \langle f({\mathbf {n}}), {\mathbf {g}}\rangle , \end{aligned}$$

for all ${\mathbf {n}}\in {\mathbf {H}}^2$ and ${\mathbf {g}}\in {\mathbf {H}}^1$. Before proceeding further we should also recall that it was proved in [8, Lemma 8.9] that there exists ${\tilde{\ell }} >0$ such that

4.4 $$\begin{aligned} \Vert \nabla {\mathbf {n}}\Vert ^2+\Vert {\mathbf {n}}\Vert ^2 \le 2 \Psi ({\mathbf {n}})+{\tilde{\ell }} \Vert {\mathbf {n}}\Vert ^2, \end{aligned}$$

for any $${\mathbf {n}}\in {\mathbf {H}}^1$$.

Now, by Itô’s formula we have

$$\begin{aligned} \begin{aligned}&\Psi ({\mathbf {n}}_m(t\wedge \tau _m))-\Psi ({\hat{\pi }}_m{\mathbf {n}}_0)\\&\quad =\int _0^{t\wedge \tau _m}\left( -\Vert \mathrm {A}_1{\mathbf {n}}_m(s) +f({\mathbf {n}}_m(s))\Vert ^2 +\frac{1}{2} \int _{\mathcal {O}}{\tilde{f}}({\mathbf {n}}_m(s))|G({\mathbf {n}}_m(s))|^2\right) ds \\&\qquad +\int _0^{t\wedge \tau _m}\biggl \langle \biggl (\frac{1}{2} G^2({\mathbf {n}}_m(s))-{\tilde{B}}({\mathbf {v}}_m(s),{\mathbf {n}}_m(s))\biggr ),f({\mathbf {n}}_m(s))+\mathrm {A}_1{\mathbf {n}}_m(s)\biggr \rangle ds\\&\qquad +\frac{1}{2} ||\nabla G({\mathbf {n}}_m(s))||^2+ \langle G({\mathbf {n}}_m(s)), f({\mathbf {n}}_m(s))+\mathrm {A}_1{\mathbf {n}}_m(s)\rangle dW_2(s), \end{aligned} \end{aligned}$$

which is equivalent to

$$\begin{aligned} \begin{aligned}&\Psi ({\mathbf {n}}_m(t\wedge \tau _m))-\Psi ({\hat{\pi }}_m{\mathbf {n}}_0)\\&\quad =\int _0^{t\wedge \tau _m} \left( -\Vert \mathrm {A}_1{\mathbf {n}}_m(s) +f({\mathbf {n}}_m(s))\Vert ^2 +\int _{\mathcal {O}}{\tilde{f}}({\mathbf {n}}_m(s))|G({\mathbf {n}}_m(s))|^2\,\, dx \right) ds\\&\qquad +\int _0^{t\wedge \tau _m}\left( \langle \frac{1}{2} G^2({\mathbf {n}}_m(s)), f({\mathbf {n}}_m(s))+\mathrm {A}_1{\mathbf {n}}_m(s)\rangle -\langle {\tilde{B}}({\mathbf {v}}_m(s),{\mathbf {n}}_m(s)),\mathrm {A}_1{\mathbf {n}}_m(s)\rangle \right) ds\\&\qquad +\frac{1}{2} \int _0^{t\wedge \tau _m} ||\nabla G({\mathbf {n}}_m(s))||^2 ds+ \int _0^{t\wedge \tau _m} \langle G({\mathbf {n}}_m(s)), f({\mathbf {n}}_m(s))+\mathrm {A}_1{\mathbf {n}}_m(s)\rangle dW_2(s). \end{aligned} \end{aligned}$$

Here we used the fact that

$$\begin{aligned} \langle {\mathbf {v}}_m\cdot \nabla {\mathbf {n}}_m, f({\mathbf {n}}_m)\rangle&= \sum _{i,j} \int _{\mathcal {O}}{\mathbf {v}}_m^{(i)}(x) \frac{\partial {\mathbf {n}}_m^{(j)}(x)}{\partial x_i} {\tilde{f}}(|{\mathbf {n}}_m(x)|^2) {{\mathbf {n}}_m}^{(j)}(x) dx\\&= \frac{1}{2} \sum _{i=1}^d \int _{\mathcal {O}}{\mathbf {v}}_m^{(i)}(x) \frac{\partial {\tilde{F}}(|{\mathbf {n}}_m(x)|^2)}{\partial x_i} dx\\&= \langle {\mathbf {v}}_m, \nabla {\tilde{F}}(\vert {\mathbf {n}}_m|^2)\rangle \\&= 0, \text { because }\mathrm {div }\;{\mathbf {v}}_m=0. \end{aligned}$$

Now, let us observe that

$$\begin{aligned} \frac{1}{2} \biggl \vert \int _{\mathcal {O}}{\tilde{f}}\left( \vert {\mathbf {n}}_m(x)\vert ^2\right) |G({\mathbf {n}}_m(x))|^2 dx\biggr \vert \le \frac{1}{2} \Vert {\mathbf {h}}\Vert _{{\mathbb {L}}^\infty }^2 \int _{\mathcal {O}}\vert {\tilde{f}}\left( \vert {\mathbf {n}}_m(x)\vert ^2\right) \vert \,\,\vert {\mathbf {n}}_m(x) \vert ^2 dx. \end{aligned}$$

Next, by setting ${\bar{f}}(r)=\sum _{k=0}^N b_k r^k$ with $b_k=\vert a_k\vert$, $k=0,\ldots , N$, we derive that there exists a polynomial ${\tilde{Q}}$ of degree *N* such that

$$\begin{aligned} {\bar{f}}(r)r= a_N r^{N+1} + {\tilde{Q}}(r) . \end{aligned}$$

From this last identity and the former estimate we derive that

$$\begin{aligned}&\frac{1}{2} \biggl \vert \int _{\mathcal {O}}{\tilde{f}}(\vert {\mathbf {n}}_m\vert ^2) |G({\mathbf {n}}_m)|^2 dx\biggr \vert \\&\quad \le \frac{1}{2} \Vert {\mathbf {h}}\Vert _{{\mathbf {L}}^\infty }^2 \biggl [ a_N\int _{\mathcal {O}}\vert {\mathbf {n}}_m(x) \vert ^{2N+2} dx+ \biggl |\int _{{\mathcal {O}}} {\tilde{Q}}(|{\mathbf {n}}_m(x)|^2) dx \biggr |\biggr ], \end{aligned}$$

from which along with [8, Lemma 8.7] we deduce that there exists a constant $C>0$ which depends only on $||{\mathbf {h}}||_{{\mathbf {L}}^\infty }$ such that

$$\begin{aligned} \frac{1}{2} \biggl \vert \int _{\mathcal {O}}{\tilde{f}}(\vert {\mathbf {n}}_m\vert ^2) |G({\mathbf {n}}_m)|^2 dx\biggr \vert \le C\Big ( \int _{\mathcal {O}}F(\vert {\mathbf {n}}_m(x) \vert ^2) dx+ \Vert {\mathbf {n}}_m\Vert ^2 \Big ). \end{aligned}$$

Thus,

$$\begin{aligned} \frac{1}{2} \biggl \vert \int _{\mathcal {O}}{\tilde{f}}(\vert {\mathbf {n}}_m\vert ^2) |G({\mathbf {n}}_m)|^2 dx\biggr \vert \le C\Big ( \Psi ({\mathbf {n}}_m) + \Vert {\mathbf {n}}_m\Vert ^2\Big ), \end{aligned}$$

and

4.5 $$\begin{aligned} \begin{aligned}&\Psi ({\mathbf {n}}_m(t\wedge \tau _m))+ \Psi ({\hat{\pi }}_m{\mathbf {n}}_0)\le \int _0^{t\wedge \tau _m} \left( -\Vert \mathrm {A}_1{\mathbf {n}}_m(s) +f({\mathbf {n}}_m(s))\Vert ^2 \right. \\&\quad \left. +\,C \Big [ \Psi ({\mathbf {n}}_m(s)) + \Vert {\mathbf {n}}_m(s) \Vert ^2\Big ] \right) ds \\&\quad +\int _0^{t\wedge \tau _m}\left( \left\langle \frac{1}{2} G^2({\mathbf {n}}_m(s)), f({\mathbf {n}}_m(s))+\mathrm {A}_1{\mathbf {n}}_m(s)\right\rangle \right. \\&\quad \quad \left. -\langle {\tilde{B}}({\mathbf {v}}_m(s),{\mathbf {n}}_m(s)),\mathrm {A}_1{\mathbf {n}}_m(s)\rangle \right) ds\\&\quad +\frac{1}{2} \int _0^{t\wedge \tau _m} ||\nabla G({\mathbf {n}}_m(s))||^2 ds + \langle G({\mathbf {n}}_m(s)), f({\mathbf {n}}_m(s))+\mathrm {A}_1{\mathbf {n}}_m(s)\rangle dW_2(s). \end{aligned} \end{aligned}$$

Thanks to (2.10) we derive that

$$\begin{aligned} -\langle {\tilde{B}}({\mathbf {v}}_m,{\mathbf {n}}_m), \mathrm {A}_1{\mathbf {n}}_m\rangle -\langle M({\mathbf {n}}_m), {\mathbf {v}}_m\rangle =0. \end{aligned}$$

From Lemma 6.1 we also derive that

$$\begin{aligned} \langle B ({\mathbf {v}}_m,{\mathbf {v}}_m), {\mathbf {v}}_m\rangle =0. \end{aligned}$$

Thus, adding up inequality (4.3) and inequality (4.5) and using the last two identities we see that

4.6 $$\begin{aligned}&\left[ \Psi ({\mathbf {n}}_m)+\frac{1}{2} \Vert {\mathbf {v}}_m\Vert ^2\right] (t\wedge \tau _m)- \left[ \Psi ({\hat{\pi }}_m{\mathbf {n}}_0)+ ||\pi _m {\mathbf {v}}_0 ||_{{\mathbb {L}}^2}\right] \nonumber \\&\quad + \int _0^{t\wedge \tau _m}\left[ \Vert \nabla {\mathbf {v}}_m(s)\Vert ^2 -(\Psi ({\mathbf {n}}_m(s)) + \Vert {\mathbf {n}}_m(s) \Vert ^2)\right] ds \nonumber \\&\quad \le \frac{1}{2}\int _0^{t\wedge \tau _m} \biggl ( - \Vert \mathrm {A}_1{\mathbf {n}}_m(s) +f({\mathbf {n}}_m(s))\Vert ^2+\Vert S({\mathbf {v}}_m(s))\Vert ^2_{{\mathcal {T}}_2} \\&\quad \quad + \Vert G^2({\mathbf {n}}_m(s))\Vert ^2 +||\nabla G({\mathbf {n}}_m(s))\Vert ^2 \biggr )ds\nonumber \\&\quad + \int _0^{t\wedge \tau _m}\langle {\mathbf {v}}_m(s), S({\mathbf {v}}_m(s))dW_1(s)\rangle +\langle G({\mathbf {n}}_m(s)), f({\mathbf {n}}_m(s))+\mathrm {A}_1{\mathbf {n}}_m(s)\rangle dW_2(s).\nonumber \end{aligned}$$

Since $G({\mathbf {n}}_m)={\mathbf {n}}_m\times {\mathbf {h}}$, we have

4.7 $$\begin{aligned} ||\nabla G({\mathbf {n}}_m)||^2&\le ||G({\mathbf {n}}_m)\Vert ^2_{{\mathbf {H}}^1},\nonumber \\&\le \Vert \nabla ({\mathbf {n}}_m\times {\mathbf {h}})\Vert ^2+\Vert {\mathbf {n}}_m\times {\mathbf {h}}\Vert ^2\nonumber \\&\le 2[\Vert \nabla {\mathbf {n}}_m\times {\mathbf {h}}\Vert ^2+ \Vert {\mathbf {n}}_m\times \nabla {\mathbf {h}}\Vert ^2]+\Vert {\mathbf {n}}_m\times {\mathbf {h}}\Vert ^2\nonumber \\&\le C \Vert {\mathbf {h}}\Vert ^2_{{\mathbf {L}}^\infty }(\Vert \nabla {\mathbf {n}}_m\Vert ^2 + \Vert {\mathbf {n}}_m\Vert ^2) +\Vert {\mathbf {n}}_m\times \nabla {\mathbf {h}}\Vert ^2. \end{aligned}$$

From Hölder’s inequality and the Sobolev embedding ${\mathbf {H}}^1\subset {\mathbf {L}}^6$ (true for $d=2,3$!) we obtain

$$\begin{aligned} \Vert {\mathbf {n}}_m\times \nabla {\mathbf {h}}\Vert ^2&\le \Vert {\mathbf {n}}_m\Vert ^2_{{\mathbf {L}}^6}\Vert \nabla {\mathbf {h}}\Vert ^2_{{\mathbf {L}}^3},\\&\le c\left( \Vert \nabla {\mathbf {n}}_m\Vert ^2+\Vert {\mathbf {n}}_m\Vert ^2\right) \Vert \nabla {\mathbf {h}}\Vert ^2_{{\mathbf {L}}^3}. \end{aligned}$$

By plugging this last inequality into (4.7), we infer the existence of a constant $C>0$ which depend only on $||{\mathbf {h}}||_{{\mathbf {W}}^{1,3}}$ such that

$$\begin{aligned} \Vert \nabla G({\mathbf {n}}_m)\Vert ^2 \le C (\Vert \nabla {\mathbf {n}}_m\Vert ^2 +\Vert {\mathbf {n}}_m\Vert ^2). \end{aligned}$$

In a similar way one can prove that there exists $C>0$ which depends only on $\Vert {\mathbf {h}}\Vert _{{\mathbf {L}}^\infty }$ such that

$$\begin{aligned} \Vert G^2({\mathbf {n}}_m)\Vert ^2&\le C \Vert {\mathbf {n}}_m\Vert ^2\le C \left( \Vert \nabla {\mathbf {n}}_m\Vert ^2+\Vert {\mathbf {n}}_m\Vert ^2\right) . \end{aligned}$$

From the last two estimates, (4.4) and the linear growth assumption (2.2) we derive that there exists a constant $C>0$ such that

4.8 $$\begin{aligned}&\frac{1}{2}\biggl (\Vert S({\mathbf {v}}_m)\Vert ^2_{{\mathcal {T}}_2(\mathrm {K}_1, \mathrm {H})} + \Vert G^2({\mathbf {v}}_m)\Vert ^2 +||\nabla G({\mathbf {n}}_m)\Vert ^2 \biggr )\nonumber \\&\quad \le C\Vert {\mathbf {v}}_m\Vert ^2 +2 C\Psi ({\mathbf {n}}_m)+{\tilde{\ell }} C \Vert {\mathbf {n}}_m\Vert ^2. \end{aligned}$$

From this inequality and (4.6) we derive that there exists $C>0$ such that

4.9 $$\begin{aligned}&{\mathbb {E}}\sup _{s\in [0,t\wedge \tau _m]}\left[ \Psi ({\mathbf {n}}_m(s))+\frac{1}{2} \Vert {\mathbf {v}}_m(s))\Vert ^2\right] \nonumber \\&\qquad + {\mathbb {E}}\int _0^{t\wedge \tau _m}\biggl (\Vert \nabla {\mathbf {v}}_m(s))\Vert ^2 + \frac{1}{2} \Vert \mathrm {A}_1{\mathbf {n}}_m(s) +f({\mathbf {n}}_m(s))\Vert ^2\biggr )ds\nonumber \\&\quad \le C {\mathbb {E}}\int _0^{t\wedge \tau _m} \Big [ \Vert {\mathbf {v}}_m(s)\Vert ^2 +\Psi ({\mathbf {n}}_m(s))+{\tilde{\ell }} \Vert {\mathbf {n}}_m(s) \Vert ^2\Big ] ds \\&\qquad + {\mathbb {E}}\sup _{s\in [0,t\wedge \tau _m] }\biggl \vert \int _0^{s\wedge \tau _m} \langle {\mathbf {v}}_m(r), S({\mathbf {v}}_m(r))dW_1(r) \rangle \biggr \vert \nonumber \\&\qquad + {\mathbb {E}}\sup _{s\in [0,t\wedge \tau _m]}\biggl \vert \int _0^{s\wedge \tau _m} \langle G({\mathbf {n}}_m(r)), f({\mathbf {n}}_m(r))+\mathrm {A}_1{\mathbf {n}}_m(r) \rangle dW_2(r)\biggr \vert \nonumber \\&\qquad + {\mathbb {E}}\left( \frac{1}{2} \Vert {\mathbf {v}}_0\Vert ^2+ \Psi ({\mathbf {n}}_m(0))\right) .\nonumber \end{aligned}$$

Thanks to the Burkholder–Davis–Gundy, Cauchy–Schwarz and Cauchy inequalities we infer that

4.10 $$\begin{aligned}&{\mathbb {E}}\sup _{s\in [0,t\wedge \tau _m]}\biggl \vert \int _0^{s\wedge \tau _m} \langle G({\mathbf {n}}_m(r)), f({\mathbf {n}}_m(r))+\mathrm {A}_1{\mathbf {n}}_m(r) \rangle dW_2(r)\biggr \vert \nonumber \\&\quad \le C {\mathbb {E}}\biggl (\int _0^{t\wedge \tau _m} [\langle G({\mathbf {n}}_m(s)),\mathrm {A}_1{\mathbf {n}}_m(s)+f({\mathbf {n}}_m(s))\rangle ]^2 ds\biggr )^\frac{1}{2}\nonumber \\&\quad \le C {\mathbb {E}}\biggl [\sup _{s\in [0,t\wedge \tau _m]}\Vert G({\mathbf {n}}_m(s))\Vert \biggl (\int _0^{t\wedge \tau _m} \Vert \mathrm {A}_1{\mathbf {n}}_m(s)+f({\mathbf {n}}_m(s))\Vert ^2 ds\biggr )^\frac{1}{2} \biggr ]\nonumber \\&\quad \le C {\mathbb {E}}\sup _{s\in [0,t\wedge \tau _m]}\Vert G({\mathbf {n}}_m(s))\Vert ^2 +\frac{1}{4} {\mathbb {E}}\int _0^{t\wedge \tau _m} \Vert \mathrm {A}_1{\mathbf {n}}_m(s)+f({\mathbf {n}}_m(s))\Vert ^2 ds\nonumber \\&\quad \le C \Vert {\mathbf {h}}\Vert ^2_{{\mathbf {L}}^\infty }{\mathbb {E}}\sup _{s\in [0,t\wedge \tau _m]}\Vert {\mathbf {n}}_m(s)\Vert ^2 +\frac{1}{4} {\mathbb {E}}\int _0^{t\wedge \tau _m} \Vert \mathrm {A}_1{\mathbf {n}}_m(s)+f({\mathbf {n}}_m(s))\Vert ^2 ds. \end{aligned}$$

By making use of a similar argument and (2.2) one can prove that

4.11 $$\begin{aligned}&{\mathbb {E}}\sup _{s\in [0,t\wedge \tau _m] }\biggl \vert \int _0^{s\wedge \tau _m} \langle {\mathbf {v}}_m(r), S({\mathbf {v}}_m(r))dW_1(r) \rangle \biggr \vert \le \frac{1}{4}{\mathbb {E}}\sup _{s\in [0,t\wedge \tau _m]}\Vert {\mathbf {v}}_m(s))\Vert ^2 \nonumber \\&\quad +\,C {\mathbb {E}}\int _0^{t\wedge \tau _m} \Vert {\mathbf {v}}_m(s)) \Vert ^2 ds. \end{aligned}$$

Note that from (4.4) we easily derive that $\Vert {\mathbf {v}}_m \Vert ^2 +\Vert {\mathbf {n}}_m\Vert ^2_{{\mathbf {H}}^1} \le \Vert {\mathbf {v}}_m\Vert ^2+ 2\Psi ({\mathbf {n}}_m) +{\tilde{\ell }} \Vert {\mathbf {n}}_m\Vert ^2.$ Hence, using (4.10) and (4.11) in (4.9) we infer that

$$\begin{aligned} \begin{aligned}&{\mathbb {E}}\sup _{s\in \left[ 0,t\wedge \tau _m\right] }\left[ \Psi ({\mathbf {n}}_m(s))+\frac{1}{2} \Vert ({\mathbf {v}}_m(s))\Vert ^2\right] \\&\qquad + {\mathbb {E}}\int _0^{t\wedge \tau _m}\biggl (\Vert \nabla {\mathbf {v}}_m(s))\Vert ^2 + \frac{1}{2} \Vert \mathrm {A}_1{\mathbf {n}}_m(s) +f({\mathbf {n}}_m(s))\Vert ^2\biggr )ds\\&\quad \le C {\mathbb {E}}\int _0^{t\wedge \tau _m} \left( \Vert {\mathbf {v}}_m(s))\Vert ^2 +\Psi ({\mathbf {n}}_m(s))\right) ds + C \varphi (t\wedge \tau _m), \end{aligned} \end{aligned}$$

where $\varphi (\cdot )$ is the non-decreasing function defined by

$$\begin{aligned} \varphi (t)= {\mathbb {E}}\left( \Vert {\mathbf {v}}_0\Vert ^2+\Psi ({\mathbf {n}}_m(0)) \right) +{\mathbb {E}}\sup _{s\in [0,t]} \Vert {\mathbf {n}}_m(s)\Vert ^2+{\mathbb {E}}\int _0^{t} \Vert {\mathbf {n}}_m(s)\Vert ^2 ds. \end{aligned}$$

Now, it follows from Gronwall’s lemma that

$$\begin{aligned} \begin{aligned}&{\mathbb {E}}\sup _{s\in \left[ 0,t\wedge \tau _m\right] }\left[ \Psi ({\mathbf {n}}_m(s))+\frac{1}{2} \Vert ({\mathbf {v}}_m(s))\Vert ^2\right] \\&\qquad + {\mathbb {E}}\int _0^{t\wedge \tau _m}\biggl (\Vert \nabla {\mathbf {v}}_m(s))\Vert ^2 + \frac{1}{2} \Vert \mathrm {A}_1{\mathbf {n}}_m(s) +f({\mathbf {n}}_m(s))\Vert ^2\biggr )ds\\&\quad \le \varphi (t\wedge \tau _m) \left( 1+T e^{C T}\right) , \end{aligned} \end{aligned}$$

which altogether with Proposition 3.8 completes the proof of Proposition 3.9 for the case $$ p=1$$.

For the case $p\ge 4N+2$ we first observe that from (4.6) and (4.8) we easily see that

$$\begin{aligned} \begin{aligned}&\Psi ({\mathbf {n}}_m(t\wedge \tau _m))+{\tilde{\ell }} \Vert {\mathbf {n}}_m(t\wedge \tau _m)\Vert ^2 +\Vert {\mathbf {v}}_m(t\wedge \tau _m) \Vert ^2\\&\qquad +\int _0^{t\wedge \tau _m}\biggl (2 \Vert \nabla {\mathbf {v}}_m(s)) \Vert ^2+ \Vert \mathrm {A}_1{\mathbf {n}}_m(s)+f({\mathbf {n}}_m(s))\Vert ^2 \biggr )ds\\&\quad \le \Psi ({\mathbf {n}}_0)+\Vert {\mathbf {v}}_0\Vert ^2 + {\tilde{\ell }} [||{\mathbf {n}}_0||^2 +\Vert {\mathbf {n}}_m(t\wedge \tau _m)\Vert ^2] \\&\qquad +C \int _0^{t\wedge \tau _m} \left( 2\Psi ({\mathbf {n}}_m(s))+\ell \Vert {\mathbf {n}}_m(s)\Vert ^2 +\Vert {\mathbf {v}}_m(s))\Vert ^2\right) ds \\&\quad \biggl |\int _0^{t\wedge \tau _m} \langle {\mathbf {v}}_m(s), S({\mathbf {v}}_m(s))dW_1(s)\rangle \biggr |\\&\qquad + \biggl |\int _0^{t\wedge \tau _m} \langle G({\mathbf {n}}_m(s)), f({\mathbf {n}}_m(s))+\mathrm {A}_1{\mathbf {n}}_m(s)\rangle dW_2(s)\biggr |. \end{aligned} \end{aligned}$$

Second, rising both sides of this estimate to the power *p* and taking the supremum over $s\in [0,t\wedge \tau _m]$ and the mathematical expectation imply that there exists a constant $C>0$ depending only in *p* such that

4.12 $$\begin{aligned}&{\mathbb {E}}\sup _{s\in [0,t\wedge \tau _m]}[\psi (s)]^p- {\mathbb {E}}[\psi (0)]^p \nonumber \\&\qquad + {\mathbb {E}}\biggl [\int _0^{t\wedge \tau _m} \biggl (2 \Vert \nabla {\mathbf {v}}_m(s)) \Vert ^2+ \Vert \mathrm {A}_1{\mathbf {n}}_m(s)+f({\mathbf {n}}_m(s))\Vert ^2 \biggr )ds\biggr ]^p\nonumber \\&\quad \le C {\mathbb {E}}\sup _{t\in [0,T]}||{\mathbf {n}}_m(t)||^{2p} +C T {\mathbb {E}}\int _0^{t\wedge \tau _m} [\psi (s)]^p ds \\&\qquad + C {\mathbb {E}}\sup _{s\in [0,t\wedge \tau _m]}\biggl |\int _0^{s\wedge \tau _m} \langle {\mathbf {v}}_m(r), S({\mathbf {v}}_m(r))dW_1\rangle \biggr |^p\nonumber \\&\qquad + C {\mathbb {E}}\sup _{s\in [0,t\wedge \tau _m] }\biggl |\int _0^{s\wedge \tau _m} \langle G({\mathbf {n}}_m(r)), f({\mathbf {n}}_m(r))+\mathrm {A}_1{\mathbf {n}}_m(r)\rangle dW_2\biggr |^p,\nonumber \end{aligned}$$

where, for the sake of simplicity, we have put

$$\begin{aligned} \psi (t)=\Psi ({\mathbf {n}}_m(t))+\ell \Vert {\mathbf {n}}_m(t)\Vert ^2 +\Vert {\mathbf {v}}_m(t))\Vert ^2. \end{aligned}$$

Now by making use of the Burkholder–Davis–Gundy, Cauchy–Schwarz, Cauchy inequalities and the linear growth assumption (2.2) we derive that

4.13 $$\begin{aligned}&{\mathbb {E}}\sup _{s\in [0,t\wedge \tau _m]}\biggl |\int _0^{s\wedge \tau _m} \langle {\mathbf {v}}_m(r), S({\mathbf {v}}_m(r))dW_1\rangle \biggr |^p\le C {\mathbb {E}}\biggl (\int _0^{t\wedge \tau _m} \Vert {\mathbf {v}}_m(s))\Vert ^2 \Vert S({\mathbf {v}}_m(s)))\Vert ^2_{{\mathcal {T}}_2} ds\biggr )^\frac{p}{2}\nonumber \\&\quad \le C {\mathbb {E}}\biggl [\sup _{s\in [0,t\wedge \tau _m]} [\psi (s)]^\frac{p}{2}\biggl (\int _0^{t\wedge \tau _m} (1+\Vert {\mathbf {v}}_m(s))\Vert ^2)ds\biggr )^\frac{p}{2} \biggr ]\nonumber \\&\quad \le \frac{1}{4} {\mathbb {E}}\sup _{s\in [0,t\wedge \tau _m]} [\psi (s)]^p+C T +C {\mathbb {E}}\int _0^{t\wedge \tau _m} [\psi (s)]^p ds. \end{aligned}$$

By using a similar argument we obtain

$$\begin{aligned}&{\mathbb {E}}\sup _{s\in [0,t\wedge \tau _m] }\biggl |\int _0^{s\wedge \tau _m} \langle G({\mathbf {n}}_m(r)), f({\mathbf {n}}_m(r))+\mathrm {A}_1{\mathbf {n}}_m(r)\rangle dW_2\biggr |^p\\&\qquad \le C {\mathbb {E}}\biggl (\int _0^{t\wedge \tau _m} \Vert G({\mathbf {n}}_m(s))\Vert ^2 \Vert \mathrm {A}_1{\mathbf {n}}_m(s)+f({\mathbf {n}}_m(s))\Vert ^2 ds\biggr )^\frac{p}{2}\\&\qquad \le C {\mathbb {E}}\sup _{s\in [0,t\wedge \tau _m]}\Vert {\mathbf {n}}_m(s)\times {\mathbf {h}}\Vert ^p + \frac{1}{2} {\mathbb {E}}\biggl (\int _0^{t\wedge \tau _m} \Vert \mathrm {A}_1{\mathbf {n}}_m(s)+f({\mathbf {n}}_m(s))\Vert ^2 ds \biggr )^p. \end{aligned}$$

From the last line we easily derive that

4.14 $$\begin{aligned} \begin{aligned}&{\mathbb {E}}\sup _{s\in [0,t\wedge \tau _m] }\biggl |\int _0^{s\wedge \tau _m} \langle G({\mathbf {n}}_m(r)), f({\mathbf {n}}_m(r))+\mathrm {A}_1{\mathbf {n}}_m(r)\rangle dW_2\biggr |^p-C {\mathbb {E}}\sup _{s\in [0,t\wedge \tau _m]} \Vert {\mathbf {n}}_m(s)\Vert ^{2p}\\&\quad \le \frac{1}{2} {\mathbb {E}}\biggl (\int _0^{t\wedge \tau _m} \left( 2 \Vert \nabla {\mathbf {v}}_m(s))\Vert ^2+\Vert \mathrm {A}_1{\mathbf {n}}_m(s)+f({\mathbf {n}}_m(s))\Vert ^2\right) ds \biggr )^p. \end{aligned} \end{aligned}$$

Plugging (4.13) and (4.14) in (4.12) yields that

$$\begin{aligned} \begin{aligned}&{\mathbb {E}}\sup _{s\in [0,t\wedge \tau _m]}[\psi (s)]^p+ {\mathbb {E}}\biggl [\int _0^{t\wedge \tau _m} 2 \Vert \nabla {\mathbf {v}}_m(s)) \Vert ^2+ \Vert \mathrm {A}_1{\mathbf {n}}_m(s)+f({\mathbf {n}}_m(s))\Vert ^2 \biggr )ds\biggr ]\\&\quad \le 2 {\mathbb {E}}[\psi (0)]^p+ C T +C {\mathbb {E}}\sup _{s\in [0,t\wedge \tau _m]} \Vert {\mathbf {n}}_m(s)\Vert ^{2p}+ C(t\wedge \tau _m+1){\mathbb {E}}\int _0^{t\wedge \tau _m} [\psi (s)]^p ds, \end{aligned} \end{aligned}$$

from which altogether with the Gronwall lemma implies that

$$\begin{aligned} \begin{aligned}&{\mathbb {E}}\sup _{s\in [0,t\wedge \tau _m]}[\psi (s)]^p+ {\mathbb {E}}\biggl [\int _0^{t\wedge \tau _m} 2 \Vert \nabla {\mathbf {v}}_m(s)) \Vert ^2+ \Vert \mathrm {A}_1{\mathbf {n}}_m(s)+f({\mathbf {n}}_m(s))\Vert ^2 \biggr )ds\biggr ]\\&\quad \le \biggl [2 {\mathbb {E}}[\psi (0)]^p+ C T+C {\mathbb {E}}\sup _{s\in [0,t\wedge \tau _m]} \Vert {\mathbf {n}}_m(s)\Vert ^{2p} \biggr ]\left( 1+ CT(T+1) e^{C(T+1)T}\right) . \end{aligned} \end{aligned}$$

This along with (3.20) complete the proof of the proposition. $\square$

## Maximum principle type theorem

In this section we replace in the system (3.1)–(3.3) the general polynomial $f({\mathbf {n}})$ by the bounded Ginzburg–Landau function $\mathbb {1}_{|{\mathbf {n}}|\le 1}(|{\mathbf {n}}|^2-1){\mathbf {n}}$. All our previous result remains true and the analysis are even easier. In the case $f({\mathbf {n}})=\mathbb {1}_{|{\mathbf {n}}|\le 1}(|{\mathbf {n}}|^2-1){\mathbf {n}}$, we will show that if the initial value ${\mathbf {n}}_0$ is in the unit ball, then so are the values of the vector director ${\mathbf {n}}$. That is, we must show that $|{\mathbf {n}}(t)|^2 \le 1$ almost all $(\omega , t,x)\in \Omega \times [0,T]\times {\mathcal {O}}$. In fact we have the following theorem.

### Theorem 5.1

Assume that $d \le 3$ and that a process $({\mathbf {v}},{\mathbf {n}})=({\mathbf {v}}(t), {\mathbf {n}}(t))_{t\in [0,T]}$, is a solution to problem (3.1)–(3.3) with initial condition $({\mathbf {v}}_0,{\mathbf {n}}_0)$ such that $|{\mathbf {n}}_0|^2\le 1$ for almost all $(\omega ,x)\in \Omega \times {\mathcal {O}}$. Then $|{\mathbf {n}}(t)|^2 \le 1$ for almost all $(\omega , t,x)\in \Omega \times [0,T]\times {\mathcal {O}}$.

### Proof

We follow the idea in [11, Lemma 2.1] and [16, Proof of Theorem 4, Page 513]. Let $\varphi :{\mathbb {R}}\rightarrow [0,1]$ be an increasing function of class ${\mathcal {C}}^\infty$ such that

$$\begin{aligned} \varphi (s) = 0 \text { iff } s\in (-\infty , 1],\\ \varphi (s)=1 \text { iff } s\in [2,+\infty ). \end{aligned}$$

Let $\{{\tilde{\varphi }}_m: m\in {\mathbb {N}}\}$ and $\{{\tilde{\phi }}_m: m\in {\mathbb {N}}\}$ be two sequences of function ${\mathbb {R}}$ defined by

5.1 $$\begin{aligned} {\tilde{\varphi }}_m(a)&=\varphi (m a),\;\; a \in {\mathbb {R}}, \end{aligned}$$

5.2 $$\begin{aligned} {\tilde{\phi }}_m(a)&=a^2 {\varphi (m a)}, \;\; a \in {\mathbb {R}}. \end{aligned}$$

We also set

5.3 $$\begin{aligned} \varphi _m({\mathbf {u}})&={\tilde{\varphi }}_m (|{\mathbf {u}}|^2-1) ,\; {\mathbf {u}}\in {\mathbb {R}}^3, \end{aligned}$$

5.4 $$\begin{aligned} \phi _m({\mathbf {u}})&={\tilde{\phi }}_m(|{\mathbf {u}}|^2-1), \;{\mathbf {u}}\in {\mathbb {R}}^3. \end{aligned}$$

For each $m \in {\mathbb {N}}$ let $\Psi _m: {\mathbf {H}}^{2 }\rightarrow {\mathbb {R}}$ be a function defined by

5.5 $$\begin{aligned} \Psi _m({\mathbf {u}})&=\Vert \phi _m \circ {\mathbf {u}}\Vert _{{\mathbf {L}}^1}, \nonumber \\&=\int _{\mathcal {O}}\left( |{\mathbf {u}}(x)|^2-1\right) ^2 [\varphi _m({\mathbf {u}}(x))] dx,\,\, {\mathbf {u}}\in {\mathbf {H}}^{2}. \end{aligned}$$

The mapping $\Psi _m$ is twice (Fréchet) differentiable and its first and second derivatives satisfy

5.6 $$\begin{aligned} \begin{aligned} \Psi _m^\prime ({\mathbf {u}})({\mathbf {k}} )&= 4\int _{\mathcal {O}}\left( |{\mathbf {u}}(x)|^2-1)\varphi _m({\mathbf {u}}(x)) [{\mathbf {u}}(x)\cdot {\mathbf {k}}(x)] \right) dx\\&\quad + 2m \int _{\mathcal {O}}\left( |{\mathbf {u}}(x)|^2-1\right) ^2 \varphi ^\prime \left( m \left( |{\mathbf {u}}(x)|^2-1\right) \right) ({\mathbf {u}}(x)\cdot {\mathbf {k}}(x))dx , \;\;\\&\quad \text {for }{\mathbf {u}}\in {\mathbf {H}}^{2} , {\mathbf {k}}\in {\mathbf {L}}^2 , \end{aligned} \end{aligned}$$

and,

$$\begin{aligned} \begin{aligned}&\Psi ^{\prime \prime }_m({\mathbf {u}})({\mathbf {k}},{\mathbf {f}})= 4m^2 \int _{\mathcal {O}}\left[ (|{\mathbf {u}}(x)|^2-1)^2 \varphi _m^{\prime \prime }(m(|{\mathbf {u}}(x)|^2-1 )) ({\mathbf {u}}(x)\cdot {\mathbf {k}}(x) ) ({\mathbf {u}}(x) \cdot {\mathbf {f}}(x) ) \right] dx\\&\quad +16m \int _{\mathcal {O}}\left[ (|{\mathbf {u}}(x)|^2-1)\varphi ^\prime (m(|{\mathbf {u}}(x)|^2 -1)) ({\mathbf {u}}(x) \cdot {\mathbf {k}}(x) ) ({\mathbf {u}}(x) \cdot {\mathbf {f}}(x) )\right] dx\\&\quad + 8\int _{\mathcal {O}}\biggl [\varphi _m({\mathbf {u}}(x)) ({\mathbf {u}}(x)\cdot {\mathbf {k}}(x) ) ({\mathbf {u}}(x) \cdot {\mathbf {f}}(x)) \biggr ]dx\\&\quad + 2m \int _{\mathcal {O}}\left[ |{\mathbf {u}}(x)|^2 -1)^2 \varphi ^\prime (m (|{\mathbf {u}}(x) |^2-1) ) ({\mathbf {k}}(x)\cdot {\mathbf {f}}(x) \right] dx\\&\quad +4 \int _{\mathcal {O}}\left[ \varphi _m({\mathbf {u}}(x)) (|{\mathbf {u}}(x)|^2-1) ({\mathbf {k}}(x)\cdot {\mathbf {f}}(x) )\right] dx, \end{aligned} \end{aligned}$$

for ${\mathbf {u}}\in {\mathbf {H}}^{2}$ and ${\mathbf {k}},{\mathbf {f}} \in {\mathbf {L}}^2$. In particular, if ${\mathbf {u}}\in {\mathbf {H}}^{2}$ and ${\mathbf {k}},{\mathbf {f}} \in {\mathbf {L}}^2$ are such that

$$\begin{aligned} {\mathbf {k}}(x)\perp {\mathbf {u}}(x) \text{ and } {\mathbf {f}}(x)\perp {\mathbf {u}}(x) \text{ for } \text{ all } x \in {\mathcal {O}}, \end{aligned}$$

then

5.7 $$\begin{aligned} \Psi _m^\prime ({\mathbf {u}})({\mathbf {k}})=0, \end{aligned}$$

and

5.8 $$\begin{aligned} \begin{aligned} \Psi _m^{\prime \prime }({\mathbf {u}})({\mathbf {k}},{\mathbf {f}} )&= 4\int _{\mathcal {O}}\left[ (|{\mathbf {u}}(x) |^2-1)\varphi _m({\mathbf {u}}(x)) ({\mathbf {k}}(x) \cdot {\mathbf {f}}(x) )\right] dx\\&\quad +2m \int _{\mathcal {O}}\left[ (|{\mathbf {u}}(x) |^2-1)^2 \varphi ^\prime ( m (|{\mathbf {u}}(x) |^2 -1) ) ( {\mathbf {k}}(x) \cdot {\mathbf {f}}(x)) \right] dx. \end{aligned} \end{aligned}$$

It follows from Itô’s formula (see [37, Theorem I.3.3.2, Page 147]) that

$$\begin{aligned} \begin{aligned} d[\Psi _m({\mathbf {n}})]&=\Psi _m ({\mathbf {n}}) \left( -\mathrm {A}_1 {\mathbf {n}}-{\tilde{B}}({\mathbf {v}},{\mathbf {n}})-\frac{1}{\varepsilon ^2}f({\mathbf {n}})+\frac{1}{2} G^2({\mathbf {n}})\right) dt\\&\quad +\frac{1}{2} \Psi _m^{\prime \prime }({\mathbf {n}}) (G({\mathbf {n}}), G({\mathbf {n}})) dt. \end{aligned} \end{aligned}$$

The stochastic integral vanishes because $G({\mathbf {n}})\perp _{{\mathbb {R}}^3} {\mathbf {n}}$. Since $G^2({\mathbf {n}})=({\mathbf {n}}\times {\mathbf {h}})\times {\mathbf {h}}$ and $G({\mathbf {n}})={\mathbf {n}}\times {\mathbf {h}}$, we infer from (5.6) and the identity

$$\begin{aligned} -|a \times b|^2_{{\mathbb {R}}^3}=a\cdot \left( (a\times b)\times b\right) , a, b \in {\mathbb {R}}^3, \end{aligned}$$

that

$$\begin{aligned} \begin{aligned} \Psi ^\prime ({\mathbf {n}})(G^2({\mathbf {n}}))&= -2m \int _{\mathcal {O}}(|{\mathbf {n}}(x) |^2-1 ) \varphi ^\prime (m(|{\mathbf {n}}(x)|^2-1 ) ) |G({\mathbf {n}}(x))|^2 dx\\&\quad -4 \int _{\mathcal {O}}(|{\mathbf {n}}(x) |^2-1)\varphi _m ({\mathbf {n}}(x))|G({\mathbf {n}}(x))|^2 dx, \end{aligned} \end{aligned}$$

which along with the fact that $G({\mathbf {n}}(x)) \perp {\mathbf {n}}(x)$ for any $x \in {\mathcal {O}}$ and (5.8) we infer that

$$\begin{aligned} \frac{1}{2} \Psi _m^{\prime \prime }(G({\mathbf {n}}), G({\mathbf {n}}))+\frac{1}{2} \Psi _m^{\prime }(G^2({\mathbf {n}}))=0. \end{aligned}$$

Hence

5.9 $$\begin{aligned} d[\Psi _m({\mathbf {n}})]=\Psi _m ({\mathbf {n}}) \left( -\mathrm {A}_1 {\mathbf {n}}-{\tilde{B}}({\mathbf {v}},{\mathbf {n}})-\frac{1}{\varepsilon ^2}f({\mathbf {n}})\right) dt. \end{aligned}$$

Now, observe that from the assumptions on $\varphi$ and the definition of ${\tilde{\phi }}_m, m \in {\mathbb {N}}$ we can show that for any $a\in {\mathbb {R}}$,

5.10 $$\begin{aligned} {\tilde{\phi }}_m(a) \rightarrow (a_+)^2 \text { and } m \varphi ^\prime (m a) \rightarrow 0 \text { as } m \rightarrow \infty , \end{aligned}$$

where $a_{+}:=\max (a,0)$. Observe also that there exists a constant $C>0$ such that for all $m \in {\mathbb {N}}$ and $a\in {\mathbb {R}}$

5.11 $$\begin{aligned} |{\tilde{\phi }}_m(a)|\le C a^2 \text { and } |m \varphi ^\prime (m a)|\le C \vert a |. \end{aligned}$$

We now easily infer from (5.10), (5.11) and the Lebesgue Dominated Convergence Theorem that for ${\mathbf {u}}\in {\mathbf {H}}^{2}, {\mathbf {k}}\in {\mathbf {L}}^2$

$$\begin{aligned} \lim _{m \rightarrow \infty }\Psi _m({\mathbf {u}})&=\left\Vert\left( |{\mathbf {u}}|^2-1\right) _{-}\right\Vert^2,\\ \lim _{m \rightarrow \infty }\Psi ^\prime _m({\mathbf {u}})({\mathbf {k}})&=4 \int _{\mathcal {O}}\left[ \left( |{\mathbf {u}}(x)|^2-1\right) _{-}({\mathbf {u}}(x)\cdot {\mathbf {k}}(x))\right] \,dx. \end{aligned}$$

Hence, setting $y(t)=\Vert \left( |{\mathbf {n}}(t)|^2-1\right) _{+}\Vert ^2$ we obtain from letting $\ell \rightarrow \infty$ in (5.9) that for almost all $(\omega ,t)\in \Omega \times [0,T]$

$$\begin{aligned} y(t)-y(0)+4\int _0^t \biggl (\int _{\mathcal {O}}\biggl [\mathrm {A}_1 {\mathbf {n}}+({\mathbf {v}}\cdot \nabla ){\mathbf {n}}+\frac{1}{\varepsilon ^2} f({\mathbf {n}}) \biggr ]\cdot \biggl [{\mathbf {n}}\left( |{\mathbf {n}}|^2-1\right) _{+}\biggr ] dx \biggr )ds=0. \end{aligned}$$

Let us set $\xi =\left( |{\mathbf {n}}|^2-1\right) _{+}$, it follows from [3, Exercise 7.1.5, p 283] that $\xi \in H^1$ if ${\mathbf {n}}\in {\mathbf {H}}^1$. Thus, since $\frac{\partial {\mathbf {n}}}{\partial \varvec{\nu }}=0$ we derive from integration-by-parts that

$$\begin{aligned} 4 \int _0^t \biggl (\int _{\mathcal {O}}\mathrm {A}_1{\mathbf {n}}\cdot {\mathbf {n}}\left( |{\mathbf {n}}|^2{-}1\right) _{+} dx \biggr )ds{=} \int _0^t \biggl (\int _{\mathcal {O}}\left( 2 \nabla (|{\mathbf {n}}|^2)\cdot \nabla \xi {+}4 \xi |\nabla {\mathbf {n}}|^2 \right) dx \biggr ) ds. \end{aligned}$$

Since $\xi \ge 0$ and $|\nabla {\mathbf {n}}|^2\ge 0$ a.e. $(t,x)\in {\mathcal {O}}\times [0,T]$ we easily derive from the above identity that

$$\begin{aligned} 4 \int _0^t \biggl (\int _{\mathcal {O}}\mathrm {A}_1{\mathbf {n}}\cdot {\mathbf {n}}\left( |{\mathbf {n}}|^2-1\right) _{+} dx \biggr )ds \ge 2 \int _0^t \biggl (\int _{\mathcal {O}}\nabla (|{\mathbf {n}}|^2-1)\cdot \nabla \xi dx \biggr )ds. \end{aligned}$$

Thanks to [3, Exercise 7.1.5, p 283] we have

$$\begin{aligned} \int _0^t \biggl (\int _{\mathcal {O}}\nabla (|{\mathbf {n}}|^2-1)\cdot \nabla \xi dx \biggr )ds= \int _0^t \int _{\mathcal {O}}|\nabla (|{\mathbf {n}}|^2-1) |^2 \mathbb {1}_{\{|{\mathbf {n}}|^2>1\}}dx \,\, ds, \end{aligned}$$

which implies

$$\begin{aligned} 4 \int _0^t \biggl (\int _{\mathcal {O}}\mathrm {A}_1{\mathbf {n}}\cdot {\mathbf {n}}\left( |{\mathbf {n}}|^2-1\right) _{+} dx \biggr )ds\ge \int _0^t \int _{\mathcal {O}}|\nabla (|{\mathbf {n}}|^2-1) |^2 \mathbb {1}_{\{|{\mathbf {n}}|^2>1\}}dx \,\, ds. \end{aligned}$$

We also have

$$\begin{aligned}&4\int _0^t \biggl (\int _{\mathcal {O}}[({\mathbf {v}}\cdot \nabla ){\mathbf {n}}]\cdot [{\mathbf {n}}\left( |{\mathbf {n}}|^2-1\right) _{+}] dx \biggr )ds\\&\quad =2\int _0^t \biggl (\int _{\mathcal {O}}[({\mathbf {v}}\cdot \nabla )(|{\mathbf {n}}|^2) ][ \left( |{\mathbf {n}}|^2-1\right) _{+}] dx \biggr )ds\\&\quad =\int _0^t \biggl (\int _{\mathcal {O}}({\mathbf {v}}\cdot \nabla ) \xi dx \biggr )ds\\&\quad =0. \end{aligned}$$

Since $f({\mathbf {n}})=0$ for $|{\mathbf {n}}|^2>1$ and $\xi f({\mathbf {n}})=0$ for $|{\mathbf {n}}|^2 \le 1$ we have

$$\begin{aligned} 4\int _0^t \biggl (\int _{\mathcal {O}}\xi f({\mathbf {n}}) \cdot {\mathbf {n}}\,dx \biggr ) ds=0. \end{aligned}$$

Therefore we see that *y*(*t*) satisfies the estimate

$$\begin{aligned} y(t)+2\int _0^t \int _{\{|{\mathbf {n}}|^2>1\}}|\nabla (|{\mathbf {n}}|^2-1)_{+}|^2 ds \le y(0), \end{aligned}$$

for almost all $(\omega ,t)\in \Omega \times [0,T]$. Since the second term in the left hand side of the above inequality is positive and $y(0)=\Vert (|{\mathbf {n}}_0|^2-1)_{+}\Vert ^2$ and by assumption $|{\mathbf {n}}_0|^2\le 1$ for almost all $(\omega ,t,x)\in \Omega \times [0,T]\times {\mathcal {O}}$ we derive that

$$\begin{aligned} y(t)=0, \end{aligned}$$

for almost all $(\omega , t)\in \Omega \times [0,T]$, $T\ge 0$. Hence we have $|{\mathbf {n}}|^2 \le 1$ a.e. $(\omega , t,x)\in \Omega \times [0,T]\times {\mathcal {O}}$, $T\ge 0$. $\square$

## 6. Appendix: Some important estimates

In this section we recall or establish some crucial estimates needed for the proof of our mains results.

First, let $d\in [1,4]$ and put $a=\frac{d}{4}$. Then the following estimates, valid for all ${\mathbf {u}}\in {\mathbb {W}}^{1,4}$, are special cases of Gagliardo–Nirenberg’s inequalities:

6.1 $$\begin{aligned}&\Vert {\mathbf {u}}\Vert _{{\mathbb {L}}^4}\le \Vert {\mathbf {u}}\Vert ^{1-a} \Vert \nabla {\mathbf {u}}\Vert ^a, \end{aligned}$$

6.2 $$\begin{aligned}&\Vert {\mathbf {u}}\Vert _{{\mathbb {L}}^\infty }\le \Vert {\mathbf {u}}\Vert _{{\mathbb {L}}^4}^{1-a}\Vert \nabla {\mathbf {u}}\Vert _{{\mathbb {L}}^4}^a. \end{aligned}$$

The inequality (6.1) can be written in the spirit of the continuous embedding

6.3 $$\begin{aligned} {\mathbb {H}}^1\subset {\mathbb {L}}^4. \end{aligned}$$

It follows from (6.2) and (6.3) that for ${\mathbf {u}}\in {\mathbb {H}}^2$

6.4 $$\begin{aligned} \Vert {\mathbf {u}}\Vert _{{\mathbb {L}}^\infty }\le \Vert {\mathbf {u}}\Vert _1 ^{1-a}\Vert {\mathbf {u}}\Vert _{2}^a. \end{aligned}$$

All these facts hold as well for the corresponding spaces ${\mathbf {L}}^r$, $r=4, \infty$, and ${\mathbf {H}}^\ell$, $\ell =1,2$. Next we give some properties of the bilinear form *B* and ${\tilde{B}}$ defined in Sect. 2 (see Eqs. (2.3) and (2.4) on page 6, respectively).

### Lemma 6.1

The bilinear mapping $B(\cdot , \cdot )$ mappings continuously $\mathrm {V}\times {\mathbb {H}}^1$ into $\mathrm {V}^*$ and

6.5 $$\begin{aligned}&\langle B({\mathbf {u}},{\mathbf {v}}),{\mathbf {w}}\rangle _{\mathrm {V}^*,\mathrm {V}}=b({\mathbf {u}},{\mathbf {v}},{\mathbf {w}}), \text { for any } {\mathbf {u}}\in \mathrm {V}, {\mathbf {v}}\in {\mathbb {H}}^1,{\mathbf {w}}\in \mathrm {V}, \end{aligned}$$

6.6 $$\begin{aligned}&\langle B({\mathbf {u}},{\mathbf {v}}),{\mathbf {w}}\rangle _{\mathrm {V}^*,\mathrm {V}}=-b({\mathbf {u}},{\mathbf {w}},{\mathbf {v}}) \text { for any } {\mathbf {u}}\in \mathrm {V}, {\mathbf {v}}\in {\mathbb {H}}^1,{\mathbf {w}}\in \mathrm {V}, \end{aligned}$$

6.7 $$\begin{aligned}&\langle B({\mathbf {u}},{\mathbf {v}}),{\mathbf {v}}\rangle _{\mathrm {V}^*,\mathrm {V}}=0 \,\, \text { for any } {\mathbf {u}}\in \mathrm {V}, {\mathbf {v}}\in \mathrm {V}, \end{aligned}$$

6.8 $$\begin{aligned}&\Vert B({\mathbf {u}},{\mathbf {v}})\Vert _{\mathrm {V}^*}\le C_0 \Vert {\mathbf {u}}\Vert ^{1-\frac{d}{4}} \Vert \nabla {\mathbf {u}}\Vert ^{\frac{d}{4}} \Vert {\mathbf {v}}\Vert ^{1-\frac{d}{4}}\Vert \nabla {\mathbf {v}}\Vert ^\frac{d}{4}, \text { for all } {\mathbf {u}}\in \mathrm {V}, {\mathbf {v}}\in {\mathbb {H}}^1. \end{aligned}$$

### Proof

This lemma is well-known and we refer to [45, Chapter II, Section 1.2] for its proof. $\square$

With an abuse of notation, we again denote by ${\tilde{B}}(\cdot ,\cdot )$ the restriction of ${\tilde{B}}(\cdot ,\cdot )$ to $\mathrm {V}\times {\mathbf {H}}^2$.

### Lemma 6.2

The bilinear operator ${\tilde{B}}$ mappings continuously $\mathrm {V}\times {\mathbf {H}}^2$ into ${\mathbf {L}}^2$ and there exists $C_1>0$ such that

6.9 $$\begin{aligned} \Vert {\tilde{B}} ({\mathbf {v}}, {\mathbf {n}})\Vert \le C_1 \Vert {\mathbf {v}}\Vert ^{1-\frac{d}{4}}\Vert \nabla {\mathbf {v}}\Vert ^\frac{d}{4} \Vert \nabla {\mathbf {n}}\Vert ^{1-\frac{d}{4}} \Vert \nabla ^2 {\mathbf {n}}\Vert ^{\frac{d}{4}}, \text { for any } {\mathbf {v}}\in \mathrm {V}, {\mathbf {n}}\in {\mathbf {H}}^2.\qquad \end{aligned}$$

Moreover, we have

6.10 $$\begin{aligned} \langle {\tilde{B}}({\mathbf {v}},{\mathbf {n}}),{\mathbf {n}}\rangle&=0,\text { for any } {\mathbf {v}}\in \mathrm {V}, {\mathbf {n}}\in {\mathbf {H}}^2. \end{aligned}$$

### Proof

We can argue as in the proof of (6.8) (see also [45, Chapter II, Section 1.2]) to establish the estimate (6.9). The identity (6.10) easily follows by integration-by-parts and by taking into account that ${{\,\mathrm{div}\,}}{\mathbf {v}}=0$ and ${\mathbf {v}}$ is zero on the boundary. $\square$

### Remark 6.3

Using the same arguments as in the proof of Lemma 6.2 we can also prove that $B(\cdot , \cdot )$ mappings continuously $\mathrm {V}\times D(\mathrm {A})$ into $\mathrm {H}$. Furthermore, *B* satisfies (6.9) with $({\mathbf {v}},{\mathbf {n}})\in \mathrm {V}\times {\mathbf {H}}^2$ replaced by $({\mathbf {u}},{\mathbf {v}})\in \mathrm {V}\times D(\mathrm {A})$.

We finally close this appendix with the following lemma.

### Lemma 6.4

There exist some positive constants $c_1$ and $c_2$ such that for any ${\mathbf {n}}_i\in {\mathbf {H}}^3$, i$=1,2$ we have, with $a=\frac{d}{4}$,

6.11 $$\begin{aligned} \begin{aligned} \Vert M({\mathbf {n}}_1)-M({\mathbf {n}}_2)\Vert&\le c_2\biggl (\Vert {\mathbf {n}}_1-{\mathbf {n}}_2\Vert _2 \Vert {\mathbf {n}}_1\Vert _2^{1-a} \Vert {\mathbf {n}}_1\Vert _3^a\\&\qquad + \Vert {\mathbf {n}}_1-{\mathbf {n}}_2\Vert ^{1-a}_2 \Vert {\mathbf {n}}_1-{\mathbf {n}}_2\Vert _3^a \Vert {\mathbf {n}}_2\Vert _2 \biggr ). \end{aligned} \end{aligned}$$

Note that we used the shorthand notation $M({\mathbf {n}}):=M({\mathbf {n}},{\mathbf {n}})$.

### Proof

From elementary calculi we infer the existence of a constant $C>0$ such that

$$\begin{aligned} \Vert M({\mathbf {f}}, {\mathbf {g}})\Vert \le C \Vert D^2 {\mathbf {f}}\Vert \Vert \nabla {\mathbf {g}}\Vert _{{\mathbf {L}}^\infty }+C \Vert \nabla {\mathbf {f}} \Vert _{{\mathbf {L}}^4}\Vert D^2 {\mathbf {g}}\Vert _{{\mathbf {L}}^4}. \end{aligned}$$

Owing to the embedding (6.3) it is not difficult to check that

$$\begin{aligned} \Vert M({\mathbf {f}}, {\mathbf {g}})\Vert \le C \Vert {\mathbf {f}}\Vert _2\biggl (\Vert \nabla {\mathbf {g}}\Vert _{{\mathbf {L}}^\infty }+ \Vert D^2 {\mathbf {g}}\Vert _{{\mathbf {L}}^4}\biggr ). \end{aligned}$$

Owing to (6.1) and (6.4) and the embedding (6.3) we obtain that

6.12 $$\begin{aligned} \Vert M({\mathbf {f}}, {\mathbf {g}})\Vert \le C \Vert {\mathbf {f}}\Vert _2 \Vert {\mathbf {g}} \Vert ^{1-a}_2 \Vert {\mathbf {g}} \Vert ^a_3, \quad a=\frac{d}{4}. \end{aligned}$$

Now, note that

$$\begin{aligned} M({\mathbf {n}}_1)-M({\mathbf {n}}_2)=M({\mathbf {n}}_1-{\mathbf {n}}_2,{\mathbf {n}}_1)+M({\mathbf {n}}_2,{\mathbf {n}}_1-{\mathbf {n}}_2). \end{aligned}$$

From this last identity and (6.12) we easily deduce the inequality (6.11). This ends the proof of Lemma  6.4. $\square$
